# Quantum-Classical Protocol for Efficient Characterization of Absorption Lineshape and Fluorescence Quenching upon Aggregation: The Case of Zinc Phthalocyanine Dyes

**DOI:** 10.1021/acs.jctc.3c00446

**Published:** 2023-08-29

**Authors:** Mohammad Aarabi, Daniel Aranda, Samira Gholami, Santosh Kumar Meena, Frederic Lerouge, Yann Bretonniere, Ilke Gürol, Patrice Baldeck, Stephane Parola, Fabienne Dumoulin, Javier Cerezo, Marco Garavelli, Fabrizio Santoro, Ivan Rivalta

**Affiliations:** † Dipartimento di Chimica Industriale “Toso Montanari”, Universitá degli Studi di Bologna, Viale del Risorgimento 4, I-40136 Bologna, Italy; ‡ Consiglio Nazionale delle Ricerche, Istituto di Chimica dei Composti Organo Metallici (ICCOM-CNR), I-56124 Pisa, Italy; § Instituto de Ciencia Molecular (ICMol), Universidad de Valencia, Catedrático J. Beltrán 2, 46980 Paterna, Valencia, Spain; ∥ Department of Chemical Engineering, Indian Institute of Technology Ropar, Rupnagar, 140001 Punjab, India; ⊥ ENSL, CNRS, Laboratoire de Chimie UMR 5182, 46 Allée d’Italie, 69364 Lyon, France; # TÜBITAK Marmara Research Center, Materials Technologies, Gebze, 41470 Kocaeli, Türkiye; ¶ Department of Biomedical Engineering, Faculty of Engineering and Natural Sciences, Acibadem Mehmet Ali Aydinlar University, 34752 Istanbul, Türkiye; ∇ Departamento de Química and Institute for Advanced Research in Chemical Sciences (IAdChem), Universidad Autónoma de Madrid, 28049 Madrid, Spain

## Abstract

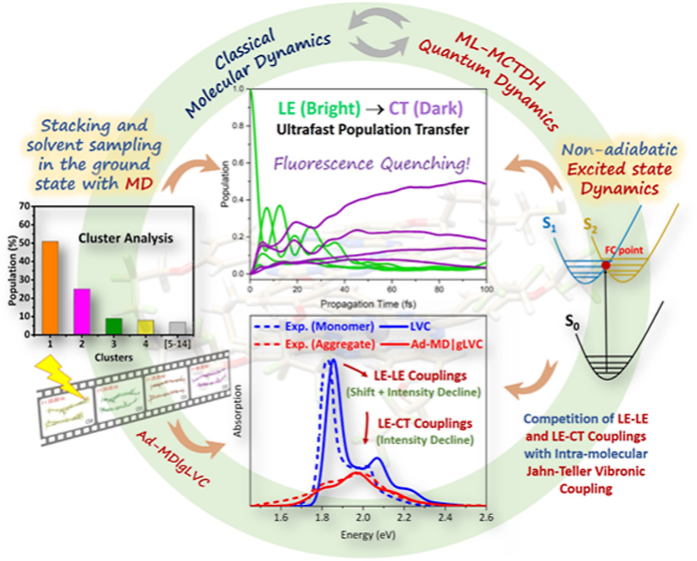

A quantum-classical protocol that incorporates Jahn–Teller vibronic coupling effects and cluster analysis of molecular dynamics simulations is reported, providing a tool for simulations of absorption spectra and ultrafast nonadiabatic dynamics in large molecular photosystems undergoing aggregation in solution. Employing zinc phthalocyanine dyes as target systems, we demonstrated that the proposed protocol provided fundamental information on vibronic, electronic couplings and thermal dynamical effects that mostly contribute to the absorption spectra lineshape and the fluorescence quenching processes upon dye aggregation. Decomposing the various effects arising upon dimer formation, the structure–property relations associated with their optical responses have been deciphered at atomistic resolution.

## 1 Introduction

Zinc phthalocyanines (Zn Pcs), an important category of porphyrin dyes, exhibit unique optical, magnetic, and electronic properties that make them good candidates for a broad range of applications.^1−9^ Such a wide applicability originates from some intrinsic properties, especially a strong and intense absorption in the red region (600–800 nm) resulting from a largely extended π-conjugated ring, high and long-lived fluorescence, high thermal and chemical stability, and long exciton lifetime. Nevertheless, Zn Pcs show a tendency to aggregate in aqueous media due to hydrophobic interactions, which are largely affected by their substitution pattern, leading to optical properties that are largely structure-dependent.^10−12^ The aggregation strongly affects the photophysical and photochemical properties of Zn Pcs, resulting in broadening, shift, and loss of peak height intensity of the Q-band (>500 nm) in the absorption spectrum (also featuring a higher energy, Soret band), quenching in fluorescence intensity, loss of catalytic activity, and shortening of their triplet state lifetime.^12−19^

The driving forces in the self-assembly of Zn Pc chromophores are principally π–π electronic interactions, van der Waals forces, and, in particular cases, specific interactions such as hydrogen bonding, coordination of central metal ion with atoms in the ring’s substituents or with solvent molecules. While depending on the substitution pattern, the aggregation tendency of Zn Pcs in non-polar solvents, such as dimethyl sulfoxide (DMSO), is generally lower than that in aqueous solutions.^10−12,14,15,17,18,20^ As a consequence, manipulating the structure and the environmental effects may result in changes in the optical and spectral properties of the Zn Pc chromophores. In this context, theoretical simulations can provide valuable and unique insights to correlate the self-assembly of chromophores with their optical properties by providing structural information with atomistic resolution in conjunction with accurate characterization of the corresponding electronic structure.^21^

It is well established that spectral fingerprints of any multichromophoric system strongly depend on their structures, as traditionally explained by Kasha’s exciton theory.^22,23^ Accordingly, one can distinguish between H-type (face-to-face), J-type (head-to-tail), and the so-called oblique-type aggregates. The first two aggregated forms exhibit blue- and red-shifts, respectively, in the maximum absorption with respect to the monomer, while a band splitting in the absorption spectra of distorted H- or J-aggregate (oblique case) is usually observed as a result of the allowed transition to split excitonic states. Also, while H-aggregates are non-emissive in most cases,^24,25^ J-types could feature enhanced fluorescence.^17,26^

Kasha’s theory is capable of interpreting some general spectral changes observed in aggregates. Nevertheless, a comprehensive understanding of the structure–(optical)property relations requires more sophisticated computational modeling since other effects such as charge-transfer (CT) excitations, vibronic couplings, and nuclear motions due to the interaction with the environment generally play a fundamental role.^21,21,27,28^ In the specific case of the Q-band of Zn Pcs, the vibronic progression peaks in the spectrum of monomers could easily overlap with the typical bands arising from aggregates, making it difficult to accurately distinguish different factors contributing to the whole spectral lineshape.^29−31^

Several attempts have appeared in recent years addressing some of the above-mentioned effects in aggregated Zn Pcs. In particular, for Zn Pc aggregates in crystalline lattices, the contribution of CT states on the vibrationally resolved absorption spectra and exciton dynamics has been recently explored by Feng *et al.* by means of a model Hamiltonian of aggregates in a Zn Pc film.^32^ They found that appearance, energy splitting, and relative peak height intensity ratio of two absorption peaks in the Q-band region are essentially controlled by the participation of CT states (*i.e.*, the coupling between Frenkel-like and CT-like states), rather than the mixtures of H- and J-type aggregates, as generally assumed.^13,18^ The lineshape of the Q-band and the nature of the electronic transitions were found to be strongly dependent on the number of Zn Pc units in the aggregate and on their relative intermolecular distances,^32,33^ as well as on the presence of remote exciton transfer^33^ and on the different geometrical conformations related to the material polymorphism.^34^

In contrast to what happens in solid structures, where large nuclear motions are quite restricted, self-assembly in solution involves significant nuclear conformational fluctuations that can affect the aggregation and the associated spectroscopic properties and ultrafast nonadiabatic dynamics.^21,27,35−37^ In the case of Zn Pc aggregates, the microscopic origin of the Q-band lineshape (broadening and energy shifts) and of the fluorescence quenching and their correlations with conformational dynamics in solution remain unrevealed yet. Very recently, some of us have introduced a novel mixed quantum-classical computational protocol, named adiabatic molecular dynamics with a generalized linear vibronic coupling model^38^ (Ad-MD|gLVC), to account for inter-state coupling and conformational disorder effects on the spectroscopy of aggregates in solution. It combines extensive classical molecular dynamics (MD) simulations with quantum dynamics (QD) wave-packet propagations to treat systems with coupled electronic states. This protocol was applied to study vibronic absorption spectra and ultrafast nonadiabatic dynamics of the photoexcited perylene diimide dimer in acetonitrile and water solutions, accurately reproducing the changes in the vibronic features of the absorption spectrum upon aggregation and investigating the role of CT states in the spectra and the ultrafast photoinduced dynamics. Zn Pcs and their aggregates exhibit a number of specific features that make them particularly interesting for testing and improving the Ad-MD|gLVC methodology.

First, the parent un-substituted Zn Pc chromophore has *D*_4*h*_ symmetry and its two lowest excited states are degenerate, undergoing Jahn–Teller (JT) distortions. Ring’s substitution affects the symmetry but does not erase the couplings between the states. Notably, the impact of JT-like couplings on the optical and photophysical properties of Zn Pc dyes remains unknown. Second, the quasi-degenerate local states of Zn Pcs arise from the existence of quasi-degenerate virtual orbitals, which increase the number of possible CT states formed upon aggregation, and their role remains to be investigated. Finally, in this perspective, the origin of both the Q-band lineshape and the non-emissive character of some self-assembled Zn Pcs has not yet been fully clarified.

Here, we present a variant of the Ad-MD|gLVC protocol to investigate the interplay of intra-molecular (JT-like) and inter-molecular (exciton–exciton and exciton–CT) vibronic couplings together with thermal dynamical fluctuations on the spectroscopy and photoinduced dynamics of Zn Pcs aggregates in solution. The approach employed here extends the Ad-MD|gLVC method by describing the JT-like couplings in the monomeric units and also exploiting the advantages of MD cluster analysis^39^ to effectively reduce the computational cost. This approach, indeed, speeds up the step of treating the soft degree of freedoms to account for inter-molecular dynamical effects by substituting the steps of the LVC Hamiltonian generation (usually performed on a large number of MD snapshots) with calculations for just a few representative number of structures selected by clustering.

As a model system, here, we synthesized, characterized spectroscopically, and computationally modeled the **Zn PcF12-SH** compound, an asymmetrically substituted Zn Pc of the A3B type, bearing peripheral 2,2,3,3-tetrafluoropropyloxy substituents on three isoindole subunits, and a thiol function at the extremity of the hexyloxy chain on the fourth subunit. **Zn PcF12-SH** was designed for potential application as a dye in hybrid organic–inorganic systems because it features a −SH terminal group that can covalently bind to the metal surfaces of nanoparticles.^40−42^

## 2 Theoretical Methods

### 2.1 General Scheme of the Computational Protocol

**Figure 1:**
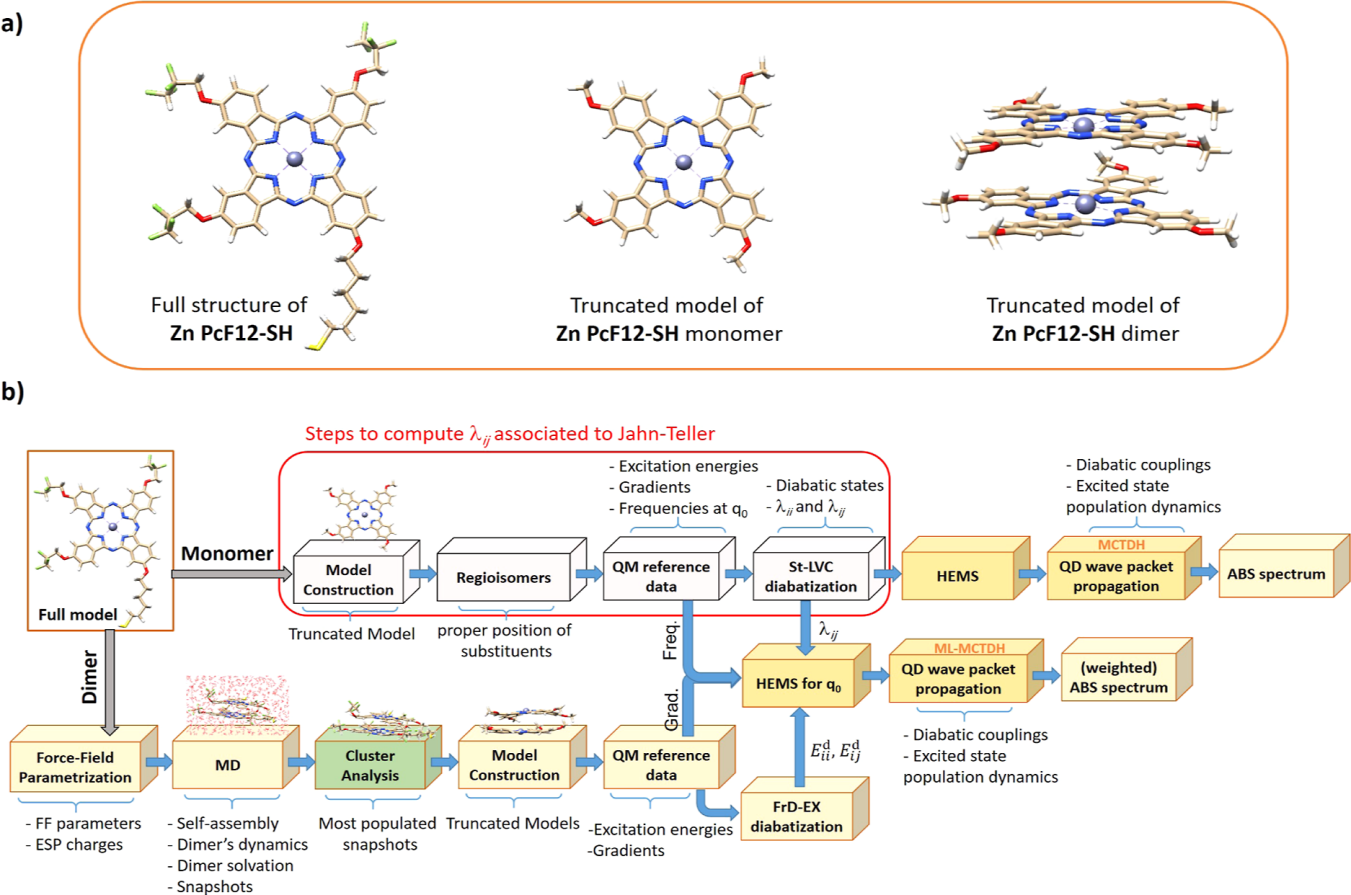
(a) Molecular structure of the **Zn PcF12-SH** chromophore studied in this work and its truncated models in the monomeric and aggregated states. (b) General scheme of the protocol used in the present study. The main computational steps are indicated in the boxes, while the main outputs of each step are indicated below the brackets. The steps to compute the linear coupling term associated with the JT effect are specified by the red box. HEMS: hierarchical effective mode selection, FrD-EX: fragment diabatization-based excitonic model, St-LVC: standard linear vibronic coupling model.

Figure 1a,b presents the molecular structure of **Zn PcF12-SH** and the stepwise computational scheme used in the present study, respectively. According to this protocol, the solute and solvent nuclear degrees of freedom of the system are adiabatically separated into the so-called stiff and soft modes, where the fast stiff modes, *i.e.,* the intra-molecular vibrations of the monomers, are described at the quantum dynamical (QD) level through wavepackets propagating on the coupled electronic states, while the slow soft modes, describing the solvent and the relative arrangement of the monomers, are treated classically by MD simulations.^37,38^ In practice, soft modes are considered too slow to react to the electronic transitions. Therefore, they are sampled with classical MD before the photoexcitation, but, afterward, they are considered frozen. On the other hand, the photoinduced dynamics and the electronic spectral shape do depend on the instantaneous configurations of the soft modes, because they affect the coupled potential energy surfaces (PES) on which the fast modes move (”static disorder limit”).^43^

Despite the robustness of the Ad-MD|gLVC protocol, the need to repeat the diabatization and the QD propagations for a large number of snapshots still demands a significant computational effort for large systems like **Zn PcF12-SH**. To overcome this problem, here we modify the original Ad-MD|gLVC protocol^38^ by including MD clustering (see 1b). Cluster analysis is a statistical data mining tool that seeks and explores the conformation heterogeneity in an ensemble of molecular structures generated by MD simulation trajectories and divides the snapshots into groups that share similar conformational states (*i.e.,* into clusters). Conventional cluster analyses of MD trajectories use cut-off parameters based, for instance, on the root-mean-square deviation (RMSD) between Cartesian coordinates.^44^ Initially, a thermodynamically accessible set of conformations of the solvated **Zn PcF12-SH** dimer is sampled through an MD trajectory of 50 ns, and a snapshot is extracted every 0.05 ns. Then, the snapshots with similar soft coordinates (*i.e.,* those with similar relative arrangement of the **Zn PcF12-SH** rings) are identified by MD cluster analysis and grouped into different clusters. Eventually, a representative snapshot, namely, the ”central structure,” which shares similar soft coordinates with other structures existing in a given cluster, is selected and then employed for the computations of vibronic spectra and nonadiabatic dynamics. The central structure (hereafter CS) is defined as the structure featuring the smallest average distance to all the structures existing in the same cluster.^44−48^ This approach reduces the number of the diabatizations (about 1 h for a single truncated model of the **Zn PcF12-SH** dimer on a single CPU processor) and subsequent QD wavepacket propagations (about 60 h for each bright electronic excited state of each single structure) for a large number of snapshots, to only a few selected ones determined by the cluster analysis, resulting in a huge speedup of the whole analysis. The final absorption can then be retrieved from a ”weighted average” of the nonadiabatic vibronic spectra computed in the stiff modes subspace for few representative MD snapshots, considering the population of each corresponding cluster. It should be noticed here that compacting the contribution of a large number of different snapshots to just a few leads to an underestimation of the inhomogeneous broadening effect, which is not a real issue for the present calculations, as shown in the following sections.

The computational scheme in Figure 1b also includes an additional route, specified by the red box, passing through a truncated model of the **Zn PcF12-SH** monomer for which only methoxy functional groups are retained in the structure (with the same truncation scheme used for dimers after selection of MD snapshots). This route is mainly designed here to evaluate the linear coupling term associated with the JT effect between the two nearly degenerate excited states of each individual **Zn PcF12-SH** unit involved in self-assembly. The latter path, in fact, additionally extends the capability of the Ad-MD|gLVC protocol,^38^ introducing the possibility to treat multichromophore systems, with possible intra-site internal conversions, while taking into account inter-state coupling and inter-molecular structural distortion effects. A similar extension was already employed to study the formation of CT states in guanine–cytosine in explicit solvent models.^49^

### 2.2 LVC Model

We parameterize the following LVC Hamiltonian considering a set of coupled electronic states in the diabatic basis |**d**⟩ = (|*d*_1_⟩, |*d*_2_⟩, ..., |*d*_*n*_⟩)
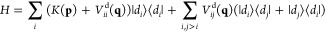
1which depends on the dimensionless normal mode coordinates **q** of the ground electronic state S_0_ and their conjugate momenta **p**. The kinetic *K* and potential *V* terms of the Hamiltonian are defined as
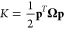
2
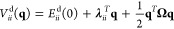
3
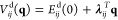
4

Here, **Ω** is the diagonal matrix of S_0_ normal mode frequencies ω_α_, *E*_*ii*_^d^(0) is the diabatic vertical energy of state *i*, and *E*_*ij*_^d^(0) is a constant electronic coupling between diabatic states *i* and *j*, both at the reference geometry (0). The latter term does not appear on the standard LVC approach^50^ (St-LVC), and it is introduced to describe aggregated states in terms of local excitations (LEs) and CT states.^51^ The vectors **λ**_*ii*_ and **λ**_*ij*_ (with *j* ≠ *i*) are the gradients of the diabatic PESs in the reference geometry and the linear coupling parameters, respectively. The adiabatic–diabatic transformation is obtained by a recently developed fragment diabatization (FrD) approach.^51−53^ For this reason, in previous work, we defined the obtained LVC Hamiltonian as FrD-LVC.^51^ Here, for the sake of brevity, we will keep on using the simplified label LVC. According to this approach, we defined diabatic states of the truncated **Zn PcF12-SH** dimer on the basis of reference states that are either the adiabatic states of the isolated fragments/monomers of the dimer (for LEs) or one-electron transitions between orbitals on different fragments (for CT states). See the Supporting Information for details on computation of the LVC parameters.^54^

The diabatic states |*L*⟩ and |*L*′⟩ for the monomer were defined identical to the two lowest-energy adiabatic states. They are different combinations of H → L and H → L + 1 transitions, where H and L stand for HOMO and LUMO orbitals, respectively. For the dimer, eight diabatic states |*d*_*i*_⟩ should be defined since L and L + 1 orbitals are nearly degenerate. Therefore, we had to consider not only the four bright LEs |*L*_1_⟩, |*L*_1_^′^⟩, |*L*_2_⟩, and |*L*_2_^′^⟩ (“1” and “2” identify the two different monomers) but also the CT states |CT(1 → 2)⟩, |CT′(1 → 2)⟩, |CT(2 → 1)⟩, and |CT′(2 → 1)⟩, where *i* → *j*, *i*, and *j* indicate the monomer where a hole is created (in the HOMO) and the electron is transferred, respectively. More specifically, the electron is moved in L in |CT(1 → 2)⟩ and |CT(2 → 1)⟩ and in L + 1 in |CT′(1 → 2)⟩ and |CT′(2 → 1)⟩ (see Figure S1).

Obtaining the linear intra-(**λ**_*ii*_) and inter-(**λ**_*ij*_) coupling terms is computationally demanding for systems of the size of a Zn Pc dimer. These terms, in fact, are computed through the numerical differentiation of the diabatic potential energies *V*_*ij*_^d^, (*j* ≥ *i*) at structures displaced by a small quantity ±Δ_α_ along each normal coordinate *q*_α_. Therefore, we followed simplified schemes allowing us to avoid a normal-mode analysis of the dimer at each snapshot, and the related difficulties on performing it at a structure out-of-equilibrium, thus updating only the state vertical energies *E*_*ii*_^d^(0) and constant couplings *E*_*ij*_^d^(0). It is worthy to recall that in our vibronic models, we need to describe only the internal motion of each monomer since in our protocol the inter-molecular vibrations describing the conformation of the dimer are treated as classical coordinates sampled by MD simulations. We started our analysis with the intra-state **λ**_*ii*_ couplings, *i.e.,* the gradients (***g***_*i*_) of the diabatic states, describing the displacement of the equilibrium positions, and we followed the recipe proposed and tested in ref (38). In practice, we assumed that the normal coordinates of the dimer (**q**_dim_) are identical to those of two independent monomers. Hence, the **q**_dim_ was represented as a column vector including the normal coordinates of the two individual monomers, namely, **q**_dim_ = {**q**_1_, **q**_2_}. Accordingly, for all structures of the dimer, the gradients of the LEs were approximated as **g**_1_ = {**g**, **0**}, **g**_1_^′^ = {**g**^′^, 0}, **g**_2_ = {**0**, **g**}, and **g**_2_^′^ = {0, **g**^′^} for the states |*L*_1_⟩, |*L*_1_^′^⟩, |*L*_2_⟩, and |*L*_2_^′^⟩, respectively, where **g**, **g**′ are the gradients of the |*L*⟩ and |*L*′⟩ states, respectively, and were computed analytically through the isolated monomer (see the scheme shown in Figure 1b) accounting for solvent effects in the non-equilibrium regime. For the CT states, we adopted the gradients **g**_+_, **g**_–_, and **g**_–_^′^ obtained from the cationic and anionic species with the electron either in L or L + 1 orbitals for **g**_–_ and **g**_–_^′^, respectively. Therefore, the gradients of the CT states become **g**(CT(1 → 2)) = {**g**_+_, **g**_–_}, **g**(CT′(1→2)) = {**g**_+_, **g**_–_^′^} and so forth. Under this approach, the linear coupling terms computed numerically on the truncated **Zn PcF12-SH** monomer were approximated as the representative sets of  and , to be used in dimer’s route, as shown in Figure 1b.

As far as inter-state couplings **λ**_*ij*_ are concerned, in ref (38), we showed that the constant term in eq 4 dominates the linear terms. On these grounds, here, we set **λ**_*ij*_ = **0**, and hence, *V*_*ij*_^d^(*q*) = *E*_*ij*_^d^(0). This choice allows a large computational time saving by skipping the necessity to repeat calculations at displaced geometries and reducing the FrD diabatization to a simpler excitonic (EX) one, which we indicated in a previous study as ”FrD-EX” approach.^53^ The only exception was the coupling between the states affected by JT, |L_*i*_⟩ and |L_*i*_^′^⟩, for which the constant term  is zero and, therefore, the linear coupling is the leading term, *i.e.,*. In this case, we used for all structures of the dimer the **λ**_*LL*′_ vectors calculated from the full St-LVC parameterization of the isolated monomer (with *N* = 213) to evaluate the impact of JT couplings on the spectral shapes (see Supporting Information for more details on the evaluation of JT linear coupling terms).

In summary, these simplifications greatly reduce the computational cost of the method, making it affordable for systems as large as the Zn Pc dyes.

### 2.3 Adiabatic and Nonadiabatic Computation of the Absorption Spectra and Wave Packet Quantum Dynamics

The molar absorptivity ϵ(ω) at zero kelvin can be computed in a time-dependent framework by the following expression
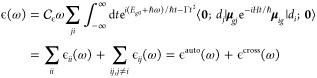
5where  collects all the physical constants (with full expression given in the Supporting Information), **μ**_*gj*_ = ⟨*g*|**μ**|*d*_*j*_⟩ represents the electric transition dipole moment between states *g* and *j* and **0** is the ground-vibrational state of the ground electronic state *g*, whose energy is set to zero. Since the diabatic states are defined to be ideally independent of the nuclear coordinates, the vector **μ**_*gj*_ can be safely considered constant (Condon approximation). The time integrals contain a Fourier transform with a quadratic Gaussian damping , ruled by a parameter Γ corresponding to a Gaussian broadening in the frequency domain and the correlation functions ϕ_*ji*_(*t*) = ⟨*d*_*j*_; **0**| exp −*iHt*/*ℏ*|*d*_*i*_; **0**⟩, which can be separated in two terms: auto-(*i* = *j*) and cross-(*i* ≠ *j*) correlation functions. The latter functions are required to obtain the terms ϵ_*ii*_(ω) and ϵ_*ij*_(ω) in eq 5 and computed according to the procedure described below. It is worth noting that the auto-correlation functions carry the total intensity, while the cross terms simply modulate the spectral shape without changing the total value of its integral. The total vibronic spectrum is then obtained by Fourier transform of the total correlation function, as detailed in the Supporting Information.

For the LVC Hamiltonian, the time integrals in eq 5 can only be solved numerically by propagating the initial wave packets |*d*_*i*_; **0**⟩ on the coupled PESs. To this end, here, we adopted the multi-configurational time-dependent Hartree (MCTDH) method, as implemented in the Quantics package.^55^ Notwithstanding the impressive methodological progress thanks to the recent advances on MCTDH and its multi-layer extension ML-MCTDH,^56^ including all the degrees of freedom for a system of the Zn Pc dimer’s size is extremely challenging. To overcome this technical problem, we used a hierarchical representation by blocks of the LVC Hamiltonian in terms of effective coordinates (the hierarchical effective mode selection, HEMS, see Figure 1b) in such a way that the short-time dynamics, which dominates the spectral shape, can be described by few blocks of coordinates (see Supporting Information for details).^57−61^ Considering the truncated **Zn PcF12-SH** monomer and neglecting the JT-like couplings, its spectra can be obtained with an adiabatic Born–Oppenheimer computation. In the adiabatic approximation, the correlation functions in eq 5 have an analytical solution, whose evaluation represents a very convenient way to obtain fully converged spectra. To perform these computations, we adopted the vertical gradient (FC|VG) and adiabatic Hessian (FC|AH) approaches^62^ implemented in the code .^63,64^ The availability of these analytical correlation functions also allowed us to investigate effects neglected in the LVC Hamiltonian, namely, those arising from quadratic differences in the excited- and ground-state PESs (causing the so-called Duschinsky mixing of the normal modes) and the relative difference of their vibrational frequencies.

After computing the nonadiabatic spectrum for each representative Zn Pc dimer taken from different clusters, the final expression for the ”weighted spectral line shape” is
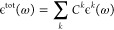
6and
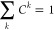
7where *C*^*k*^ stands for the weight (or population portion) of the relevant cluster *i*, where the nonadiabatic vibronic spectrum ϵ^*k*^ is computed for its representative snapshot.

## 3 Experimental and Computational Methods

### 3.1 Synthesis

#### 3.1.1 Materials and Methods

Elemental analyses were obtained using a Thermo Finnigan Flas 1112 instrument. Infrared spectra were recorded on a PerkinElmer Spectrum BX FT-IR system. ^1^H NMR spectra were recorded in CDCl_3_ solution on a Bruker or Varian 500 MHz spectrometer. Matrix-assisted laser desorption/ionization time-of-flight mass spectrometry (MALDI-TOF-MS) measurements were performed on a Bruker Daltonics micrOTOF. Positive ion and linear mode MALDI-TOF MS spectra of the compounds were obtained in 2,5-dihydroxy benzoic acid MALDI matrix using nitrogen laser accumulation 50 laser shots. The substituted 4-nitrophthalonitrile,^65^ 4-[(6-hydroxyhexyl)oxy]phthalonitrile,^66^ and 4-(2,2,3,3-tetrafluoropropoxy)phthalonitrile^67^ were synthesized and purified according to the literature procedures. *N*,*N*′-Dimethylformamide and *n*-amyl alcohol were dried as described by Perrin and Armarego^68^ before use. All zinc acetate, K_2_CO_3_, anhydrous Na_2_SO_4_, 1,5-diaza-bicyclo[4.3.0]non-5-ene, CDCl_3_, and 2,2,3,3-tetra-fluoro-1-propanol were purchased from commercial suppliers. Further details and a synthesis scheme (Scheme S1) are available in the Supporting Information.

### 3.2 Optical Properties’ Measurements

Absorption spectra were recorded on a JASCO V670 spectrophotometer, employing a quartz cell with an optical path of 2 cm. Excitation and fluorescence emission spectra were recorded using a Horiba-Jobin Yvon Fluorolog-3 spectrofluorimeter equipped with a Hamamatsu R928 or water-cooled R2658 photomultiplier tubes. Spectra were corrected for the intensity variations of both the excitation light source (lamp and grating) and the emission spectral response (detector and grating). To record the absorption spectrum of monomeric **Zn PcF12-SH**, a 50 μL stock solution of 7 μM **Zn PcF12-SH** in DMSO was initially prepared and then diluted by adding 300 μL of DMSO to reach the concentration of 1 μM. To obtain the absorption spectrum of aggregate **Zn PcF12-SH**, the same initial stock solution was adopted and diluted by adding 300 μL of water. The absorption spectrum of **Zn PcF12-SH** in this aqueous solution is not stable over time as a decrease of the diffusion background was observed by the time evolution of the diluted solution, leading to a 30% loss of oscillator strength. This could be due to the mixing of solvents or, alternatively, due to the generation of **Zn PcF12-SH** aggregates that disappear over time by sedimentation or by slow dissolution. The major change in absorption was observed within 30 min after addition of water. Thus, we reported here the spectrum recorded 24 h after adding water to the mother solution, showing negligible changes over time.

### 3.3 Computational Details

Since the full control of the positions of the functional groups is experimentally challenging, in order to define a reference model for **Zn PcF12-SH**, we studied the optical properties of its possible regioisomers using, to this scope, the truncated model described above.^69^ One regioisomer has been selected as the representative monomer, following the procedure described in the Supporting Information (see Section S3.2), and used for all computational procedures described in this work. A full structure (full model) of the **Zn PcF12-SH** monomer was built using the substitution pattern on this selected regioisomer in order to compute its absorption spectrum and to study its dynamics in solvent. For the parameterization of the force field (FF) and atomic charge calculations required to run classical MD simulations, as sketched in Figure 1b, the full model geometry was optimized in solution (in both aqueous solution and pure DMSO) with density functional theory (DFT) using the B3LYP^70,71^ exchange–correlation functional, the 6-31G(d,p) basis set,^72,73^ the Grimme’s D3 dispersion corrections,^74,75^ and a polarizable continuum model (IEF-PCM),^76^ as implemented in Gaussian16.^77^ The FF parameters for the **Zn PcF12-SH** molecule were obtained by the procedure followed in ref (78), where bonded and van der Waals parameters were taken from the GROMOS parameter set.^79^ Moreover, the atomic partial charges were obtained using the electrostatic potential fitting method.^80,81^ All excited-state computations were performed using a time-dependent DFT approach (TD-DFT) and the CAM-B3LYP^82^ functional for a reliable description of CT states. As discussed in Section 2.2, in order to model the gradients of the CT states of the dimer, we had to compute the excited-state gradients of the radical anion species. This was done analytically for the truncated model, employing the “maximum overlap method” (MOM)^83−85^ (employing the same DFT functional and basis sets), which offers a single-determinant approximation for excited states. To include the solvent effects on the excited-state properties, all the calculations were carried out adopting the linear-response PCM approach (LR-PCM) for solvation in both aqueous solution and pure DMSO. All the DFT/TD-DFT calculations were performed using the Gaussian16 package,^77^ while the MOM computations were performed with the Q-Chem software package.^86^ Diabatizations were carried out with the code Overdia.^54^

All the MD simulations have been performed using the GROMACS package^45−48^ (version 5.1.4), initiated adopting the two well-separated (>20 Å) **Zn PcF12-SH** units in pure DMSO and aqueous solution, for which the details of the model systems are reported in Table S2 of Supporting Information. Periodic boundary conditions were applied in all three directions. A constant temperature of 300 K was maintained by the Berendsen scheme. A time step of 2 fs was also employed, and trajectories’ frames were stored every 50 ps (*i.e.,* one snapshot every 0.05 ns). Energy minimization was performed after the addition of water. The system was first equilibrated in the *NVT* ensemble for 100 ps. Then, the MD production runs were performed for at least 50 ns for each solvent in the same *NVT* ensemble (see Table S2 of the Supporting Information for details).

In order to get a first “strongly-interacting” model for the **Zn PcF12-SH** dimer, at the end of the 50 ns dynamics in water, a **Zn PcF12-SH** dimer conformation was extracted and a truncated model was generated. This structure was then optimized at the CAM-B3LYP/6-31G(d,p) level (namely, the ”optimized truncated dimer”, hereafter OM) and its excited states were characterized with the same exchange–correlation functional within the LR-PCM/water implicit solvation model. Starting from this structure, we constructed another opposite dimer model (namely, the “distant truncated dimer”, hereafter DM) where the distance between two monomer rings, *i.e.*, *d*_P–P_, was rigidly increased by 1.55 Å, to assure that the rings were at a distance much larger than those observed for **Zn PcF12-SH** dimers in the MD simulation, keeping the rest of structure the same as the OM one. The *d*_P–P_ was defined as an average interatomic distance along the axis normal to the Zn Pc ring, with atoms belonging to lateral chains being excluded. The definition of this interatomic distance was necessary because the two aromatic macrocycle rings of **Zn PcF12-SH** dimers are not necessarily fully planar and generally exhibit some out-of-plane distortions.

Along the MD trajectory in water, we observed the **Zn PcF12-SH** aggregation in the ∼2–50 ns range, and in this time interval, we performed cluster analysis by employing the g_cluster tool implemented in GROMACS^45−48^ and considering only the macrocycle rings since their relative orientation and distance are relevant for determining the **Zn PcF12-SH** dimer photophysics. The structurally similar clusters were obtained with the GROMOS clustering algorithm as described by Daura *et al.*,^44^ adopting a 0.10 nm RMSD cut-off. This provided four dominant clusters incorporating 93% of the total trajectory frames. Finally, the CS of each cluster was selected as the representative dimeric conformation, and the lateral chains were truncated to generate the corresponding truncated dimer models. The resulted structures were directly employed to generate the LVC Hamiltonians following the simplified approach described in Section 2.2 (see Figure 1b).

Regarding the QD simulations, the nuclear wavepackets were propagated according to the LVC nonadiabatic Hamiltonian for 100 fs, employing the MCTDH and ML-MCTDH methods, respectively, for the truncated models of monomer and the dimers of **Zn PcF12-SH** (with specific settings detailed in the Supporting Information). Finally, the nonadiabatic spectra were computed with a Gaussian broadening of HWHM = 0.04 eV.

## 4 Results and Discussion

### 4.1 Absorption Spectroscopy of **Zn PcF12-SH** Monomers and the Jahn–Teller Effect

**Figure 2:**
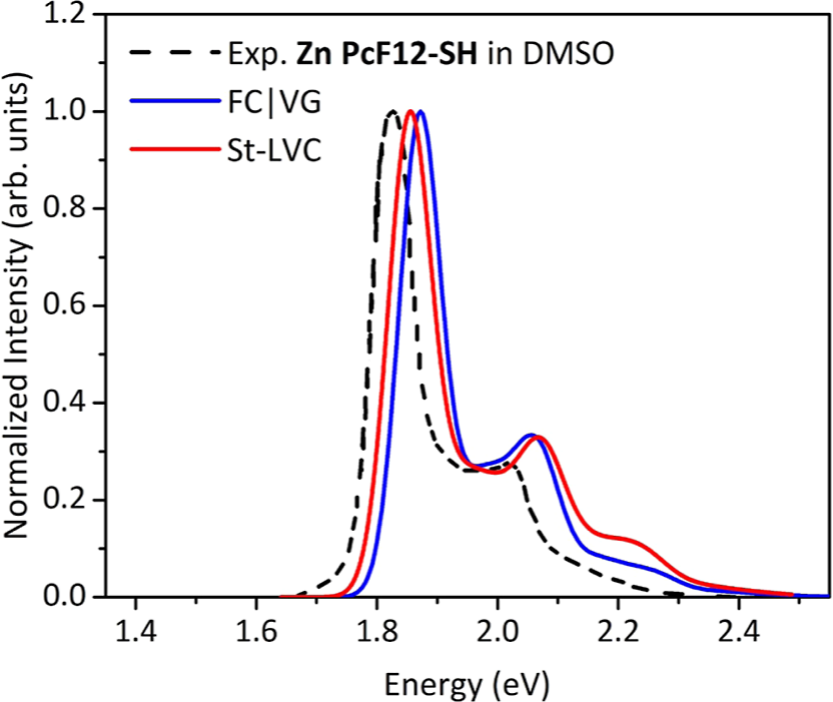
Comparison of the experimental absorption spectrum recorded for diluted solutions of the **Zn PcF12-SH** compound in pure DMSO in the Q-band region (dashed black line), with the theoretical spectra computed for the truncated model (regioisomer C) with adiabatic FC|VG (blue line) and nonadiabatic St-LVC (red line, including JT coupling) approaches. To facilitate the comparison, all the spectra were normalized at the maximum peak height intensity of the main peak. No shift has been applied to the theoretical spectra. All vibronic transitions were convoluted with a Gaussian function with HWHM = 0.04 eV.

The characteristic Q-band of Zn Pc monomers lies in the 1.65–2.25 eV (550–750 nm) range, peaking at 1.83 eV (679 nm) for our **Zn PcF12-SH** in pure DMSO, as shown in Figure 2, where monomeric species are expected to be largely predominant. The Q-band is associated with ππ* electronic transitions associated with two almost degenerate (S_1_ and S_2_) electronic states. This band clearly features a vibronic progression with peaks at higher energies, eventually overlapping with very minor contributions from the absorption of aggregated **Zn PcF12-SH** species as indicated by the recorded experimental excitation spectra (see Figure S13 in the Supporting Information). As a first step, we aimed to obtain a model of the Zn Pc monomers that reproduces the features of the experimental absorption spectrum of the **Zn PcF12-SH** compound in pure DMSO. Since Zn Pcs are known to feature several possible regioisomers when isoindole subunits are monosubstituted (see Figure S7 in the Supporting Information for details), we investigated the effect of regioisomers on their absorption properties. Given the large number of permutations associated with different substitution patterns on the Zn Pc ring, we could perform such an extensive study only on the truncated model of **Zn PcF12-SH**, as described in the Supporting Information. The outcome showed that the standard deviations of vertical excitation energies and oscillator strengths among 36 Zn Pc regioisomers are <0.02 eV and <0.025 with averages of 1.92 eV and 0.712, respectively (see Table S5 in the Supporting Information), indicating a minor effect of the substitution positions on the fundamental absorption properties of the monomers. Therefore, in order to compute the full vibronic spectra of these monomers, we employed the FC|VG method limiting to few regioisomers. Indeed, considering the precursors used in the synthesis of the **Zn PcF12-SH** compound, regioisomers carrying lateral substituents at the α positions can be discarded, restricting the analysis to just four regioisomers (namely, A, B, C, and D, see the Supporting Information for details). These isomers showed slightly different vibronic spectra that still all hold the main features of the experimental absorption spectrum of the **Zn PcF12-SH** compound in pure DMSO (see the Supporting Information for details). Therefore, among these four regioisomers, we selected one, namely, the isomer C. Its vibronic spectrum indeed was the most similar to the average spectrum of the four A–D isomers (see the Supporting Information for details). Moreover, the absorption spectrum of isomer C was also best agreeing with the experimental spectrum of **Zn PcF12-SH** in pure DMSO, featuring a small, rigid blue shift of ∼0.05 eV, as shown in Figure 2. If one constructs a “full” model out of this regioisomer by adding the complete lateral chains, the vibronic spectrum remains almost unaffected, except for an extra rigid blue shift of just ∼0.02 eV (see Figure S10 in the Supporting Information). Notably, the isomer C features the largest mixing of H → L and H → L + 1 transitions in the ππ* (S_1_ and S_2_) excited states among all regioisomers, thus being also the best model to investigate the potential JT effect on the absorption properties of the Zn Pc monomers.

Thus, we investigated the impact of JT coupling on the spectral shape of the monomer (represented by the regioisomer C) by adopting the St-LVC Hamiltonian approach. In Figure 2, the St-LVC (accounting for JT coupling effects) and the FC|VG spectra of the truncated model in adiabatic approximation are compared, showing high similarity. Only a minor difference can be noticed for the relative peak height intensity of the highest energy peak of the vibronic progression, overall indicating that the effect of JT coupling is not large enough to modify the lineshape of the absorption spectrum of **Zn PcF12-SH** monomers.

### 4.2 Absorption Spectroscopy of **Zn PcF12-SH** Dimeric Species

**Figure 3:**
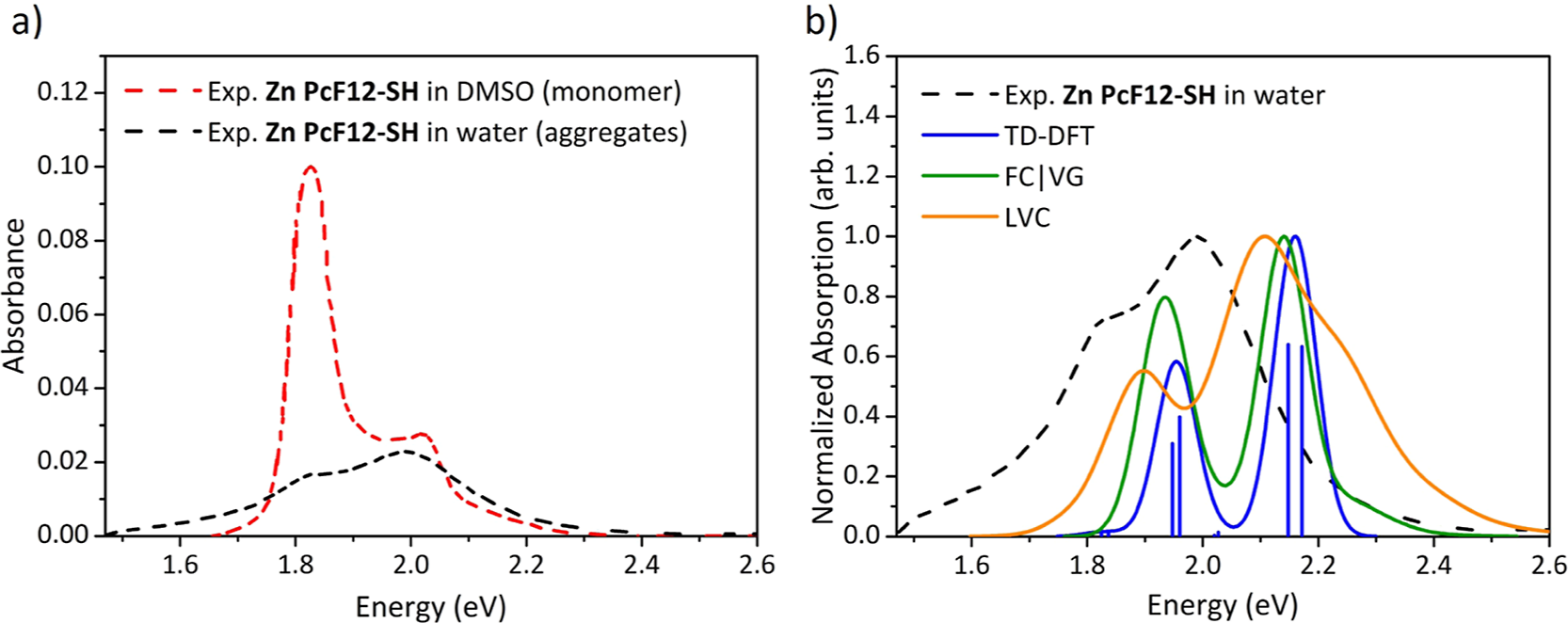
(a) Experimental absorption spectra recorded for diluted solutions of **Zn PcF12-SH** in pure DMSO (dashed red line) and aqueous solution (dashed black line) in the Q-band region, which stand, respectively, for the monomeric and aggregate forms of Zn Pc. (b) Comparison of the theoretical absorption spectra computed for the OM dimer model of Zn Pc at different adiabatic (pure electronic TD-DFT and vibronic FC|VG) and nonadiabatic vibronic (LVC) approaches, with the experimental spectrum recorded for diluted solution of the **Zn PcF12-SH** in aqueous solution in the Q-band region. To facilitate the comparison, the theoretical spectra were normalized to match the maximum peak height intensity. All transitions were convoluted with a Gaussian function with HWHM = 0.04 eV.

As shown in Figure 3a (and Figure S8 in the Supporting Information), the comparison of the experimental absorption spectra recorded for **Zn PcF12-SH** in aqueous solution and in pure DMSO reveals remarkably different optical behaviors in different media. As compared to DMSO, absorption in water is associated with a dramatic drop of the peak height intensity of the Q-band leading to the formation of a broad band extending from 1.46 to 2.48 eV (500 to 850 nm) with two main peaks with comparable intensities. Finally, in aqueous solution, a blue shift of the maximum of Q-band can be noticed. All these observations suggest the formation of H-type aggregates according to Kasha’s exciton theory.^22^ Similar findings have been reported in the literature for other Zn Pc derivatives as well.^10,12,13,31−34^ The formation of H-type aggregates for the **Zn PcF12-SH** compound is further supported by our experiments since this Zn Pc in aqueous solution does not emit. Indeed, the compound exhibits fluorescence at 1.81 eV (685 nm, see Figure S8 in the Supporting Information) and a small Stokes shift of 0.015 eV (∼6 nm in this region) only in DMSO, both characteristic features of monomeric Zn Pc species.^12,14^ In the following sections, we will report the absorption properties of the H-aggregates of the **Zn PcF12-SH** compound in water by adopting the TD-DFT, FC|VG, and the LVC approaches to determine the spectral properties of reference models of dimer conformations (*i.e.,* OM and DM), and the Ad-MD|gLVC protocol combined with cluster analysis to characterize the specific effects of conformational fluctuations in solution.

#### 4.2.1 Absorption Properties of the Zn Pc Dimer Reference Models

We start our analysis by neglecting the effect of thermal fluctuations and adopting simplified Zn Pc dimer models. As a first reference model, we used the OM dimer (as described in Section 3.3) that represents the equilibrium structure of the truncated dimer in water solution and it is associated with monomers that are stacked more tightly than what is observed in the MD simulations of the solvated **Zn PcF12-SH** compound.

Figure 3b shows the pure electronic absorption spectrum (in the Q-band region) obtained for the truncated **Zn PcF12-SH** dimer at the TD-DFT/CAM-B3LYP level and compares it with corresponding vibronic spectra in both adiabatic (FC|VG) and nonadiabatic (LVC) approximations as well as with experiment. The main features of the experimental spectrum lineshape are well reproduced at the pure electronic TD-DFT level. In particular, the experimental double peak (at 1.82 and ∼2 eV) is reproduced with reasonable energy separation, and the relative peak height intensity of the lowest energy peak is consistently smaller than the higher energy one. In terms of excitation energies and absorption maxima, the TD-DFT spectrum shows a non-negligible blue shift of 0.17 eV, with respect to the experimental spectrum of **Zn PcF12-SH** in aqueous solution (see Figure 3b). This is, however, in line with the presence of more tightly stacked monomers in the OM dimer than in the real **Zn PcF12-SH** system (as it will be showed for the full model in solution discussed in the next section). An inspection of the vertical electronic transitions computed at the TD-DFT level for the OM dimer (see Table S6 in the Supporting Information) reveals eight low-lying excited states, exhibiting a pairwise quasi-degeneracy. In other words, they constitute four nearly degenerate pairs of electronic transitions, two pairs of which are dipole-allowed, thus bright (see sticks in Figure 3b), while the rest are dipole-forbidden, thus dark. Accordingly, in terms of excitations between the delocalized orbitals of the dimer, the two low-energy S_3_ (and S_4_) bright states in the pure electronic absorption spectrum originate from combinations of two equally weighted H – 1 → L(L + 1) and H → L + 1(L) excitations, while the two high-energy S_7_ (and S_8_) bright states come from combinations of two H – 1 → L + 2(L + 3) and H → L + 3(L + 2) excitations, in which the former transitions have higher weights than the latter ones (see Figure S14 in the Supporting Information for picture of molecular orbitals involved). Thus, although the TD-DFT computations appear to be able to provide a reasonably good absorption spectrum compared to experimental data, the complicated composition of the electronic transitions at this pure electronic level makes a comprehensive understanding of the nature of the states difficult. Moreover, the TD-DFT adiabatic approach essentially ignores important contributions like nuclear motions and excited-state couplings, influencing spectral lineshape. Remarkably, the eight lowest excited states of the Zn Pc dimer appear in a narrow energy window of 0.35 eV at the TD-DFT level (see Table S6 in the Supporting Information), a situation where inter-state nonadiabatic couplings may operate, calling for a full nonadiabatic treatment beyond the adiabatic Born–Oppenheimer approximation. To this end, as detailed in Section 2.2, we defined eight diabatic states in the localized basis following the FrD diabatization approach. The resulting LVC Hamiltonian in diabatic representation (data in Table S7 of the Supporting Information) contained four LE and four CT diabatic states and their mixing well-reproduce the eight lowest adiabatic TD-DFT states (see the comparison of energies in Tables S6 and S9 of the Supporting Information). The outcome of the FrD diabatization reveals strong LE–LE and LE–CT couplings that are also remarkably large considering the corresponding energy gaps. Hence, one can expect them to contribute to the absorption spectra. In fact, as shown in Figure 3b, the nonadiabatic vibronic LVC spectrum features a broader spectrum with respect to the TD-DFT one using the same phenomenological broadening (HWHM = 0.04 eV), while the relative intensities of the two main bands remain almost the same. The adiabatic vibronic FC|VG computations (considering the bright states S_3_, S_4_, S_7_, and S_8_) predict a spectrum slightly broader than TD-DFT and narrower than LVC. Thus, the experimental relative intensities of the main bands appear more similar to those of LVC spectrum than TD-DFT and FC|VG ones. Overall, these results suggest that inter-state couplings, included in the LVC method, are likely the main contributions to the spectral broadening associated with aggregation.

#### 4.2.2 Modulation of Spectral Lineshapes by Inter-state Couplings upon Aggregation

**Figure 4:**
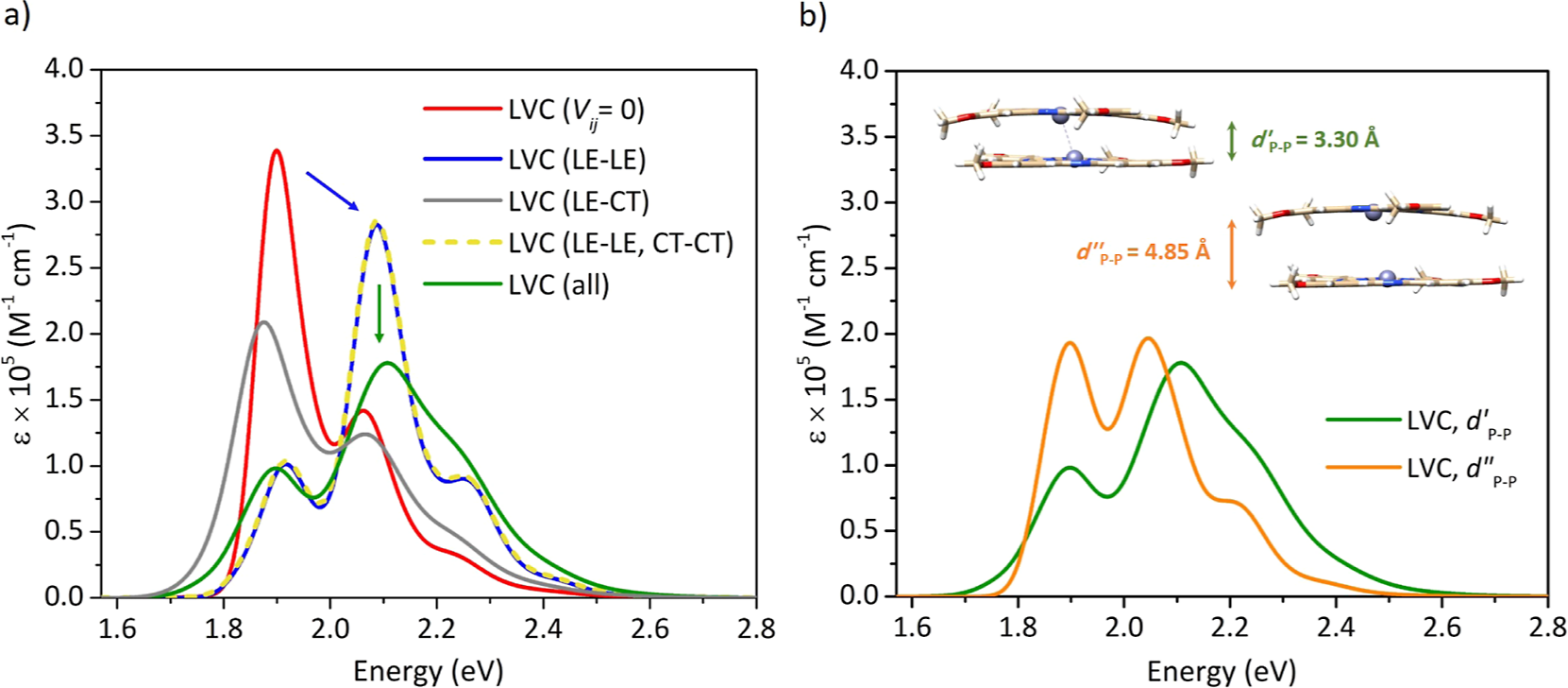
(a) The nonadiabatic vibronic spectra computed for the OM dimer model of **Zn PcF12-SH** turning off various coupling terms; without inter-state coupling (*V*_*ij*_ = 0, red line), included only the four LE states and neglecting the CT states (blue line), included only LE–CT couplings and excluded LE–LE and CT–CT ones (gray line), included LE–LE and CT–CT couplings and neglected LE–CT ones (yellow line) as well as the one accounting for all kinds of couplings between the four LE and four CT diabatic states (green line). (b) Comparison of the nonadiabatic vibronic spectra computed for the OM dimer (green line) with that of the DM dimer (orange line), where all the four LE and four CT diabatic states were included in the computation of LVC spectra. No shift has been applied to the computed spectra, and all vibronic transitions are convoluted with a Gaussian of HWHM = 0.04 eV.

In order to capture which excited-state couplings contribute predominantly on the spectral line shape upon aggregation, we started from an idealized model of the truncated **Zn PcF12-SH** dimer in which all inter-state couplings are removed, (*i.e.V*_*ij*_ = 0), which provides a spectrum identical to that obtained with the FC|VG adiabatic model for the monomer,^62^ and then we progressively switched on selected couplings in the LVC Hamiltonian (see Table S7 in the Supporting Information), as shown in Figure 4a. Including the couplings between the four LE states (LE–LE), while still setting those involving CT states to zero, leads to a dramatic change of the relative peak height intensity of the first two vibronic peaks (*i.e.,* the main difference changing the lineshape while going from the monomer to the aggregate), resulting in a blue shift of the spectrum maximum by 0.13 eV. The maximum intensity also decreases with respect to *V*_*ij*_ = 0, resulting in a broader spectrum, getting most of the main features of the LVC spectrum with all inter-state coupling terms included. On the contrary, the activation of just the LE–CT couplings (LE–CT) has a much smaller (still non-negligible) effect on the spectral shape since it does not revert the relative intensity of the two lowest-energy peaks. At the same time, as expected, activating also the CT–CT couplings (*i.e.,* LE–LE and CT–CT) does not lead to any change with respect to the LE–LE spectrum since CT states can only change the spectrum mixing with LE ones. Finally, LE–CT couplings do have an impact and, in fact, their inclusion on top of the (LE–LE, CT–CT) case, which leads to the full LVC spectrum (all), determines a remarkable redistribution of the relative intensities of the vibronic bands, further decreasing the peak intensity height of the most intense band and improving the agreement with the experimental spectrum of aggregated **Zn PcF12-SH** in aqueous solution. These results evidence the remarkable effect of inter-state couplings, clarifying that the blue shift associated with Zn Pc aggregation and the lineshape broadening is mainly driven by exciton interactions among LE states, as expected in H-type aggregates, whereas however LE–CT couplings are not negligible and are responsible for a significant modulation of the relative intensities of the vibronic bands. This outcome suggests a partially different interpretation of the specific roles of inter-state couplings in Zn Pc aggregates with respect to what has been observed for symmetric Zn Pc thin films,^32−34^ where modulation of band intensities upon aggregation was mainly ascribed to the contribution from LE–CT state coupling (thus, not due to LE–LE couplings) and the number of molecular units in the aggregate. It is worth mentioning that the latter effect has been partially investigated in the present work by the TD-DFT/CAM-B3LYP simulation of the absorption spectrum of a trimer species, as reported in the Supporting Information (see Figure S15). The computed spectrum for Zn Pc (while missing vibronic and electronic coupling contributions) showed that trimeric species could contribute to the absorption spectrum by altering the relative intensities of the absorption spectrum bands and by adding new peaks with small intensity height in the low-energy portion of the Q-band. These results are in line with the trend observed for previously reported data in the Zn Pc thin film.^33^

In order to further explore the effect of inter-state coupling on the spectral shape as related to the distance between two Zn Pc monomers, we also computed the LVC nonadiabatic vibronic spectrum of the DM dimer (defined in Section 3.3), as shown in Figure 4b. For this structure, while the exciton coupling between the LE states is greatly diminished, the contribution of the CT states is naturally eliminated since they now stand much higher in energy and are not able to couple with the LEs (see Table S8 in the Supporting Information for the relevant LVC Hamiltonian). Interestingly, the LVC spectrum of the truncated **Zn PcF12-SH** dimer with “separated” monomers exhibits similar features of the spectrum of the monomer, with just different relative intensities of the two most intense bands. This outcome confirms that the lineshape of the aggregated **Zn PcF12-SH** spectrum is dominated by inter-state couplings that are correlated, as expected,^32,33^ with the relative distance between the interacting monomers. To provide insights into the nature of the electronic transitions involved in the LVC absorption spectra upon aggregation, we compared the LVC adiabatic states of the OM dimer, obtained by diagonalization of the LVC Hamiltonian at the FC point (**q** = 0), against those of the DM dimer (see Table S9 in the Supporting Information). When the monomers are well separated, the Zn Pc dimer exhibits two nearly degenerate electronic transitions with considerable oscillator strengths (*i.e.,* bright), which are essentially constructed from contributions of LE states, while adiabatic LVC states with CT nature are essentially dark (almost zero oscillator strength). As two monomers approach together, the energy gaps between states with LE and CT characters substantially decrease (by ∼0.2–0.3 eV) and two nearly degenerate (high-energy) states with CT character feature now considerable oscillator strengths. The analysis of composition of the adiabatic LVC states reveals that all adiabatic LVC states in the aggregated dimer hold a large mixing of LE–CT characters (generally about 50/50 in percentage). As a consequence, also the oscillator strengths of the low-energy states are altered (*i.e.,* almost halved), while their energies do not undergo any sizeable change. This clearly describes the key role played by inter-state LE–CT couplings in modulating the nature of electronic states upon Zn Pc aggregation. Finally, it is noteworthy mentioning that the Zn Pc dimer models considered so far do not account for an accurate description of the conformations of **Zn PcF12-SH** aggregated dimers in solution (which can feature Zn Pc units with various sliding positions and relative distances) and thus can serve as a reliable reference for more realistic modeling, as discussed in the next section.

#### 4.2.3 Interplay between Inter-state Couplings and Dynamical Effects on the Absorption Spectroscopy of the Zn Pc Dimer

**Figure 5:**
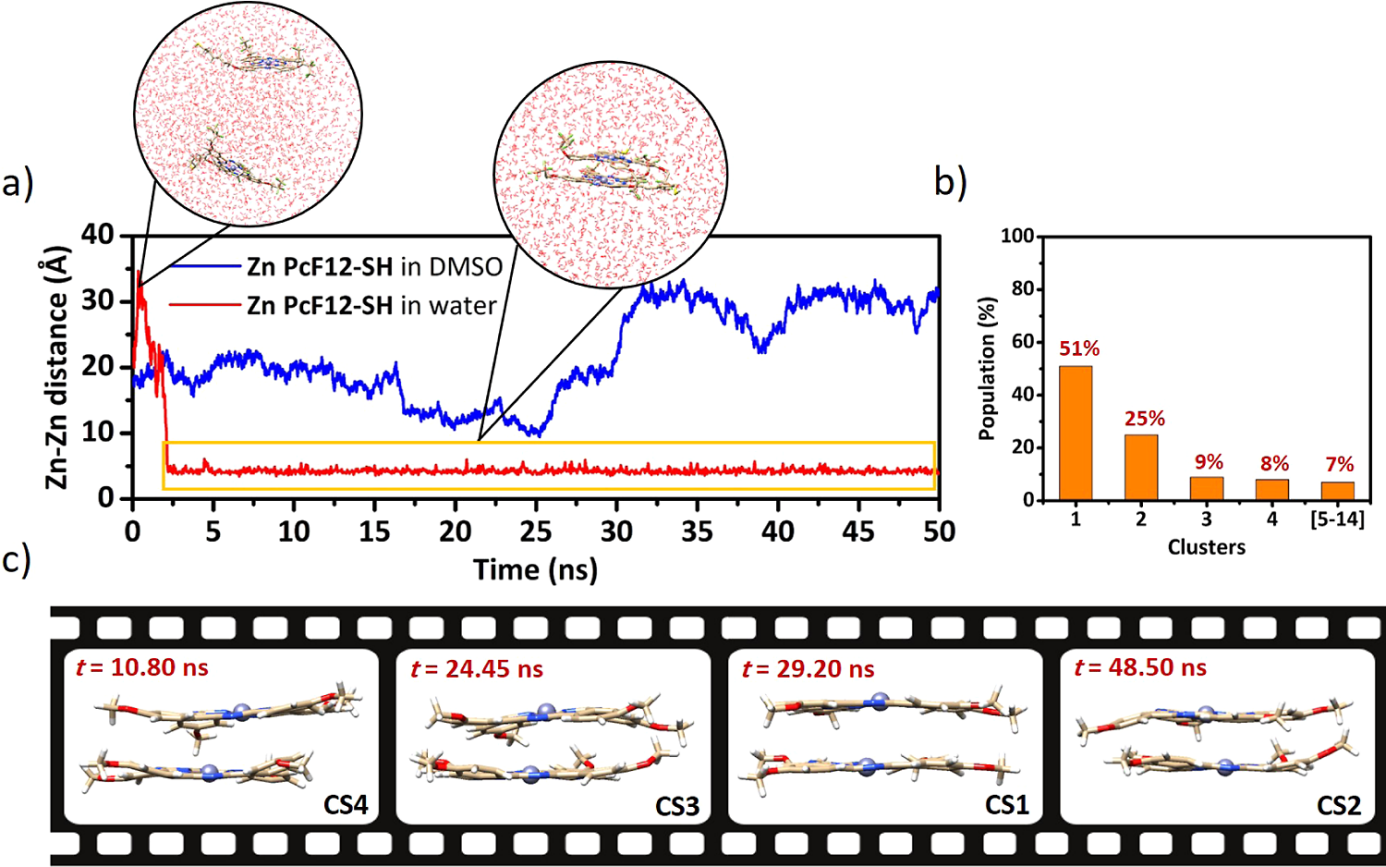
(a) Time evolution of the Zn–Zn distance (*d*_Zn–Zn_, in Å) between the two interacting **Zn PcF12-SH** units along the 50 ns MD trajectories in pure DMSO (blue line) and in water (red line). The segment of MD trajectory in water considered for cluster analysis is specified by a yellow box. (b) Population of various clusters obtained from MD clustering with an RMSD of 0.1 nm. (c) Representative Zn Pc dimer structures associated with CS1 to CS4.

In this section, in order to account for the effects of structural dynamics, we discuss the Ad-MD|gLVC spectrum of the **Zn PcF12-SH** dimer in aqueous solution. First, to investigate the behavior of Zn Pc molecules in different solvents, we performed a 50 ns MD simulation, adopting the full structure of the **Zn PcF12-SH** dimer (full model) solvated in both pure DMSO and water. Although the thiol group terminated and the other lateral chains have shown only a marginal impact on the monomer optical properties, in the case of aggregates they could play a significant role by influencing the stability and organization of the stacked forms, thus eventually affecting the final spectral shape. Figure 5 summarizes the analysis of these MD simulations, showing the time-dependent fluctuations of the distance between two **Zn PcF12-SH** monomers (in particular the distance between two Zn atoms) in pure DMSO and in water, along with the outcome of the cluster analysis for water-solvated dimers. Indeed, as evident from the fluctuations of the Zn–Zn distances, two **Zn PcF12-SH** monomers aggregate in water within 2 ns (with Zn–Zn distance dropping below 4.5 Å) and remain in contact for the entire 50 ns trajectory, while the dimer formation is not observed in pure DMSO, with Zn–Zn distance being always >10 Å along the entire trajectory (see Figure 5a). These results are in line with the experimental evidence of aggregation in aqueous solution and not in pure DMSO, providing atomistic details of the possible geometric arrangements of the **Zn PcF12-SH** dimer in aqueous solution. As shown in the inset of Figure 5a, two stacked **Zn PcF12-SH** molecules generally arrange as face-to-face in water, implying formation of H-aggregates, in line with the experimental optical properties observed in aqueous solution.

The portion of MD trajectory in water related to **Zn PcF12-SH** aggregation (from ∼2 to 50 ns, see Figure 5a) was considered to sample the soft coordinates associated with the possible conformations of the Zn Pc dimer. The snapshots with dimers sharing similar conformations (*i.e.,* similar soft coordinates) were then grouped by cluster analysis, with the first four largest clusters holding 93% of total trajectory frames, as depicted in Figure 5b. The CSs of these four clusters (depicted in Figure 5c) were considered as representative conformations of the **Zn PcF12-SH** dimer in water solution in order to compute the corresponding Ad-MD|gLVC spectrum, according to eq 6.

**Figure 6:**
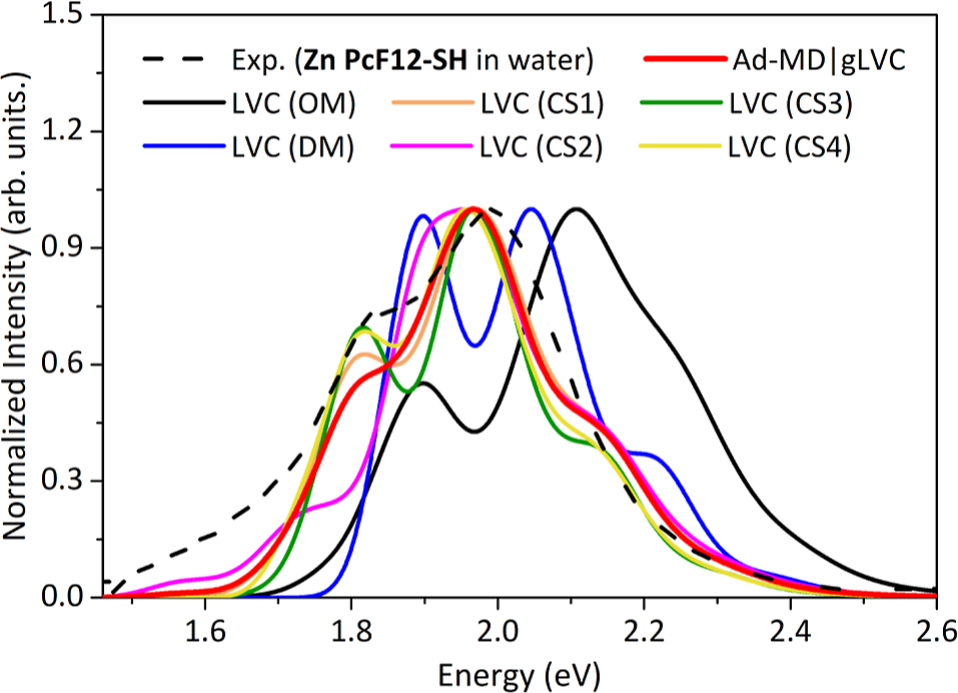
Weighted Ad-MD|gLVC spectrum and the individual LVC spectra computed using four truncated Zn Pc dimers obtained from the cluster analysis of MD trajectory of **Zn PcF12-SH** in water. For comparison, the LVC spectra computed for the “static” (OM and DM) models are also reported. To facilitate the comparison, all the spectra were normalized at the maximum intensity (height) of the main peak. No shift has been applied to the theoretical spectra.

Figure 6 shows the individual LVC spectra computed for the four CSs and the corresponding weighted average Ad-MD|gLVC spectrum, as compared with the experimental spectrum in aqueous solution and the “static” (OM and DM) dimer models described above. All computed nonadiabatic LVC spectra exhibit two main peaks that are red-shifted with respect to the LVC spectrum of the OM model, being closer to the experimental spectrum in aqueous solution. Notably, the pure electronic adiabatic TD-DFT spectra (see Figure S16 in the Supporting Information) of these four CSs are not in agreement with experiments, implying the important role of the inter-state couplings and vibronic contributions. Therefore, these effects need to be accounted for if one wants to reproduce the experimental spectrum out of specific and realistic conformations extracted from MD simulations. The comparison of the individual LVC spectra clearly revealed the effects of structural dynamics on the spectral shape, involving changes in terms of broadening, relative spectral spacing, and intensity of the absorption peaks (height). Interestingly, while the position of the maximum absorption for the most intense band is almost unaffected by the conformational state considered (peaking always at ∼2.04, against ∼2.00 eV in the experimental spectrum), the spectrum of the CS2 features, if compared with other conformations: (i) a larger broadening of the high-energy band; (ii) a red-shift and peak height intensity decrease of the low-energy band (at ∼1.80 eV for CS1, 3–4); and (iii) the appearance of the lowest-energy weak band (below 1.60 eV), also present in experimental spectrum. The “weighted” Ad-MD|gLVC absorption spectrum, computed by averaging over these four LVC spectra and taking into account the weights of each corresponding cluster, features two main bands lying at energies that match the experimental ones. In this spectrum, it is worth noting that the contribution of CS2 increases the overall broadening and the peak height intensity of the absorption at low energies (below 1.8 eV). Comparing the LVC spectra obtained for the “static” OM and DM models with those of the four conformations selected from MD simulations, it appears clear that just rigidly increasing the inter-monomer distance from one extreme (3.30 Å for the OM model) to the other (4.85 Å for the DM model) explains the alternation of the relative intensities of the two bands and captures most of the broadening observed in the experimental spectrum of the aggregates, part of which must be due to the presence of aggregates larger than just dimers. It is expected, also considering our TD-DFT simulated spectrum for a trimeric species mentioned above, that larger aggregates would increase the relative intensity height of the red tail of the Q-band while featuring an overall broader and lower band with respect to dimers. Anyway, our results indicated that the modulation of the band intensity height and the broadening in the low-energy tail that characterizes the experimental spectrum could be rationalized only by including the contributions from the conformational dynamics. As already discussed in Section 2.1, replacing the contribution of a large number of snapshots with four structures from cluster analysis, remarkably speeds up the computation, but at the cost of a limited quantitative prediction of the spectral broadening. On one side, we showed above that the effect of inter-state couplings leads to a spectral width comparable to experiment even for static structures of the dimer, and the broadening effect due to the fluctuations of the slow degrees of freedom is partially accounted for with the phenomenological Gaussian width (*i.e.* HWHM = 0.04 eV), but, on the other hand, the effects of such solvent inhomogeneous broadening are not fully captured by our approach. In particular, the position of CT states is expected to be especially sensitive to the instantaneous position of the solvent molecules and, in the previous sections, we have highlighted that CT states contribute to the width and shape of spectrum. Neglecting solvent inhomogeneous broadening on CT states, thus, might be involved in the underestimation of the low-energy tail of the spectrum that is found in the averaged spectrum shown in Figure 6.

Since we showed that the experimental absorption spectral features of the **Zn PcF12-SH** aggregated structures in solution are affected by the inter-state couplings and the structural dynamical conformations, we could now obtain a detailed microscopic understanding of the correlations between these two effects. Here, thus, we rationalize the correlation between the parameters of the diabatic Hamiltonian (*i.e.*, diabatic energies and the couplings) and the variation of the geometrical parameters in the selected structures obtained from the MD trajectories. In other words, we are interested in the geometrical parameters that contribute the most to the spectral lineshape of the solvated **Zn PcF12-SH** dimer, with particular attention to the CS2 structure that differentiates the most from the other selected CSs. To this end, we first defined the most relevant inter-monomer coordinates (carrying all the soft degrees of freedom of the system), including the Zn–Zn distance (*d*_Zn–Zn_), the inter-ring distance (*d*_P–P_, computed as explained in Section 3.3) as well as the angles (α_*i*_ and β_*i*_, with *i* = 1–8) defined according to reference axes within each Zn Pc ring (see details and Figure S17 in the Supporting Information) and monitored their distributions along the MD trajectories in water (see Figures S18–S20 in the Supporting Information). In particular, since the angles α_*i*_ describe both tilting and displacement of one monomer with respect to the other in all directions, they are correlated with the *d*_Zn–Zn_ distance, while the dihedral angle β describes the twisting of the two Zn Pc units.

The *d*_Zn–Zn_ distance shows a distribution in the range of 3.6–5 Å, with a broad maximum in the 3.95–4.35 Å range, thus much larger Zn–Zn distances than that of the OM model (with *d*_Zn–Zn_ = 3.08 Å). It is worth noting that while dispersion corrections involved in the geometry optimization of OM might predict smaller distances for π-stacking with respect to the FF employed in the MD simulations, the large difference in *d*_Zn–Zn_ distances observed is essentially due to the absence of the long lateral chain in the reduced OM model. The distribution of the angles α_*i*_ (defined in the range 0–90°) exhibited a non-negligible population at 90° for each angle, but perfectly π-stacked monomers would imply all α_*i*_ angles being simultaneously at 90°, a condition that is not found along the MD simulation (see Figure S21 in the Supporting Information). The twisting angle β showed a distribution from −40 to 5° with a maximum falling into the range of −15 to −20°. This indicates that the twisting of the two stacked **Zn PcF12-SH** monomers in solution is remarkably restricted due to the presence of the lateral chains.

**Figure 7:**
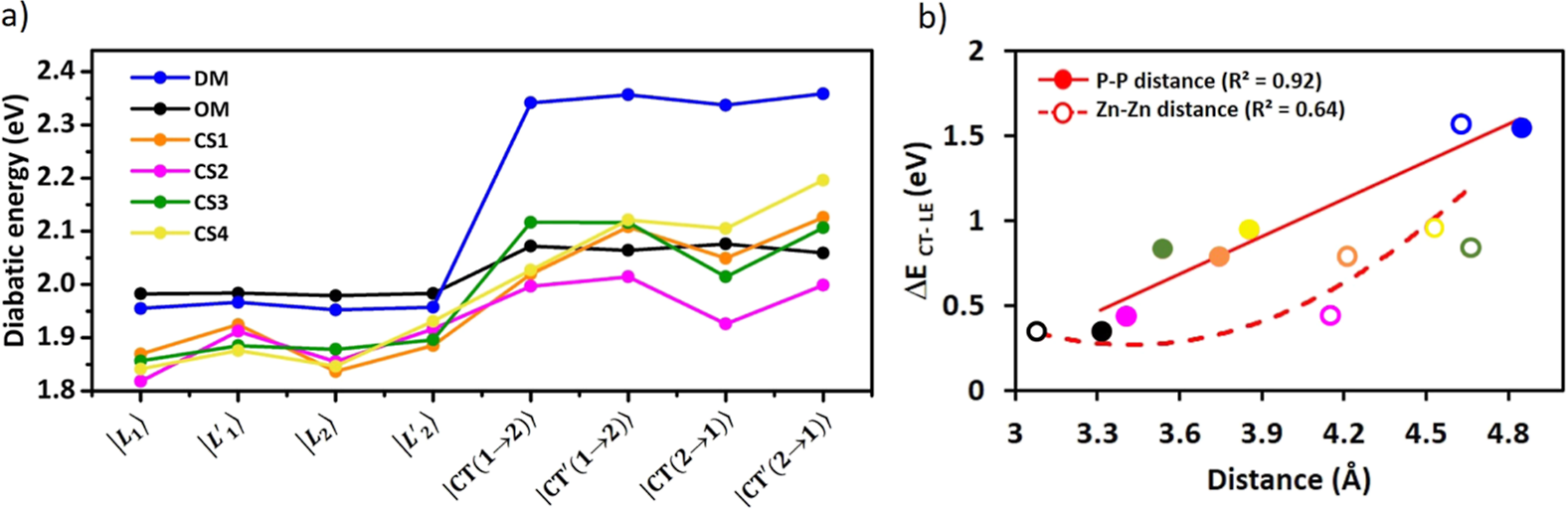
(a) Stability of the LE and CT diabatic states (in eV) for all Zn Pc dimers considered in this study. (b) Dependence of the averaged CT–LE energy gap on both *d*_Zn–Zn_ and *d*_P–P_ stacking distances.

**Figure 8:**
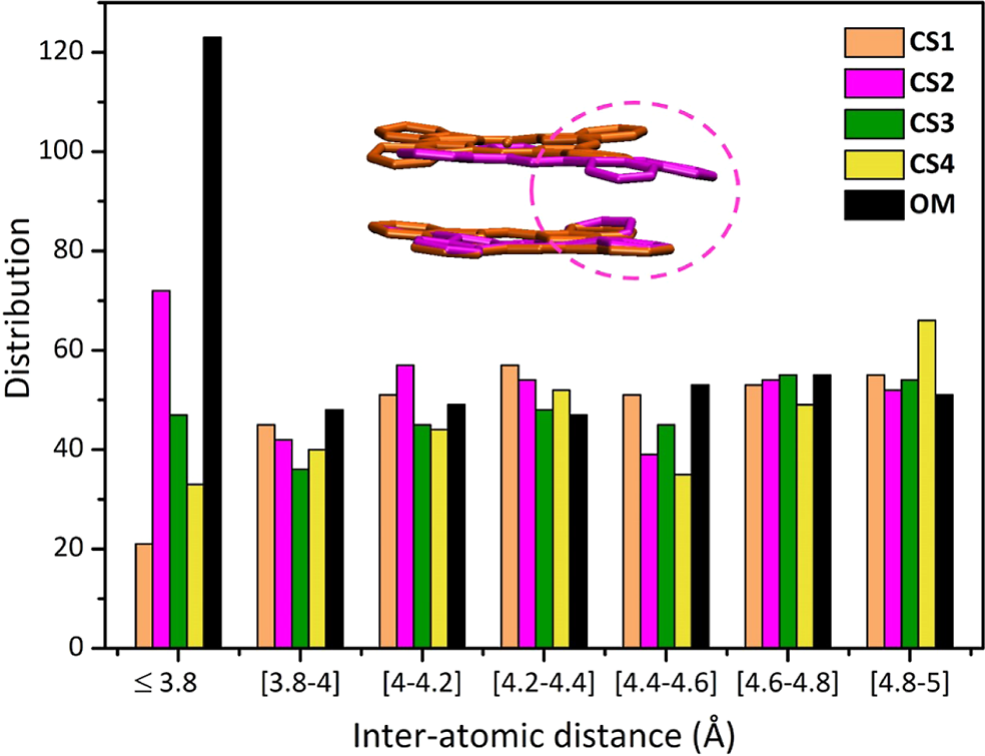
Comparison of distribution of inter-atomic distances for the representative Zn Pc dimer structures associated with CS1 to CS4, as well as for the OM dimer. Only the distances ≤5 Å are reported for the sake of clarity.

Figure 7a compares the relative energies of LE and CT diabatic states for all Zn Pc dimeric structures considered in this study (with their geometrical parameters reported in Table S10 in the Supporting Information). Interestingly, all diabatic LE states are nearly degenerate in both the OM and DM Zn Pc dimers, while such degeneracy is lost for all CSs taken from the MD clustering, as a result of the dynamical geometrical distortions in solution. The same holds for the CT states, where the dynamical effects are even larger. As mentioned above, the LE–CT energy gaps change significantly going from the OM to DM dimer model (*i.e.,* changing the *d*_P–P_ distance) along with their mixing and, consequently, their couplings. In order to confirm that this trend holds in the structures extracted from the MD simulations, we correlated the LE–CT energy gaps with both the *d*_P–P_ and *d*_Zn–Zn_ distances for all the Zn Pc dimers considered in this study, including the CSs of the four main clusters, as shown in Figure 7b. The LE–CT energy gaps generally reduce with the decrease of the *d*_P–P_ distance, and with a good linear correlation (with *R*^2^ = 0.92), while for *d*_Zn–Zn_, such a trend is not observed. Notably, CS2, which showed unique spectral properties with respect to other dimeric structures (see Figure 6), features the smallest LE–CT energy gaps and the shortest *d*_P–P_ distance with respect to the other CSs from MD. However, the *d*_Zn–Zn_ distance in CS2 is not much shorter (only by 0.06 Å) than that of CS1 (see Table S10 in the Supporting Information). This suggests that the peculiar spectroscopic features of this Zn Pc dimer must be related to the relative orientation of the two monomers in the CS2 structure. Interestingly, the β dihedral angle in this structure (∼−20°) is similar to that of the OM model and also to that of CS3, indicating that the twisting angle is not the critical geometrical parameter. Instead, the *d*_P–P_ distance of CS2 is similar to that of the OM model (3.39 *vs* 3.30 Å, respectively), while the *d*_Zn–Zn_ distance is much larger (4.15 *vs* 3.08 Å, respectively, see Table S10 in the Supporting Information). Since *d*_P–P_ is an average interatomic distance along a specific axis (*i.e.,* that normal to the Zn Pc ring), this suggests that this distance might remain short despite a long *d*_Zn–Zn_ distance if, for instance, one side of the Zn Pc ring bends toward the other monomer. In fact, looking at the distribution of the interatomic distances among the atoms in the rings, a peculiar behavior for CS2 can be clearly observed. As shown in Figure 8, indeed, CS2 features a large number of interatomic distances below 3.80 Å, still smaller than that of the OM model (this because the latter features also a quite small *d*_Zn–Zn_) but significantly higher than those of the other clusters. Thus, the peculiar absorption spectrum of CS2 is mainly due to a specific tilting of the two Zn Pc rings, where the two monomers are not significantly sliding from each other, promoting the approach at one side of the rings (see structural overlay in Figure S22 in the Supporting Information) and increasing the orbital overlap in a specific portion of the Zn Pc dimer. This type of configuration, thus, allows a decrease of the LE–CT energy gap (see Figure 7a), and then the effectiveness of the corresponding inter-state couplings is also expected to increase.

The full analysis of inter-state couplings as a function of the *d*_P–P_ and *d*_Zn–Zn_ distances for all the Zn Pc dimers considered in this study (see Figures S23–S28 in the Supporting Information) reveals that the excitonic (LE–LE) couplings are minimally affected by the geometrical configuration (except in the obvious case of very distant monomers), while the LE–CT couplings, indeed, exhibit large variation among different Zn Pc dimers. This suggests that the LE–CT couplings are the most sensitive to the geometrical variations of the aggregate, resulting as the main cause of the thermal fluctuations effects on the observed spectral shape. Notably, the particular spectral shape of CS2 is found to be correlated with the presence of quite large couplings of specific LE–CT states (see Figures S25–S28 in the Supporting Information), which result to be even higher than those computed for the OM model (despite the latter has shorter *d*_P–P_ and *d*_Zn–Zn_ distances). In order to directly correlate the effects of the structural dynamics on the coupling terms to the spectral shape, we compared the vibronic LVC spectra for CS dimers (in addition to the OM dimer model), isolating the contributions from each type of inter-state couplings (see Figure S29 of the Supporting Information for details). With respect to the spectra computed in the absence of inter-state couplings (*V*_*ij*_ = 0), *i.e.,* analogous to monomer-like spectrum with the low-energy band holding the highest peak height intensity, the inclusion of LE–LE modulates the relative intensities of the two main bands for all structures. Notably, a sizeable effect of the LE–CT couplings could be observed only in the case of CS2 and OM, *i.e.,* those structures with small *d*_P–P_ distances, and it results into a spectral broadening (particularly large for CS2) and a decrease of the peak height intensity for the low-energy band.

In the next section, we will show how the correlated effects of inter-state couplings and conformational dynamics do determine not only the absorption properties of the Zn Pc dimers but also their emissive properties.

### 4.3 Quantum Dynamics of the Electronic Populations

**Figure 9:**
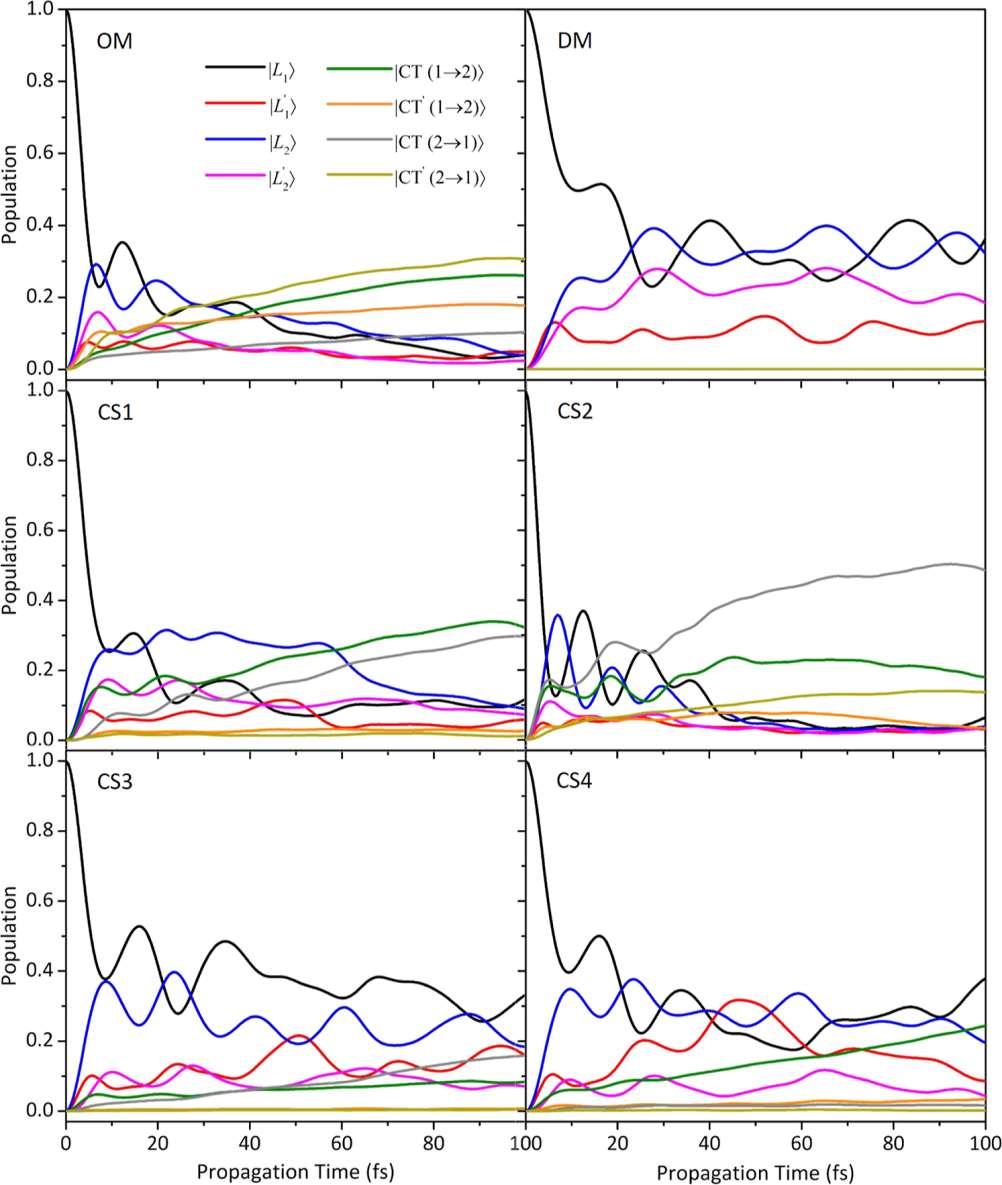
Population dynamics of the diabatic states of all the Zn Pc dimers considered in this study, starting photoexcitation from |*L*_1_⟩. ML-MCTDH including 30 effective coordinates.

Beyond allowing the computations of nonadiabatic vibronic spectra, the QD propagations driven by the LVC Hamiltonian give access to the nonadiabatic time evolution of the photoexcited system on the ultrafast timescale. It is noteworthy that, despite the fact that the LVC model is built on a diabatic basis, it describes also population transfers between adiabatic states and the possible effects of avoided crossings and conical intersections.^87^ In Figure 9, we have compared the nonadiabatic QD electronic populations initiated on the local |*L*_1_⟩ state for all the Zn Pc dimers considered in this study. The time-evolution of the electronic populations following individual photo-excitation from |*L*_1_^′^⟩ has been also computed (and reported in Figure S30 in the Supporting Information). It should be highlighted that it can be difficult to design an experimental setup able to photoexcite selectively a single LE of Zn Pc dimers. Notwithstanding this, we start discussing the results of these simulations because besides being those actually performed to obtain the nonadiabatic absorption spectrum, they have the advantage to provide a clear picture of internal conversion between states with a well-defined electronic character. However, this analysis is complemented below, with the results for photoexcitation to states corresponding to the adiabatic bright states in the FC position. The results of the DM structure reveal that when the two interacting monomers are well-separated (*i.e.*, at a long distance), 50% of the population decays to other LE states within 10 fs following initial photoexcitation from each of |*L*_1_⟩ and |*L*_1_^′^⟩ diabatic states. Then, population exchanges between different LE states give rise to relatively large population oscillations over time, associated with sizable LE–LE couplings that still exist in DM although the monomers are well-separated (large *d*_P–P_ and *d*_Zn–Zn_ distances) and are very effective since the LE states are practically degenerate. Noteworthy, at such a long distance, the CT states do not gain population over time, as expected from the computed energy gaps and LE–CT couplings (see Table S8 in the Supporting Information), and by the fact that the minima of the CT states remain remarkably less stable than the minima with LE character (see Table S11 in the Supporting Information).

In the OM structure, with closely interacting monomers, CT states are stabilized and CT–LE couplings are turned on making the population dynamics more complex. More than 75% of the |*L*_1_⟩ state population decays within 7 fs to other LE states and the CT states start to get already smoothly populated, as a result of sizeable LE–CT couplings. This is followed by relatively large population oscillations up to 20 fs, and then the wave packet flows predominantly to the CT states. Indeed, after 80 fs, one can see that most of the excited population (>90%) is distributed over the CT states. This result is in line with the fact that CT minima are more stable than LE ones (see Table S11 in the Supporting Information).

Focusing on the representative Zn Pc dimers taken from MD clustering, the differences in the QD plots obtained for each CS and the comparison with the results obtained for the dimers at the static approximation highlight the sensitivity of the time-evolving populations to the fluctuation of the soft coordinates and the structural dynamics of the **Zn PcF12-SH** dimer. For the CS3 and CS4 structures, an ultrafast population transfer occurs within ∼7 fs, with more than 60% of the |*L*_1_⟩ population decaying mostly to the |*L*_2_⟩ state and to a less extent to the other LE states, as a result of strong |*L*_1_⟩–|*L*_2_⟩ coupling (see Figure S23 in the Supporting Information). This population transfer is followed by relatively large quantum beats and |*L*_1_⟩–|*L*_2_⟩ population exchanges, more evident in the first 40 fs due to the coupling with the vibrational motions, while other LE state populations show weaker oscillations. Interestingly, all the MD Zn Pc dimers exhibit a relatively smooth and steady growth in population of the CT states over time but, here, the various Zn Pc structures differ significantly in quantitative terms from each other. In fact, while for CS3 and CS4, a considerable population is still carried by the LE states after 100 fs, CS1 shows more efficient LE-to-CT population transfers (namely, transfers to |CT(1 → 2)⟩ and |CT(2 → 1)⟩ from |*L*_1_⟩ and |*L*_2_⟩, respectively) but, here, |*L*_2_⟩ still holds a significant population up to 60 fs when it finally decays to the |CT(2 → 1)⟩ state. As expected from its peculiar geometric conformation and large LE–CT couplings discussed above, the electronic populations for CS2 feature a remarkably different time evolution with respect to the other structures, with faster and more effective transfers to both LE and CT states from the initially populated |*L*_1_⟩ (or |*L*_1_^′^⟩, see Figure S30 in the Supporting Information). Indeed, ∼90% of the initial population is transferred within 7 fs, followed by relatively large and fast oscillations, with almost all the population distributed over just CT states within 50 fs. The rapid transfer to CT states of CS2 resembles that of OM; however, the population distribution of specific CT states in the two cases is rather different. Particularly, only one specific CT state, *i.e.,* |CT(2 → 1)⟩, is predominantly populated in the CS2 state, reaching ∼50% of the population after 100 fs. In OM, instead, the final population of CT states is more equally distributed. This is explained by the large stabilization of the |CT(2 → 1)⟩ state compared to that of other CT states in CS2 (see Figure 7a and Table S11 in the Supporting Information), which is due to the specific geometrical distortion in solution, and the extent of the corresponding LE–CT coupling (see Figure S25 in the Supporting Information). Thus, from the above analysis, we could conclude that a significant part of the photoexcited population flows to states with CT character on an ultrafast timescale. This is simply explained with the fact that these states have the largest reorganization energy and therefore, though being higher in energy than LE ones in the FC region, they have the most stable minima (see Table S11 in the Supporting Information).

**Figure 10:**
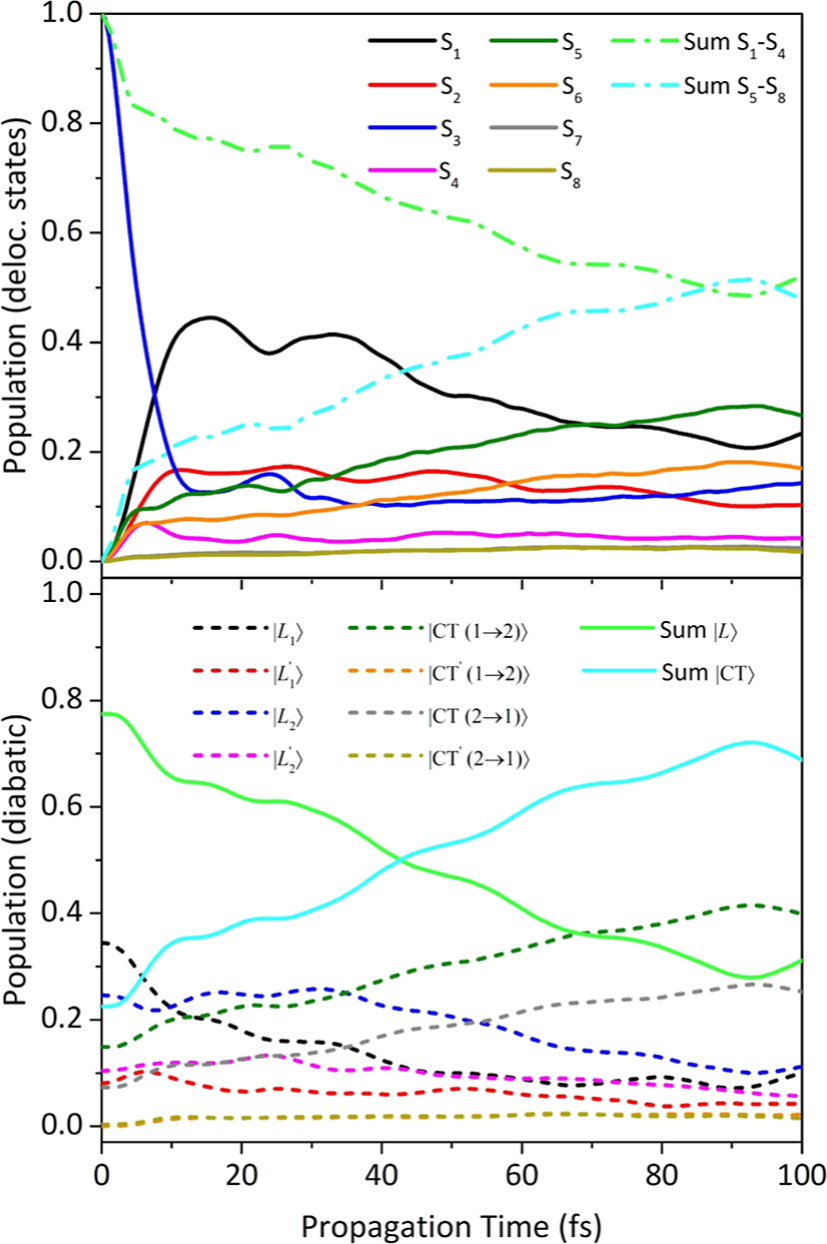
Population dynamics of the delocalized states (top) and diabatic states (bottom) of the CS1 Zn Pc dimer after photoexcitation to the combination of diabatic states corresponding to the lowest energy bright adiabatic state S_3_ in the FC point. ML-MCTDH propagations including 30 effective coordinates.

In order to further validate our conclusions (based on QD upon photoexcitation of a LE state), we performed QD simulations by photoexciting the combinations of the diabatic states that correspond to the two lowest bright adiabatic states in the FC position. It is noteworthy that, formally speaking, the new states we excited are still “diabatic” since they are combinations of diabatic states with fixed coefficients, whereas true adiabatic states are obtained *via* the diagonalization of the LVC Hamiltonian as a function of the nuclear coordinates. Here, we focused on the QD for the CS1 configuration after an excitation to the lowest bright state S_3_, as exemplifying case. Figure 10 reports the time-evolving populations of the delocalized states corresponding to the adiabatic states at the FC position (S_1_–S_8_), following initial excitation to S_3_. By analyzing the time-evolving wave packet in terms of population of the localized LE and CT diabatic states, the results clearly confirm that even starting from such a bright delocalized exciton, a remarkable amount of population flows into CT states (the sum of CT populations is 70% after 100 fs). On the contrary, the analysis of the time-evolving wave packet in terms of the population of the delocalized states is more complicated because, as a matter of fact, all of them include both LE and CT characters. However, the bottom panel of Figure 10 shows that the sum of the populations of S_1_ and S_2_, mostly corresponding to the two quasi-dark excitons often considered responsible of the quenching of the emission, does not exceed 0.35 in 100 fs.

QD initiated exciting other bright states and/or starting from different dimer structures have been analyzed and reported in Figures S31–S33 in the Supporting Information, all providing similar results, except for DM, where CT states are less stable and do not interact efficiently with LE ones. A further converging information was obtained from a QD started on S_1_, corresponding to the lowest adiabatic state at the FC position, *i.e.,* the quasi-dark exciton mostly arising from the antisymmetric combination of the two |*L*_1_⟩ states. Our results, reported in Figure S31 in the Supporting Information, showed that even populating this state (although clearly it can be challenging to excite it with a laser pulse), most of the population still flows in very short time in CT states.

In summary, the QD results presented here suggest that population of CT states can provide an alternative channel to explain the non-emissive nature of the Zn Pc aggregates. In fact, although LVC models cannot predict the fate of CT populations at later times, it is reasonable to guess that solvent equilibration will remarkably stabilize states with CT character trapping their population.

#### 4.3.1 Correlation between Inter-molecular Stacking Distances and the Population of CT States

**Figure 11:**
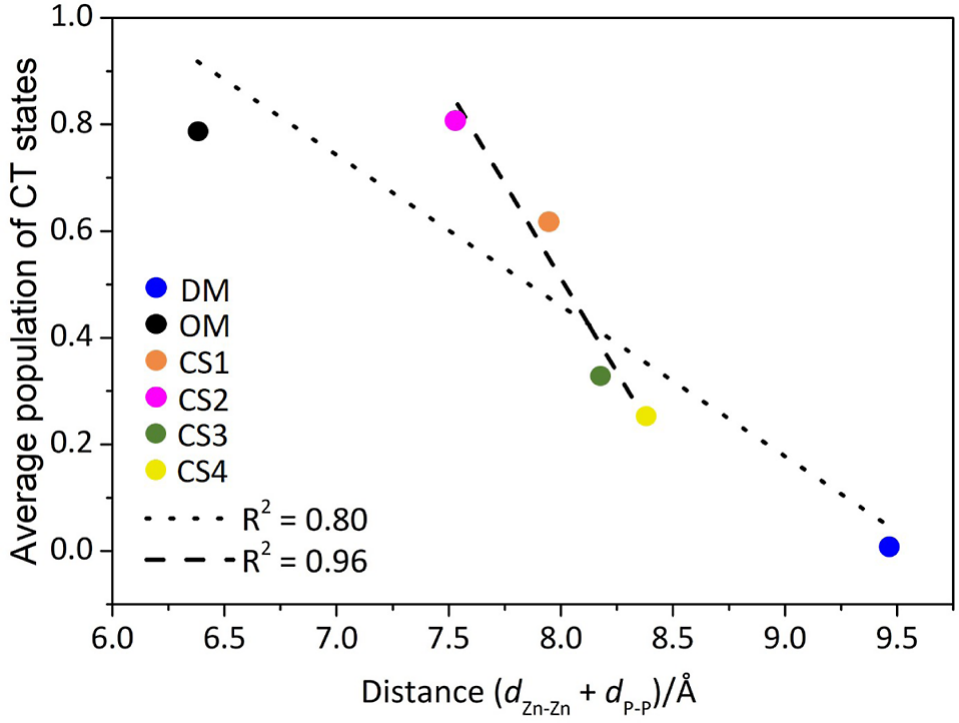
Dependence of the total population of the CT states after 100 fs, averaged over initial photoexcitation from the |*L*_1_⟩ and |*L*_1_^′^⟩ states, on the summation of *d*_Zn–Zn_ and *d*_P–P_ stacking distances. The dashed and dotted lines, respectively, represent the linear correlations excluding and including the OM and DM dimers.

The analysis of the time-evolution of the populations provided thus two relevant results. On one side, the CSs of the two most populated clusters in the MD simulation in water solution, *i.e.,* CS1 and CS2, feature the largest populations of non-emissive CT states after 100 fs after photoexcitation, in line with the experimental observation for **Zn PcF12-SH** in aqueous solution. On the other hand, the significant amount of population transfer to CT states is unexpected for the CS1 structure, if one considers the corresponding simulated absorption spectra (quite similar between CS1 and CS3/CS4 and different from CS2) and their interpretation based on the geometrical parameters discussed above (*i.e.*, *d*_P–P_ distance of CS1 larger than that of CS2 and closer to those of CS3/CS4). Interestingly, these results indicate that the argumentation for the geometrical effects on the absorption properties of the Zn Pc dimers cannot be straightforwardly used to explain the emission quenching occurring *via* CT population. Thus, looking for the geometrical parameters that determine the population of CT states, we evaluated the potential correlation between the total population gained by the CT states after 100 fs of wavepacket dynamics (of both |*L*_1_⟩ and |*L*_1_^′^⟩ states) and either the *d*_Zn–Zn_ or the *d*_P–P_ geometrical parameters. Considering just the CS1–4 structures of the Zn Pc dimer in water solution, we observed that while a good degree of correlation is found between CT states populations and the *d*_Zn–Zn_ distance (with *R*^2^ = 0.85), this is not the case for the *d*_P–P_ distance (with *R*^2^ = 0.38), see Figure S34 in the Supporting Information. However, as shown in Figure 11, when considering as geometrical parameter the sum of these two distances (*i.e.,d*_Zn–Zn_ + *d*_P–P_), the linear correlation increases significantly, with *R*^2^ = 0.96. These results indicate that the interplane *d*_P–P_ distance plays a less relevant but not negligible role in the population of dark CT states, with respect to the global inter-monomer distance, represented by the *d*_Zn–Zn_ parameter. If the reduced OM and DM models are considered, *i.e.,* looking at the “extreme” values of *d*_Zn–Zn_ and *d*_P–P_ parameters, we observed that the maximum CT population (*ca.* 80% of the initial population in LE states, *i.e.,* |*L*_1_⟩ and |*L*_1_^′^⟩) is reached for the closely bound OM dimer, while the minimal LE-to-CT transfer (*i.e.* 0%) occurs for the very distant monomers in the DM model, as expected. Notably, the CS structure with the smallest *d*_Zn–Zn_ and *d*_P–P_ distances (and the smallest sum *d*_Zn–Zn_ + *d*_P–P_), *i.e.,* CS2, features a CT population equal to OM, indicating that the LE-to-CT maximum transfer can be indeed obtained in the real Zn Pc dimers in water solution. Due to the presence of such plateau of CT population below the *d*_Zn–Zn_ + *d*_P–P_ value of CS2, the linear correlation slightly worsens (with *R*^2^ = 0.80) when including both OM and DM models.

In summary, the results of our QD simulations of electronic populations showed that the two most representative structures of stacked **Zn PcF12-SH** monomers in water solution, *i.e.,* CS1 and CS2, feature the largest populations of dark CT states, suggesting that emission quenching can occur *via* CT population. The efficiency of this LE-to-CT transfer is similar in these two structures and larger than that in CS3–4, because they feature similar global distance between monomers (*d*_Zn–Zn_), despite having significantly different interplane (*d*_P–P_) distances. This indicates that the population transfer to CT could efficiently occur even if the interplane distance is not very short, thus is plausible for many **Zn PcF12-SH** dimeric structures thermally available in aqueous solution. The results, thus, suggest a mechanism of **Zn PcF12-SH** fluorescence quenching in water solution associated with efficient population of non-emissive CT states, which is complementary to those typically associated with H-type Zn Pc aggregates in solution, invoking for population of excitons associated with the coupling of LE states and/or non-radiative energy relaxation pathways due to exciton–exciton annihilation phenomena.^12,15,28,87^

## 5 Conclusions

A zinc phthalocyanine (*i.e.*, **Zn PcF12-SH**), designed for potential applications in hybrid organic–inorganic photosystems, has been used as a target chromophoric system for the development of a quantum-classical protocol for simulations of absorption spectra and ultrafast nonadiabatic dynamics in large molecular systems undergoing aggregation in solution. This approach describes with nonadiabatic vibronic QD the effect of the coupling of LE and CT states and their competition with intra-monomeric internal conversions, like those triggered by JT couplings, and it accounts also for the effect of the fluctuations of the aggregates and the surrounding solvent. We experimentally recorded absorption and emission spectra in pure DMSO and aqueous solution to be used as reference to validate the theoretical approach, which represents a computationally very convenient variant of the adiabatic MD with a generalized linear vibronic coupling (Ad-MD|gLVC) model. The initial benchmark of various adiabatic and nonadiabatic vibronic approaches for simulating the absorption spectrum of monomeric Zn Pc species showed the reliability of the linear vibronic coupling model in reproducing the lineshape of the experimental spectrum in DMSO and, more importantly, indicated that the JT coupling effect is not large enough to substantially alter the spectral lineshape in the Zn Pc compound considered. **Zn PcF12-SH** aggregation in water solution induces a significant modification of the absorption spectrum lineshape, with the Q-band peak height intensity dropping and significantly broadening, resulting in a vibronic progression with two peaks at comparable intensities. To characterize these spectral modifications and to provide computationally affordable models for the study of aggregates, we first considered reduced models, *i.e.,* truncating the side chains and using effective normal modes for the dynamics, for the monomeric and the dimeric Zn Pc species. The monomeric reduced models were used to evaluate the effect of regioisomers on the absorption properties of each Zn Pc unit, which were found to be of small extent, and to reduce the computational cost for simulations of dimers, by employing approximations involving computations of vibrational frequencies and diabatization procedures. Two Zn Pc dimer structures were constructed using the monomeric reduced models, representing two extremes of inter-monomers interactions, *i.e.,* non-interacting (distant) and very closely interacting (optimized) Zn Pc units, namely, DM and OM models, respectively. Comparing the TD-DFT, adiabatic, and nonadiabatic vibronic spectra of the OM model indicated that inter-state couplings, explicitly characterized by the diabatization procedure, significantly contribute to the spectral broadening associated with Zn Pc aggregation. Decomposing the effect of each type (local or charge transfer, *i.e.*, LE or CT) of the electronic state and of their couplings (LE–LE, LE–CT, and CT–CT) revealed that the absorption peak blue shifts and the lineshape broadening associated with **Zn PcF12-SH** aggregation are mainly driven by exciton interactions among LE states, as expected in H-type aggregates. Moreover, the modulation of the relative intensities of the vibronic bands is due to the LE–CT couplings. This outcome showed how the roles of specific inter-state couplings are different in Zn Pc aggregates in solution with respect to what occurs in symmetric Zn Pc thin films, as investigated in refs (32)–^34^. Moving toward the application of our methodology to realistic models of Zn Pc dimer formation in water solution, we coupled our protocol to MD simulations and cluster analysis in order to include the static-disorder effect of the fluctuations of the soft degrees of freedom of the system associated with the conformational dynamics of the solvated **Zn PcF12-SH** dimer. The nonadiabatic vibronic spectra of four representative conformations of Zn Pc dimers have been compared with those of the reduced (OM and DM) models, demonstrating a modulation of the spectral lineshape as a function of the thermal dynamics in solution. Moreover, the analysis of the structure-dependent absorption properties allowed characterization of the interplay among high-frequency molecular vibrations, electronic states coupling, and thermal dynamical effects. We found that when considering specific (while representative) conformations, vibronic effects are crucial to reproduce the experimental spectral shapes, whereas phenomenological broadenings applied to vertical excitations from TD-DFT computations provide simulated absorption spectra that are inconsistent with the spectral lineshape of aggregated forms. Moreover, our approach allowed disentanglement of various contributions determining the overall lineshape of aggregated **Zn PcF12-SH**. In particular, we found that the couplings between LE and CT states, strictly correlated with the energy gaps between these states, are the most sensitive to geometrical deformation of the **Zn PcF12-SH** aggregates. These LE–CT energy gaps were in turn found to be linearly correlated with the distance between macrocycle planes of two Zn Pc monomers, resulting to be the geometrical parameter mainly causing the large mixing and electronic couplings between LE and CT states. While the interplane distance resulted to be the primary contribution to the absorption spectrum changes upon aggregation, a significant contribution was observed for conformations featuring tilting of the macrocycle planes, with non-negligible orbital overlaps between two monomers affecting the overall broadening of the absorption spectrum. Focusing on the experimental evidence of fluorescence loss in **Zn PcF12-SH** aggregates in aqueous solution, we analyzed the electronic-state populations associated with wavepacket QD of all dimer models considered in this work. We found that population transfer to non-emissive CT states is plausible for Zn Pc dimeric structures thermally available in water solution, suggesting that fluorescence quenching in solution can likely occur *via* such a mechanism. In contrast to absorption properties, we found that the CT population transfer efficiency is linearly correlated more with the distance between Zn centers than with the interplane distance, suggesting that fluorescence quenching in solution can efficiently occur even when the interplane distance between Zn Pc units is not very short (*i.e.*, above 3.7 Å). Thus, our results offer an explanation for the non-emissive character of the H-type stacked Zn Pc forms in solution, complementary to the population of dark exciton states or to exciton–exciton annihilation effects, demonstrating that a significant part of the excited populations can actually flow to almost dark CT states. In conclusion, the development of an efficient quantum-classical protocol reveals several insights for the understanding of the spectroscopic properties, such as absorption spectra lineshape and fluorescence quenching, of **Zn PcF12-SH** aggregates in solution at the atomistic level, paving the way for other relevant applications on molecular photosystems that feature aggregation-dependent optical properties in solution and potentially relevant Jahn–Teller (JT) vibronic coupling effects.

## Supporting Information Available

Further details on the theory and computation, including St-LVC and FrD diabatizations, JT effect, computation of nonadiabatic spectra, definition of diabatic states, ML-MCTDH settings, and convergence tests; detailed discussion on the excited-state properties and vibronic absorption spectra of various regioisomers of the Zn Pc monomer, further details on the experiment including synthesis procedure, fluorescence and excitation spectra of **Zn PcF12-SH** in DMSO as well as its absorption spectra in both pure DMSO and aqueous solution in full frequency range; and additional theoretical results on the monomer and dimer models (PDF)
