# Supplementary material for: Quantum-Classical Protocol for Efficient Characterization of Absorption Lineshape and Fluorescence Quenching upon Aggregation: The Case of Zinc Phthalocyanine Dyes
Source: J Chem Theory Comput. 2023 Aug 29;19(17):5938–57. doi: 10.1021/acs.jctc.3c00446 (PMC10500990; doi:10.1021/acs.jctc.3c00446)
Supplement: Supplementary file 1 — ct3c00446_si_001.pdf [file ct3c00446_si_001.pdf]

Supporting Information:

A Quantum-Classical Protocol For Efficient Characterization of Absorption Lineshape and Fluorescence Quenching Upon Aggregation: The Case of Zinc Phthalocyanine Dyes

Mohammad Aarabi,[†] Daniel Aranda,[‡,¶] Samira Gholami,[†] Santosh Kumar Meena,[§] Frederic Lerouge,[‖] Yann Bretonniere,[‖] Ilke Gürol,[⊥] Patrice Baldeck,[‖] Stephane Parola,[‖] Fabienne Dumoulin,[#] Javier Cerezo,[@,‡] Marco Garavelli,[†] Fabrizio Santoro,[*,‡] and Ivan Rivalta[*,†,‖]

†*Dipartimento di Chimica Industriale "Toso Montanari", Universitá degli Studi di Bologna, Viale del Risorgimento 4, I-40136 Bologna, Italy*

‡*Consiglio Nazionale delle Ricerche, Istituto di Chimica dei Composti Organo Metallici (ICCOM-CNR), I-56124 Pisa, Italy*

¶*Instituto de Ciencia Molecular (ICMol), Universidad de Valencia, Catedrático J. Beltrán 2, 46980, Paterna, Valencia, Spain*

§*Department of Chemical Engineering, Indian Institute of Technology Ropar, Rupnagar 140001, Punjab, India*

‖*ENSL, CNRS, Laboratoire de Chimie UMR 5182, 46 Allée d'Italie, 69364 Lyon France*

⊥*TÜBITAK Marmara Research Center, Materials Technologies, Gebze, Kocaeli, 41470 Türkiye*

#*Department of Biomedical Engineering, Faculty of Engineering and Natural Sciences, Acibadem Mehmet Ali Aydinlar University, Istanbul 34752, Türkiye*

@*Departamento de Química and Institute for Advanced Research in Chemical Sciences (IAdChem), Universidad Autónoma de Madrid, 28049 Madrid, Spain*

E-mail: fabrizio.santoro@pi.iccom.cnr.it; i.rivalta@unibo.it

Contents

1 Additional Computational Details S-3

1.1 The St-LVC Model and Jahn-Teller Effect S-3

1.2 Adiabatic-to-Diabatic Transformation for Dimers S-4

1.3 Computation of the Spectra S-4

1.4 Diabatic States S-6

1.5 ML-MCTDH Settings S-7

1.6 Convergence Tests S-9

1.7 Molecular Dynamics Simulations S-12

2 Synthesis of Zn PcF12-SH S-13

3 Absorption Spectroscopy of Different Regioisomers of Zn Pc Monomer S-16

3.1 Fundamental Absorption Properties S-16

3.2 Adiabatic Vibronic Spectra S-19

4 Simulated Absorption Spectrum of a Zn Pc Trimeric Species S-30

5 Definition of Intermolecular Coordinates For Zn Pc Dimers S-33

References S-52

1 Additional Computational Details

1.1 The St-LVC Model and Jahn-Teller Effect

In a tetragonal D_{4h} system with a pair of degenerate states of the E_g or E_u symmetry, the $E \otimes b$ JT Hamiltonian for a single pair of symmetry breaking coordinates is defined as

$$\mathbf{H_{JT}} = H_0 \mathbf{1} + \begin{pmatrix} k_1 Q_1 & k_2 Q_2 \\ k_2 Q_2 & -k_1 Q_1 \end{pmatrix} \tag{1}$$

where H_0 is the harmonic oscillator for non JT active coordinates and k_1 and k_2 are gradient and coupling parameters, respectively. Q_1 and Q_2 are modes that carry all gradient or coupling, respectively. Thus, one may find k_1Q_1 and k_2Q_2 expressions identical to λ_{ii}^Tq and λ_{ij}^Tq in the LVC Hamiltonian for each pair of symmetry-breaking modes. In a D_{4h} system, these Q_1 and Q_2 modes respectively correspond to the symmetry-breaking b_{1g} and b_{2g} coordinates that operate JT distortions for E_g electronic states and to b_{1u} and b_{2u} coordinates for E_u. In the particular case of Zn Pc, the symmetry of the bright pair of electronic states is E_u. In the lowest order approach, the JT Hamiltonian is linear on the symmetry breaking coordinates and this suggest that we can safely use our LVC Hamiltonian to properly introduce the JT effect on the spectra, as it shows linear dependency on $\mathbf{q}$ as well.

To obtain the linear coupling k_1 and k_2, identified as λ_{ij} in the notation of the main text, we used the procedure detailed in Ref. S1. First, we displace the geometry of truncated model of **Zn PcF12-SH** monomer along each dimensionless normal coordinate $\mathbf{q}_\alpha$ by a small amount $\Delta_\alpha = \pm 0.02$. Then, for each displaced geometry we compute the adiabatic states $|\mathbf{a}(\Delta_\alpha)\rangle$ and the matrix elements of their overlap with the reference states at equilibrium geometry, i.e. $S_{ij}(\Delta_\alpha){=}\langle R_i(0)\,|a_j(\Delta_\alpha)\rangle$. At each step, this provides us with a new transformation matrix $\mathbf{D}(\pm\Delta_\alpha)$, which can be applied to the diagonal matrix of adiabatic energies of the Zn Pc monomer to obtain the diabatic matrix of the potential terms at

displaced geometry

$$\mathbf{V}^d(\Delta_\alpha) = \mathbf{D}^T(\Delta_\alpha)\mathbf{V}^{ad}(\Delta_\alpha)\mathbf{D}(\Delta_\alpha) \tag{2}$$

Once we get the off-diagonal terms $V_{ij}^d(\mathbf{q}_\alpha)$, we can apply them to perform a numerical differentiation to compute the linear coupling terms λ_{ij}

$$\lambda_{ij}(\alpha) = \frac{\partial V_{ij}^d(\mathbf{q}_\alpha)}{\partial q_\alpha} \simeq \frac{V_{ij}^d(\Delta_\alpha) - V_{ij}^d(-\Delta_\alpha)}{2\Delta_\alpha} \tag{3}$$

1.2 Adiabatic-to-Diabatic Transformation for Dimers

In the followed approach, we define diabatic states of truncated **Zn PcF12-SH** dimer on the basis of reference states $|R^{Frags}\rangle$ of either the adiabatic states of the isolated fragments/monomers of the dimer (for LEs) or one electron transitions between orbitals on different fragments (for CT states).[S1,S2] The diabatic states are then obtained through the following adiabatic-to-diabatic transformation

$$|\mathbf{d}\rangle = |\mathbf{a}^{Dimer}\rangle\,\mathbf{D} = |\mathbf{a}^{Dimer}\rangle\,\mathbf{S}^T(\mathbf{S}\mathbf{S}^T)^{-1/2} \tag{4}$$

where $\mathbf{S} = \langle\mathbf{R}^{Frag}\,|\mathbf{a}^{Dimer}\rangle$ is the overlap of the reference states of the fragments with the adiabatic states of the Zn Pc dimer. Performing the latter operation at the reference geometry yields the transformation matrix $\mathbf{D}(\mathbf{0})$. This can be applied to the diagonal matrix containing the energies of the adiabatic states of the Zn Pc dimer to obtain the diabatic matrix, which contains the diabatic energies $E_{ii}^{\mathrm{d}}(0)$ on diagonal and inter-state couplings $E_{ij}^{\mathrm{d}}(0)$ on off-diagonal.

1.3 Computation of the Spectra

The complete expression for the absorption spectrum $\epsilon(\omega)$ at zero Kelvin is given by:

$$\begin{aligned}\epsilon(\omega) &= \frac{2\pi\omega N_A}{3000 \times \ln 10 \times \hbar c_0 (4\pi\epsilon_0)} \sum_{ji} \int_{-\infty}^{\infty} dt e^{i(E_{g0}+\hbar\omega)/\hbar t - \Gamma t^2} \langle \mathbf{0}; d_j | \boldsymbol{\mu}_{gj} e^{-iHt/\hbar} \boldsymbol{\mu}_{ig} | d_i; \mathbf{0} \rangle \\ &= \sum_{ii} \epsilon_{ii}(\omega) + \sum_{ij, j\neq i} \epsilon_{ij}(\omega) = \epsilon^{auto}(\omega) + \epsilon^{cross}(\omega) \end{aligned} \tag{5}$$

and depends on a set of physical constants (Avogadro's number N_A, Plank's constant $\hbar$, the speed of light c_0 and vacuum permitivity ϵ_0). The ground state energy E_{g0}, the quadratic damping term $e^{-\Gamma t^2}$ and the electric transition dipole moment $\boldsymbol{\mu}_{ig}$ were already discussed in the main text.

The correlation functions are obtained from the following expression:

$$\phi_{ij}(t) = \langle d_i(0) | d_j(t) \rangle \tag{6}$$

where $|d_i(0)\rangle$ is the initial wavefunction of the state i and $|d_j(t)\rangle$ is the time-evolved wavefunction of state j. Notice that the auto terms are obtained when $j = i$. Once all the correlation functions are collected, we calculate the "total" correlation function of the systems as the sum of all the correlation functions, each of them weighted by the scalar product of the transition dipoles the corresponding states, i.e.:

$$\phi_{tot}(t) = \sum_{i, j\neq i} \boldsymbol{\mu_{gi}} \cdot \boldsymbol{\mu_{jg}} \; \phi_{ij}(t) \tag{7}$$

Finally, the total spectrum is obtained by Fourier transform of the total correlation function ϕ_{tot}.

1.4 Diabatic States

The diabatic states for the truncated **Zn PcF12-SH** dimers considered in this study were defined according to the scheme shown in Figure S1.

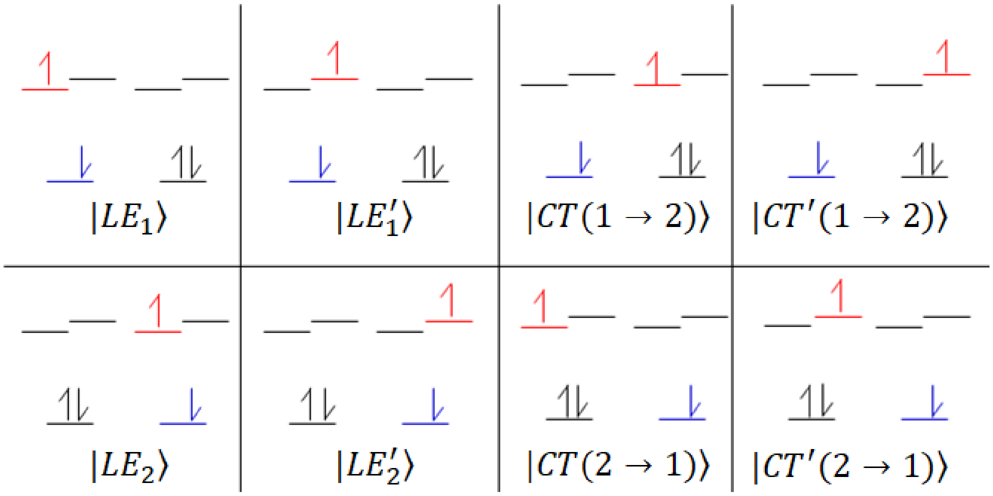

Figure S1: Sketch of the electronic excitations on the truncated **Zn PcF12-SH** dimer. The orbital from which the electron is coming from (and the hole is forming) upon excitation is shown in blue, while the orbital to where the electron arrives is highlighted in red. The states where the final orbital is LUMO+1 are labeled with an apostrophe. The diabatic states $|L_1\rangle$, $|L_1'\rangle$, $|L_2\rangle$ and $|L_2'\rangle$ used on the LVC Hamiltonian are linear combinations of the local excitations $|LE_1\rangle$, $|LE_1'\rangle$, $|LE_2\rangle$ and $|LE_2'\rangle$ as defined by the TD-DFT vectors.

1.5 ML-MCTDH Settings

For the monomer of truncated **Zn PcF12-SH** with 213 fast coordinates, a straightforward application of MCTDH method, including only 2 blocks with 6 coordinates (Q, 3 per block) provided us with the converged spectrum for a Gaussian function of the half width at half-maximum (HWHM) of 0.04 eV (see next section for convergence tests). The first block includes three coordinates, of which two of them respectively carry all the gradients for states $|L\rangle$ and $|L'\rangle$, and the third contains the linear couplings due to JT effects. For dimer instead, with a total number of 426 normal modes and inclusion of the JT-associated λ_{ij} couplings coming from St-LVC diabatization on the monomer, we adopted its ML extension, i.e. ML-MCTDH, which allows for inclusion of more coordinates and ensures getting fully-converged spectra in a fast and effective way. Accordingly, we adopted 3 blocks of 10Q with a effective collection of 30Q, provided with a hierarchical representation of the LVC Hamiltonian based on HEMS, to compute the spectra with a HWHM of 0.04 eV for the dimer structures taken from MD clustering and quantum mechanical optimization. The first block contains 10Q, each of them containing all gradients the gradient for the 8 diabatic states $|L_1\rangle$, $|L'_1\rangle$, $|L_2\rangle$, $|L'_2\rangle$, $CT(1 \to 2)$, $CT'(1 \to 2)$, $CT(2 \to 1)$ and $CT'(2 \to 1)$, respectively and two additional coordinates for the inter-state JT coupling of states $|L_1\rangle$ with $|L'_1\rangle$ and $|L_2\rangle$ with $|L'_2\rangle$. Mode combination was also utilized for modes with a similar character. Under this setup, the non-adiabatic QD propagations of the vibronic wave packets on the coupled PESs were performed, adopting the MCTDH and ML-MCTDH for the truncated monomer and dimer models of **Zn PcF12-SH**, respectively, to compute time-dependent correlation functions in a short dynamics up to 100 fs. This time scale allows to capture and introduce the most important non-adiabatic features on the spectra in a fully-converged low-resolution way. The graphical representation of the MCTDH and ML-MCTDH trees are shown in Figures S2 and S3, respectively.

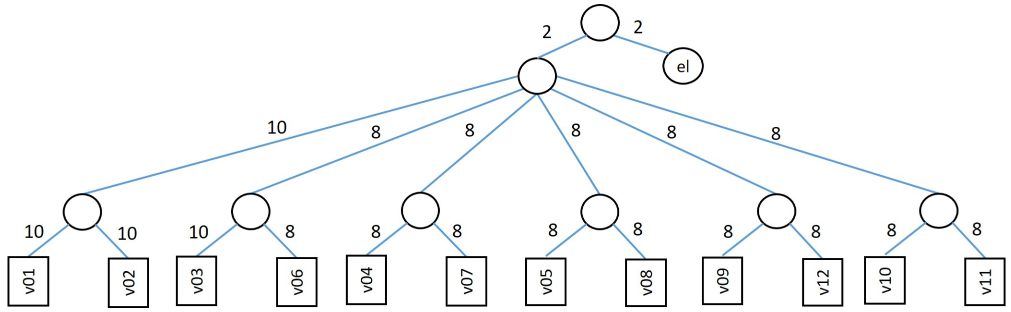

Figure S2: Graphical representation of the MCTDH tree adopted in the QD computations of the truncated **Zn PcF12-SH** monomer (regioisomer C) for a set of 12 normal coordinates. The numbers close to the lines indicate the single particle functions used to combine or the number of primitive functions to describe each normal coordinate.

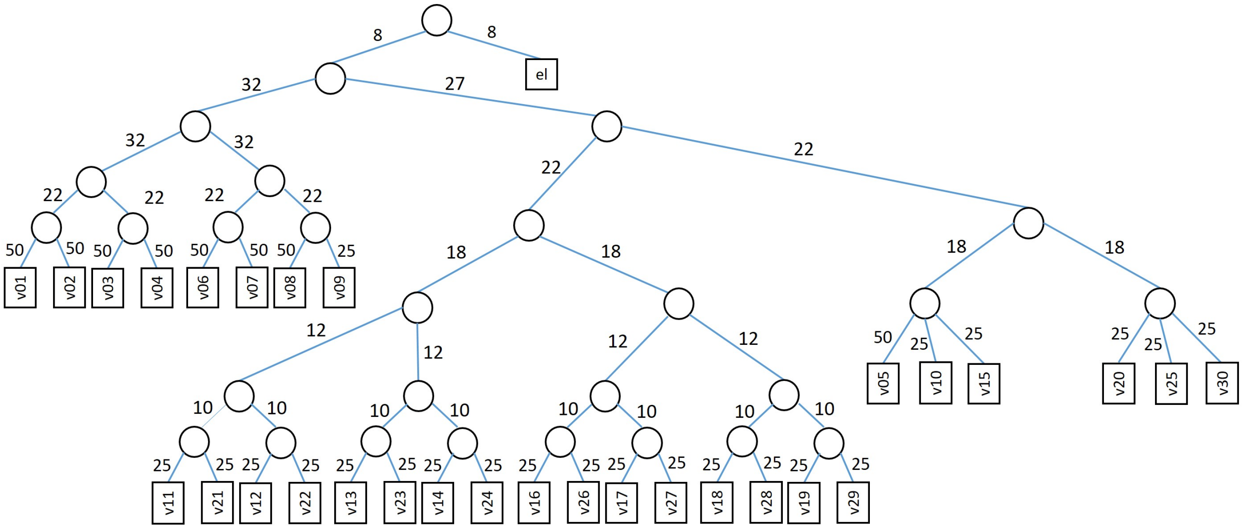

Figure S3: Graphical representation of the ML-MCTDH tree adopted in the QD computations of the truncated **Zn PcF12-SH** dimers for a set of 30 normal coordinates. The numbers close to the lines indicate the single particle functions used to combine each mode or the number of primitive functions to describe each normal coordinate.

1.6 Convergence Tests

Figure S4 shows that the LVC spectrum of the monomer can be considered as converged with 2 blocks (6Q), since increasing to 3 (9Q) or even 4 blocks (12Q) results on very minor changes on the spectral shape.

For the dimer, initially we have 10 collective coordinates per block (8 gradients and the linear coupling between the two local excitations of each monomer). In Figure S5 we compared the LVC spectra obtained for OM dimer model with 2 blocks (20Q), 3 blocks (30Q) and 4 blocks (40Q). The spectra are similar, indicating only minor changes in the low-energy band. Therefore, we considered that adopting 3 hierarchy blocks with a effective collection of 30Q is sufficient to reproduce the optical properties of the Zn Pc dimers with this resolution.

Figure S6 compares the spectra obtained for the OM dimer model of **Zn PcF12-SH** where only the four LE states were included, using the gradients from the monomer bright state computed at the TD-DFT level or those obtained from the LVC by displacing along each normal coordinate. We employed 3 blocks of 6Q (4 gradients and the linear coupling between the two local excitations of each monomer) with a effective collection of 18Q. Both provide similar spectral shapes but with some differences on the total width and on the intensity of the maximum. To be consistent with our calculations on the monomer, we used the LVC gradients for the rest of dimer calculations.

Table S1: Norm of the coupling vectors, in eV, of the Zn Pc monomer (regioisomer C) diabatic states. Parametrization at CAM-B3LYP/6-31G(d,p)/PCM(DMSO) level of theory. Notice that the matrix is symmetric and only the upper half is reported. The term on the ij, $j \geq i$ position represents $\sum_{\alpha} \lambda_{ij}(\alpha)$, therefore, the terms in the diagonal are the excited state gradients and the off-diagonal term is the excited state coupling.

State	1	2
1	0.16	0.09
2		0.17

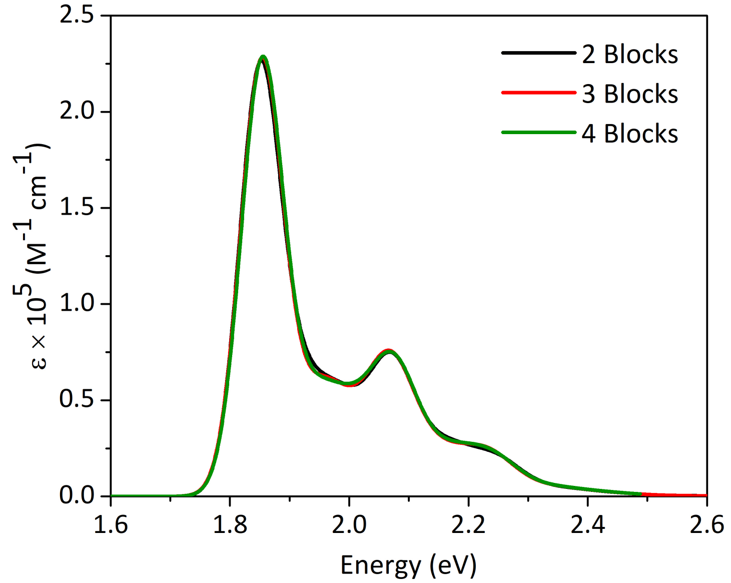

Figure S4: Convergence test for the number of hierarchical blocks for the Zn Pc monomer (regioisomer C). Each block contains 3 collective coordinates, employing MCTDH. All stick transitions were convoluted with a Gaussian of HWHM = 0.04 eV.

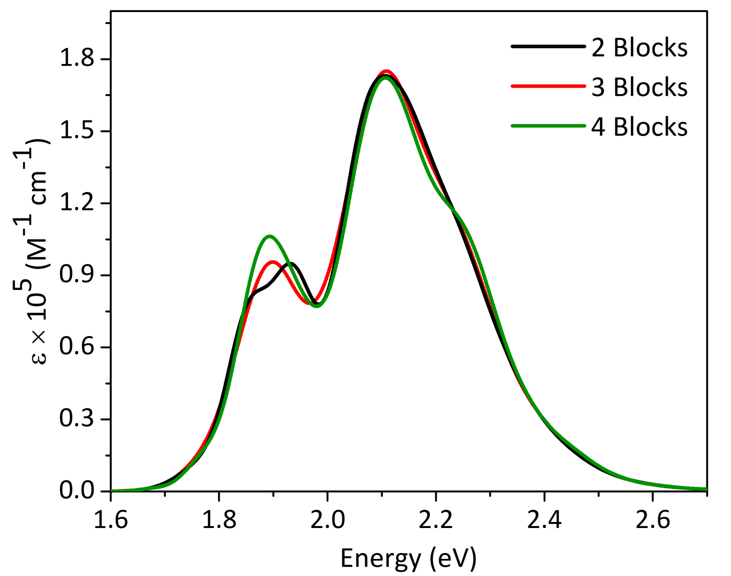

Figure S5: Convergence test for the number of hierarchical blocks for the OM dimer model. The sets with 2, 3 and 4 blocks contain 20, 30 and 40 coordinates, respectively, employing ML-MCTDH. All stick transitions were convoluted with a Gaussian of HWHM = 0.04 eV.

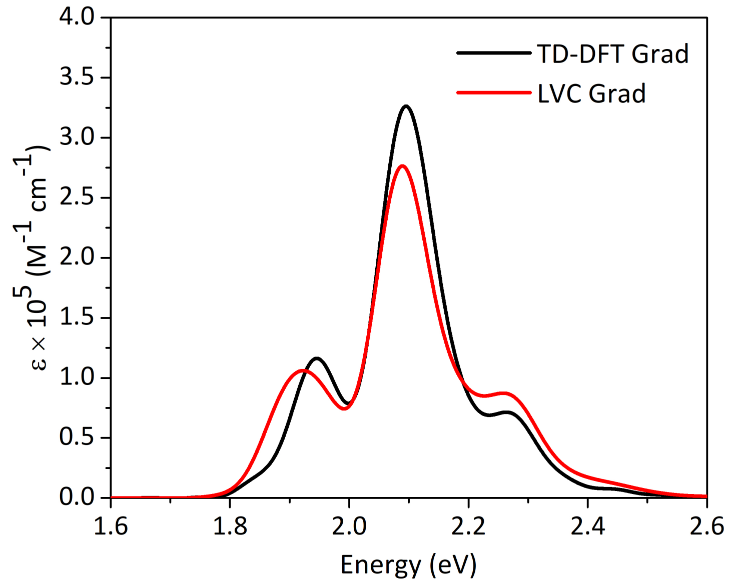

Figure S6: LVC spectrum for the OM dimer model of **Zn PcF12-SH** using a reduced model with all local excitations only (four LE states). Two sets of monomer gradients were taken, employing ML-MCTDH with 18Q (3 blocks of 6Q): those calculated directly from TD-DFT (black line), or the LVC gradients obtained by displacing along each normal coordinate (red line). All stick transitions were convoluted with a Gaussian of HWHM = 0.04 eV.

1.7 Molecular Dynamics Simulations

Table S2 collects details of the model systems used for the MD simulations of full structures of **Zn PcF12-SH** molecules in water and DMSO solvents.

Table S2: Model details for **Zn PcF12-SH** molecules in aqueous and DMSO solutions.

Name of the system	No. of Zn Pc molecules	No. of solvent molecules	Box dimensions X [nm], Y [nm] Z [nm]
Zn Pcs in water	2	4046	5.02, 5.02, 5.02
Zn Pcs in DMSO	2	998	4.75, 4.75, 4.75

2 Synthesis of Zn PcF12-SH

We followed the procedure shown in Scheme S1 to synthesis the **Zn PcF12-SH** compound used in the present study.

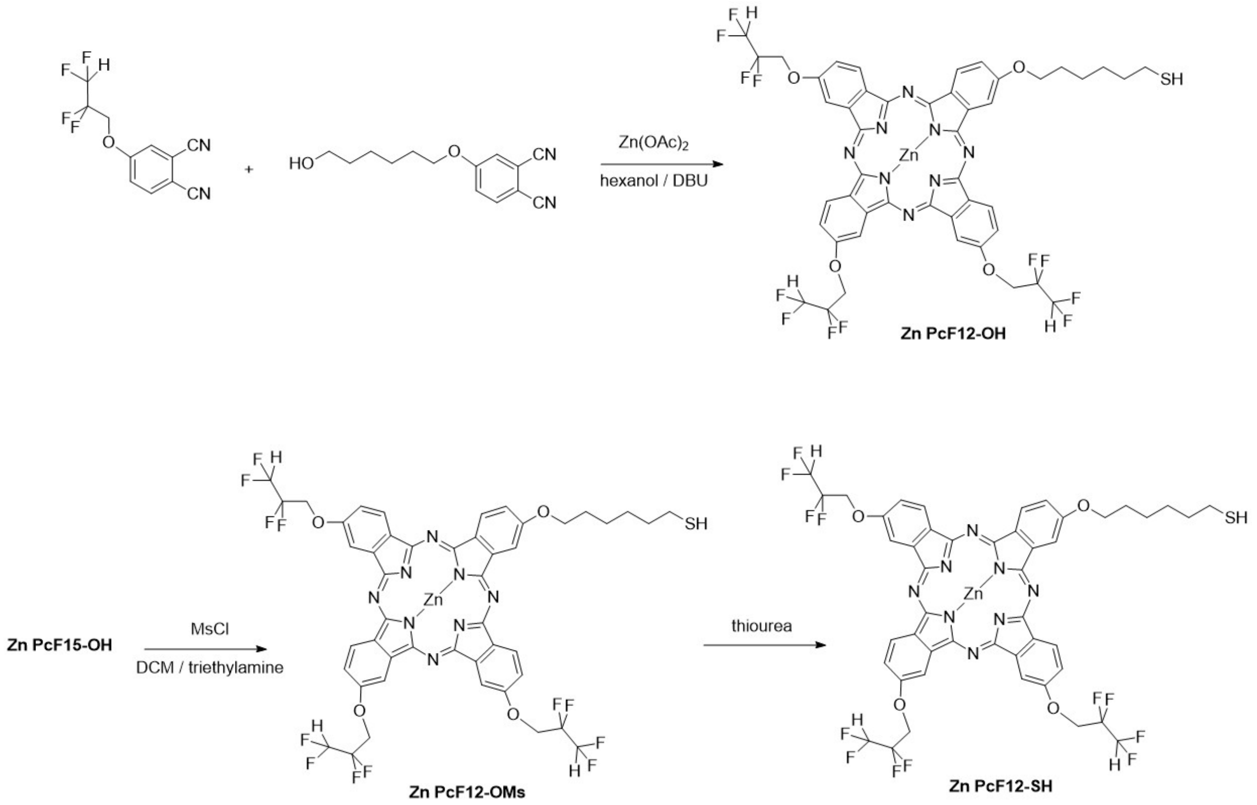

Scheme S1: The procedure used in the present study for synthesis of **Zn PcF12-SH**.

Synthesis of 2(5), 9(12), 16(19)-tri(2,2,3,3-tetrafluoropropoxy)-23-(6-hydroxy hexyl)oxy-phthalocyanine Zn PcF12-OH: 4-[(6-hydroxy hexyl)oxy]phthalonitrile (0.3 g, 1.2 mmol), 4-(2,2,3,3-tetrafluoropropoxy) phthalonitrile (1,50 g, 5,8 mmol), zinc acetate (0,45 g, 2,44 mmol), hexanol (5 mL) and DBU (0.5 mL) were refluxed under argon for 48 h. The green reaction mixture was then poured into hexane (50 mL) and the resulting precipitate filtered. The resulting solid was dissolved in a minimum amount of dichloromethane and precipitated several times from hot *n*-hexane. The crude product was loaded on a silica gel column, and first eluted by dichloromethane, then a 20/1 dichloromethane / methanol solution to elute the symmetric phthalocyanine, followed by a 10/1 solutionto obtain the

desired asymmetric phthalocyanine **PcF15-OH** as a dark green solid. Yield: 35%. FT-IR ν (cm^{-1}): 3464 (OH), 2935-2862, 1607, 1487, 1394, 1338, 1226, 1099, 1089, 943. ^1H NMR ($CDCl_3$) ppm: 7.71 (d, 4H, ArCH), 7,43 (b, 4H, ArCH), 7,12 (d, 4H, ArCH), 6.95-6.69 (m, 3H, CF_2**CH**), 4.10-4.06 (t, 6H, O**CH_2**CF_2), 3.67 (b, 2H, **CH_2**O), 3.45 (b, 2H, **CH_2**-OH), 1.76-1.45 (m, 8H, **CH_2**). MALDI-TOF-MS (without matrix) m/z: 1084.98 $[M]^+$. Calc. for $C_{47}H_{34}F_{12}N_8O_5Zn$ (1084.22): C 52.07; H 3.16; N 10.33; Found: C 51.75; H 3.11; N 10.06. 4.08, 3.68, 3.45, 3.36, 3.20, 2.53.

Synthesis of [2(5), 9(12), 16(19)-tri(2,2,3,3-Tetrafluoropropoxy)-23-(6-methan esulfonyloxyhexyloxy)]-phthalocyanine Zn PcF12-OMs: Triethylamine (20 mL) and methanesulfonyl chloride (20 mL) were added with stirring to an ice-cooled solution of **PcF15-OH** (0.3 g, 0.28 mmol) in dichloromethane (40 mL). The mixture was allowed to warm up to room temperature and stirring was continued for 24 h. Then the resulting solution was washed with water and dried with anhydrous sodium sulfate. The product was purified by column chromatography (silica gel) with a 20:1 mixture of dichloromethane and methanol as eluent. **PcF15-OMs** was obtained as a dark blue solid. Yield: 64%. FT-IR ν (cm^{-1}): 3070, 2941-2862, 1607, 1480, 1456, 1397, 1336, 1224, 1172 (O=S=O), 1098, 1039, 945, 829. ^1H NMR (acetone d_6) ppm: 8.01 (d, 4H, ArCH), 7,43 (b, 4H, ArCH), 7,08 (d, 4H, ArCH), 6.20-6.12 (m, 3H, CF_2**CH**), 4.51-4.46 (t, 6H, O**CH_2**CF_2), 4.10-4.07 (t, 2H, **CH_2**O), 3. 29 (t, 2H, **CH_2**OSO_2), 3.01 (s, 3H, SO_2**CH_3**), 1.62–1.42 (m, 8H, CH_2). MALDI-TOF-MS (2,5-dihydroxybenzoic acid) m/z: 1162.53 $[M]^+$. Calc. for $C_{48}H_{36}F_{12}N_8O_7SZn$ (1162.31): C 49.60; H 3.12; N 9.64; Found: C 49.39; H 3.03; N 9.56.

Synthesis of [2(5), 9(12), 16(19)-tri(2,2,3,3-Tetrafluoropropoxy)-23-[(6- merca ptohexyloxy]-phthalocyanine Zn PcF12-SH: PcF15-Oms (0.3 g, 0.26 mmol), thiourea (0.13 g, 1.64 mmol), THF (25 mL) and ethanol (7 mL) were refluxed for 48 h. Then aqueous sodium hydroxide solution (20%, 15.5 mL) was added. After 24 h, the reacting mixture was poured into ice-water and added dilute hydrochloride acid (1.2 M) (pH = 2) and extracted with dichloromethane. The organic phase was separated and dried with anhydrous sodium

sulfate. **PcF15-SH** was obtained as a greenish blue solid. Yield: 30%. FT-IR ν (cm^{-1}): 3040, 2931-2850, 2595 (SH), 1608, 1487, 1455, 1392, 1338, 1283, 1222, 1200, 1091, 1069, 1039, 944, 832. ^{1}H NMR (acetone d_6) δ ppm: 8.10 (d, 4H, ArCH), 7.84 (d, 4H, ArCH), 7.40 (b, 4H, ArCH), 6.12-5.95 (m, 3H, CF_2**CH**), 4.51-4.47 (t, 6H, O**CH$_2$**CF_2), 3.70 (t, 2H, **CH$_2$**-O), 3.40 (b, 1H, **SH**), 1.56–1.25 (m, 10H, **CH$_2$**). MALDI-TOF-MS matrix DHB m/z (%): 1101.01 $[M+H]^+$, 1067.88 $[M-SH]^+$, 1038.183 $[M-S-2(C_2H_4)]^+$, 981.892 $[M-S-2(C_6H_{12})]^+$. Calc. for $C_{47}H_{34}F_{12}N_8O_4SZn$ (1100.28): C 51.31; H 3.11; N 10.18; Found: C 51.17; H 3.08; N 10.12.

3 Absorption Spectroscopy of Different Regioisomers of Zn Pc Monomer

3.1 Fundamental Absorption Properties

The Zn Pc studied in this work (i.e. **Zn PcF12-SH**), in its truncated model with lateral OCH_3 groups, features 36 regioisomers, resulting from single substitution of one of the non-peripheral or peripheral positions available on each isoindole units (see Figure S7). Therefore, we initially considered all the regioisomers and computed their relative stability and also their excited state properties for the lowest electronic transitions at FC point, as reported in Tables S3-S5. The results show that different regioisomers slightly affect the relative stability of the monomeric forms (see Table S3) and also the energy splitting between the excited states (at most 0.1 eV, see Table S4). For most of the regioisomers, the low-lying excited states S_1 and S_2 feature quasi-degenerate bright states dominated by H→L and H→L+1 transitions, respectively, with more than 90% contributions. The vertical energy gap amounts to 0.04 eV for average values of 1.90 (S_1) and 1.94 eV (S_2), with standard deviation of $\pm$0.02 eV for both states, as reported in Table S5. The next transition for all regioisomers appears $\sim$1.5 eV higher than the first two states, and out of Q-band region. Taking into account the full structure, however, the steric restriction imposed by the introduction of the bulky substituents in the non-peripheral positions excludes the regioisomers carrying OCH_3 group at the α positions. In other words, with the precursors we used in the experiment, only four regioisomers A, B, C and D are possible whose vibronic absorption spectra are calculated and discussed in the next section.

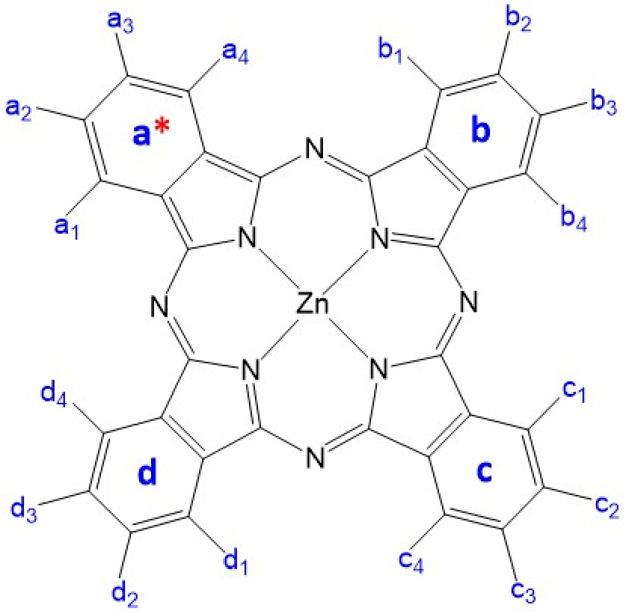

Figure S7: The general scheme used in this study for nomenclature of Zn Pc regioisomers. "*" points to the starting point of nomenclature.

Table S3: The relative energies (RE, in kcal/mol) of different regioisomers of the truncated **Zn PcF12-SH** monomer considered in this study, computed at CAM-B3LYP/6-31G(d,p)//PCM(DMSO) level of theory.

Isomer	RE	Isomer	RE	Isomer	RE	Isomer	RE
$a_3b_2c_3d_3$ (A)	0.000	$a_1b_1c_4d_2$	4.629	$a_1b_2c_3d_4$	3.720	$a_1b_3c_3d_2$	2.849
$a_3b_2c_3d_2$ (B)	0.750	$a_1b_1c_4d_4$	5.320	$a_1b_2c_4d_3$	3.272	$a_1b_3c_3d_3$	3.115
$a_2b_2c_3d_3$ (C)	0.624	$a_1b_2c_1d_2$	3.606	$a_1b_3c_1d_1$	4.606	$a_1b_3c_4d_1$	4.346
$a_2b_2c_2d_2$ (D)	2.174	$a_1b_2c_1d_3$	3.692	$a_1b_3c_1d_3$	3.897	$a_1b_3c_4d_3$	3.581
$a_1b_1c_1d_1$	5.010	$a_1b_2c_2d_3$	3.001	$a_1b_3c_2d_1$	3.741	$a_1b_4c_1d_2$	4.514
$a_1b_1c_1d_2$	4.265	$a_1b_2c_2d_4$	3.785	$a_1b_3c_2d_2$	3.192	$a_1b_4c_1d_4$	5.435
$a_1b_1c_2d_2$	3.271	$a_1b_2c_3d_1$	3.631	$a_1b_3c_2d_3$	2.956	$a_1b_4c_2d_2$	3.714
$a_1b_1c_2d_4$	4.761	$a_1b_2c_3d_2$	2.881	$a_1b_3c_2d_4$	3.874	$a_1b_4c_3d_1$	4.318
$a_1b_1c_4d_1$	5.303	$a_1b_2c_3d_3$	2.654	$a_1b_3c_3d_1$	3.550	$a_1b_4c_3d_2$	3.831

Table S4: The vertical excitation energies (E in eV), oscillator strengths (f) and the molecular orbitals (MOs) involved in the electronic transitions, which have been computed at TD-CAM-B3LYP/6-31G(d,p) level of theory, adopting LR-PCM(DMSO) model of solvation, for the lowest excited states of all regioisomers of the Zn Pc monomer constructed according to Figure S7. For comparison, the relevant properties for the full structure (i.e. **Zn PcF12-SH**) are also included.

isomer	State	E(eV)	f	Cont. MOs	W(%)	isomer	State	E(eV)	f	Cont. MOs	W(%)
Full structure	S_1	1.963	0.706	H→L	86.25	$a_1b_4c_3d_2$	S_1	1.897	0.669	H→L	95.70
	S_2	1.976	0.710	H→L+1	86.13		S_2	1.932	0.766	H→L+1	95.57
$a_3b_2c_3d_3$ (A)	S_1	1.933	0.674	H→L	95.32	$a_1b_2c_2d_3$	S_1	1.909	0.725	H→L	95.13
	S_2	1.979	0.681	H→L+1	94.97		S_2	1.956	0.697	H→L+1	94.66
$a_3b_2c_3d_2$ (B)	S_1	1.895	0.680	H→L	95.83	$a_1b_2c_2d_4$	S_1	1.917	0.688	H→L+1	92.08
	S_2	1.986	0.697	H→L+1	95.07		S_2	1.921	0.744	H→L	92.22
$a_2b_2c_3d_3$ (C)	S_1	1.949	0.687	H→L	29.95	$a_1b_2c_3d_1$	S_1	1.901	0.705	H→L	95.75
				H→L+1	65.46		S_2	1.951	0.721	H→L+1	95.50
	S_2	1.955	0.688	H→L	65.58	$a_1b_4c_2d_2$	S_1	1.917	0.753	H→L	90.33
				H→L+1	29.95		S_2	1.926	0.681	H→L+1	90.17
$a_2b_2c_2d_2$ (D)	S_1	1.920	0.720	H→L	95.70	$a_1b_2c_3d_2$	S_1	1.878	0.689	H→L	95.80
	S_2	1.920	0.720	H→L+1	95.70		S_2	1.955	0.734	H→L+1	95.27
$a_1b_1c_1d_1$	S_1	1.905	0.714	H→L	92.18	$a_1b_2c_3d_3$	S_1	1.907	0.703	H→L	94.81
	S_2	1.905	0.714	H→L+1	92.18		S_2	1.952	0.715	H→L+1	94.38
$a_1b_1c_1d_2$	S_1	1.887	0.709	H→L	95.41	$a_1b_2c_3d_4$	S_1	1.899	0.669	H→L	95.69
	S_2	1.935	0.716	H→L+1	95.10		S_2	1.948	0.756	H→L+1	95.61
$a_1b_1c_2d_2$	S_1	1.916	0.714	H→L	85.20	$a_1b_2c_4d_3$	S_1	1.876	0.726	H→L	95.94
	S_2	1.921	0.712	H→L+1	85.17		S_2	1.961	0.695	H→L+1	95.23
$a_1b_1c_2d_4$	S_1	1.887	0.728	H→L	95.81	$a_1b_3c_1d_1$	S_1	1.889	0.729	H→L	95.79
	S_2	1.936	0.702	H→L+1	95.42		S_2	1.938	0.704	H→L+1	95.42
$a_1b_1c_4d_1$	S_1	1.905	0.751	H→L	95.03	$a_1b_3c_1d_3$	S_1	1.866	0.742	H→L	96.01
	S_2	1.908	0.683	H→L+1	94.85		S_2	1.964	0.693	H→L+1	95.20
$a_1b_1c_4d_2$	S_1	1.890	0.728	H→L	95.71	$a_1b_3c_2d_1$	S_1	1.896	0.705	H→L	95.77
	S_2	1.939	0.703	H→L+1	95.36		S_2	1.933	0.727	H→L+1	95.48
$a_1b_1c_4d_4$	S_1	1.906	0.716	H→L	94.12	$a_1b_3c_2d_2$	S_1	1.894	0.729	H→L	95.81
	S_2	1.907	0.718	H→L+1	94.11		S_2	1.944	0.701	H→L+1	95.39
$a_1b_2c_1d_2$	S_1	1.869	0.710	H→L	95.84	$a_1b_3c_2d_3$	S_1	1.881	0.722	H→L	95.97
	S_2	1.960	0.717	H→L+1	95.16		S_2	1.960	0.706	H→L+1	95.32
$a_1b_2c_1d_3$	S_1	1.876	0.730	H→L	95.93	$a_1b_3c_2d_4$	S_1	1.895	0.741	H→L	95.87
	S_2	1.961	0.696	H→L+1	95.21		S_2	1.933	0.691	H→L+1	95.40
$a_1b_3c_3d_1$	S_1	1.915	0.715	H→L	89.00	$a_1b_3c_3d_2$	S_1	1.892	0.710	H→L	95.75
	S_2	1.921	0.712	H→L+1	88.99		S_2	1.941	0.715	H→L+1	95.41
$a_1b_3c_3d_3$	S_1	1.896	0.730	H→L	95.85	$a_1b_3c_4d_1$	S_1	1.885	0.710	H→L	95.51
	S_2	1.945	0.706	H→L+1	95.40		S_2	1.933	0.714	H→L+1	95.20
$a_1b_3c_4d_3$	S_1	1.863	0.723	H→L	95.96	$a_1b_4c_1d_2$	S_1	1.888	0.702	H→L	94.80
	S_2	1.961	0.703	H→L+1	95.21		S_2	1.936	0.732	H→L+1	94.55
$a_1b_4c_1d_4$	S_1	1.902	0.788	H→L	95.68						
	S_2	1.908	0.650	H→L+1	95.31						
$a_1b_4c_3d_1$	S_1	1.888	0.710	H→L	95.49						
	S_2	1.937	0.718	H→L+1	95.20						

Table S5: The average values and the standard deviations for the vertical energies (E) and oscillator strengths (f) of the lowest excited states, taken over all the 36 regioisomers reported in Table S4.

State	E (eV)	f
S_1	1.897±0.018	0.715±0.024
S_2	1.941±0.019	0.709±0.021

3.2 Adiabatic Vibronic Spectra

The experimental absorption spectrum of **Zn PcF12-SH** in diluted solutions of DMSO, is displayed in Figure S8 in the full frequency range. It exhibits a relatively broad Soret band in 2.76-4.13 eV (300-450 nm) with the maximum at 3.52 eV (352 nm) and another more intense band in 1.65-2.25 eV (550-750 nm) peaking at 1.83 eV (679 nm) which has been assigned to Q-band. The latter intense band is known to be the most important characteristic of the monomeric Zn Pc species. The Q-band is associated with a relatively weaker peak in higher energy side (at ∼2 eV), which has been attributed to vibrational progression of Q-band and also, small contribution from aggregated **Zn PcF12-SH** species which might be only partially formed in DMSO. Both Soret and Q bonds are indicative of $\pi\pi^*$ transitions, as common features of this category of the compounds.

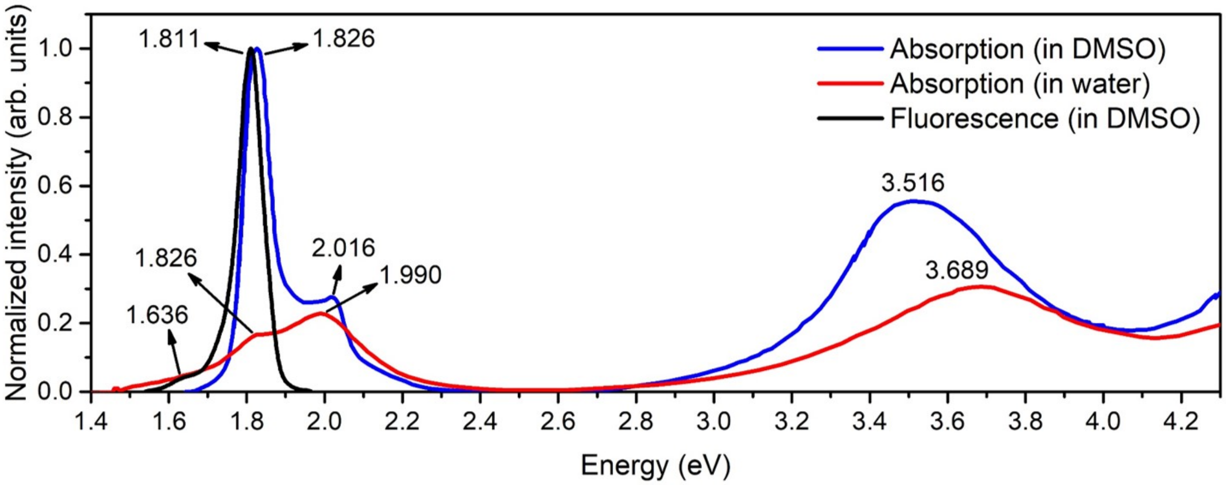

Figure S8: The experimental absorption and emission spectra of the diluted solutions of **Zn PcF12-SH** in DMSO and water in the full frequency range. The intensity of all spectra is normalized to match the maximum intensity of the Q-band in DMSO.

Here we focus on Q-band, since absorption in this region is most affected by aggregation and importantly, fluorescense is registered with excitation falling in this range (1.65-1.80 eV). The spectral shape of the Q band cannot be properly described at pure electronic level, and therefore in the following we run vibronic computations. Figure S9 represents the vibronic absorption spectra computed for the possible regioisomers of truncated **Zn PcF12-**

SH monomer in adiabatic approximation with the FC|VG model, and compares them with the experimental absorption spectrum in Q-band region. Although the computed spectra reveal some influences of different regioisomers on the spectral lineshape, the main features of the experimental absorption spectrum are nicely reproduced by all of them, in particular the vibronic structure at ∼2 eV, which is accompanied with a weak progression at ∼2.15 eV. Compared to other regioisomers, the spectrum predicted for regioisomer B exhibits the most appreciable differences with the experiment, indicating the splitting of the lowest peak and an increase of the relative intensity of two peaks. These differences are originated from a notable energy gap of ∼0.1 eV between the two lowest electronic transitions of this regioisomer, as evidenced in Table S4. For the regioisomers A and D, however, the differences are mostly reflected in some slight changes in the relative intensity of the lowest energy peak with respect to the vibronic peak.

Remarkably, the noticeable differences between the regioisomers will wipe out to a large extent by averaging over the four vibronic spectra computed for the regioisomers A-D (see the spectrum shown in orange line in Figure S9), implying various regioisomers can coexist and contribute to the final spectral line shape. Among the possible regioisomers of truncated **Zn PcF12-SH**, the isomer C delivers the spectrum which resembles more the average and the experiment, concerning the spectral lineshape, spacing and relative intensity of the bands. Notably, substituting the OCH_3 groups with the long lateral chains in regioisomer C does not introduce any significant change on the spectral line shape (except for an extra rigid blue shift of just ∼0.02 eV), as evidenced in Figure S10, confirming the reliability of the truncated model of this regioisomer to simulate the spectroscopy of **Zn PcF12-SH**. Furthermore, the regioisomer C features remarkably different optical properties compared to other regioisomers studied here. In fact as evidenced in Table S4, the two low-lying bright states of this regioisomer are combinations of H→L and H→L+1 transitions, which in a full-symmetric chromophore of Zn Pc (with D_{4h} point group) constitute a pair of degenerate E_u states. Here, the lateral groups attached to isoindole units have removed the molecular sym-

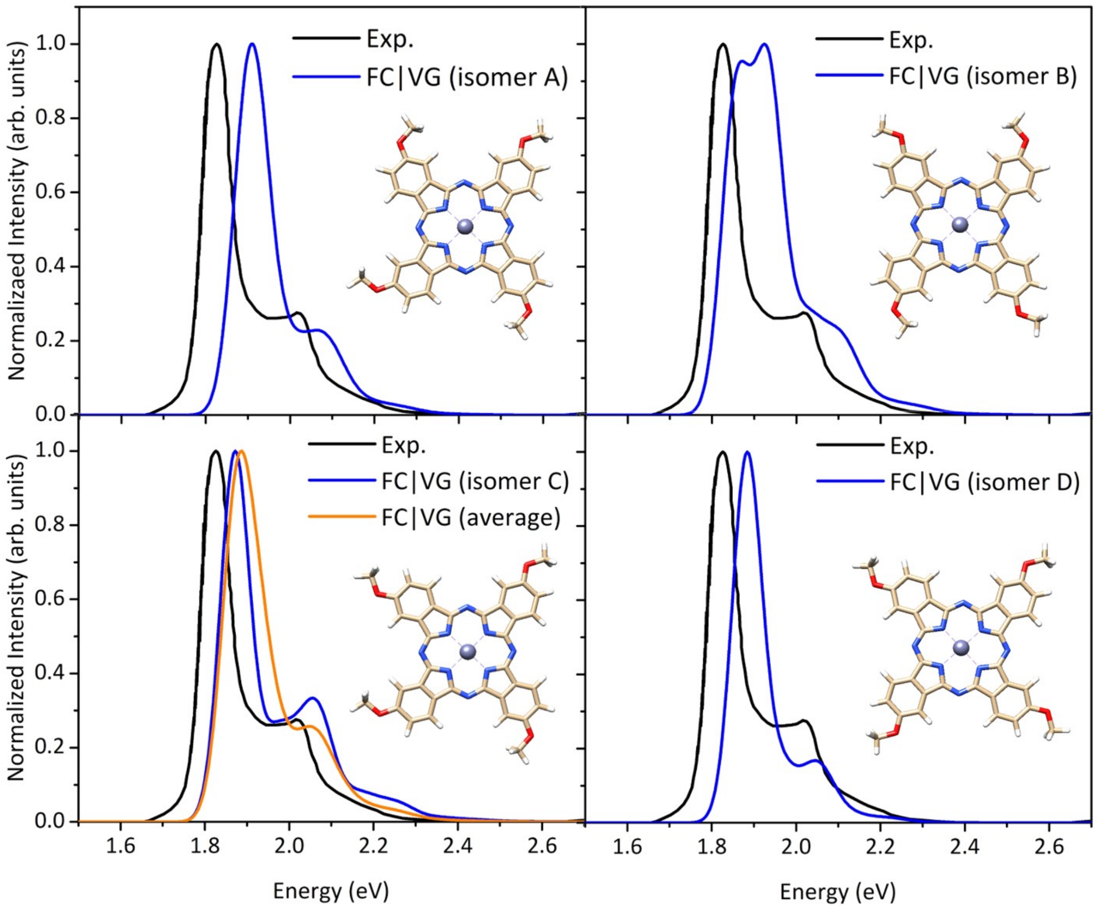

Figure S9: Comparison of the adiabatic vibronic FC|VG absorption spectra computed for the selected regioisomers (A, B, C and D) of truncated **Zn PcF12-SH** monomer with the experiment in DMSO. The average spectrum (orange) is shown in panel of isomer C.

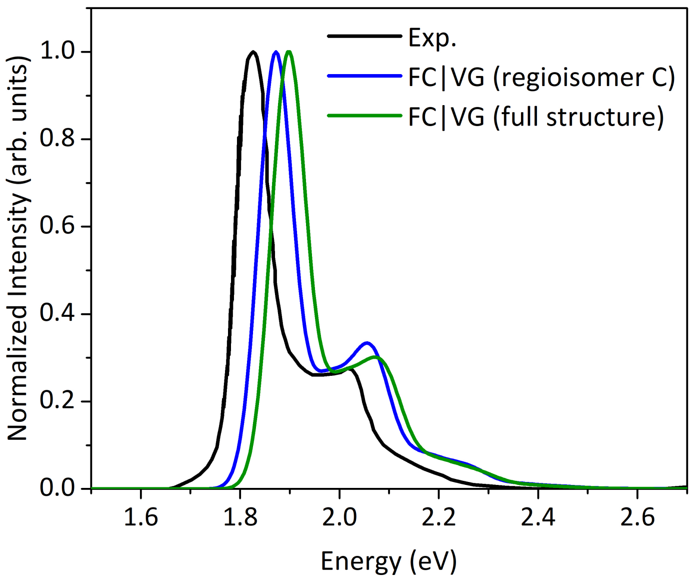

Figure S10: Comparison of the adiabatic vibronic FC|VG spectra computed for regioisomer C with that of full structure. The experimental spectrum (in DMSO) in Q-band region is also represented. To facilitate the comparison, all the spectra were normalized to have the same maximum intensity. All stick transitions were convoluted with a Gaussian function with HWHM = 0.04 eV.

metry, but the π-system still electronically behaves like a D_{4h} system, exhibiting two nearly degenerate states. The states become less mixed and the virtual orbitals slightly change their character with respect to the symmetric case, but looking at Figure S11, it is evident that like in the symmetric Zn Pc chromophore, both L and L+1 orbitals are still identical with a 90 degree rotation. The system is thus potentially able to undergo JT-like non-adiabatic effects. Accordingly, we further exploit the regioisomer C with the adapted position of the substituents to address this effect on the spectroscopy and photoinduced dynamics of both monomeric and aggregate forms of **Zn PcF12-SH**, as described in the main text.

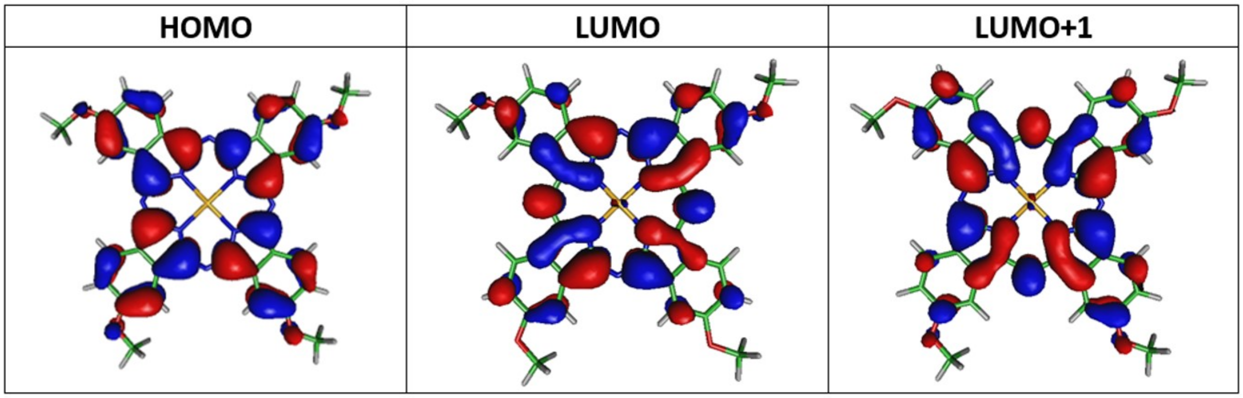

Figure S11: The frontier molecular orbitals involved in electronic transitions for the Zn Pc rgioisomer C, calculated at CAM-B3LYP/6-31G(d,p) level, adopting LR-PCM model for solvation in DMSO.

It is noteworthy that the agreement achieved here for the adiabatic FC|VG spectra with the experiment supports the reliability of harmonic approximation adopted here, implying that the frequency changes and Duschinsky mixing neglected in FC|VG approach play a minor role. This fact is confirmed in Figure S12 by comparing the FC|VG spectrum with the FC|AH one which accounts for these effects. These results also anticipate a negligible role for non-adiabatic interstate couplings in determination of the spectral shape of Zn Pc monomer investigated here, despite of representing two close-lying S_1 and S_2 excited electronic states.

Compared to the vibronic spectrum computed for regioisomer C, the vibronic band in the experimental absorption spectrum features a broad flat region (extended from ~1.90 to ~2.02 eV), which can be probably traced back to a partial contribution from self-assembled

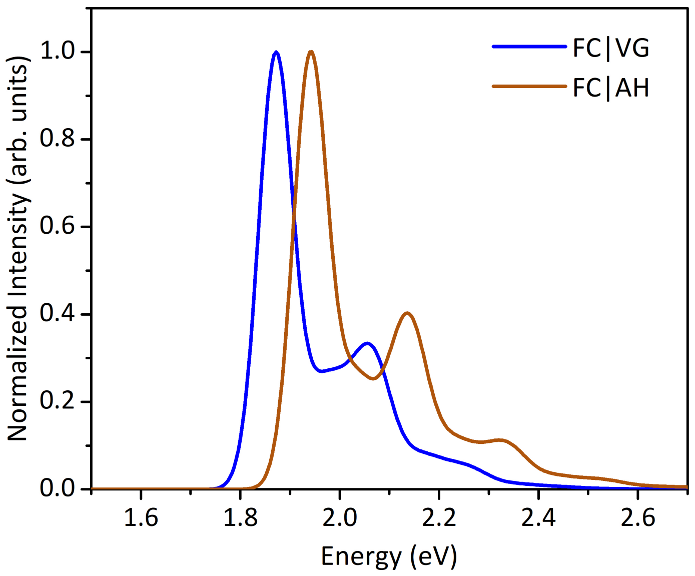

Figure S12: Comparison of the adiabatic vibronic spectra computed for regioisomer C, adopting different FC|VG and FC|AH approaches. To facilitate the comparison, both the spectra were normalized to match the maximum intensity. All stick transitions were convoluted with a Gaussian function with HWHM = 0.04 eV.

Zn PcF12-SH species. This is clearly evident in Figure S13, if one compares the absorption spectrum with the fluorescence excitation spectra recorded in different wavelengths in DMSO. In fact, our experiment have revealed that the **Zn PcF12-SH** in water does not fluorescence, where predominantly exists in self-assembled forms. This suggests formation of aggregated **Zn PcF12-SH** species in mixture with monomeric forms in DMSO. Since these aggregated species are non-emissive, they do not contribute to the excitation spectra, where the flat region has been apparently removed and the vibronic band appeared sharper than that of the absorption spectrum.

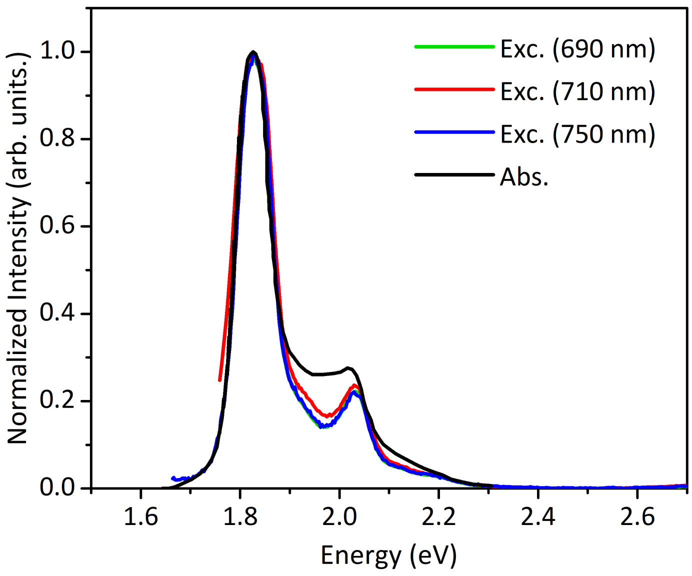

Figure S13: Comparison of the experimental absorption spectrum in Q-band region with the excitation spectra recorded at different wavelengths for monomer of **Zn PcF12-SH** in DMSO. All the spectra were normalized to have the same maximum intensity.

Table S6: The vertical excitation energies (E in eV), oscillator strengths (f) and contributed MOs in transition for the eight lowest excited states of the OM dimer computed at CAM-B3LYP/6-31G(d,p) level of theory, adopting the LR implementation of PCM model in water.

State	E(eV)	f	Cont.	W(%)
S_1	1.824	0.009	H→L	24.21
			H→L+1	21.86
			H-1→L+1	18.60
			H-1→L	8.71
			H→L+3	8.03
S_2	1.837	0.010	H→L	27.48
			H-1→L	14.63
			H-1→L+1	12.04
			H→L+1	11.14
S_3	1.947	0.310	H-1→L	47.27
			H→L+1	46.70
S_4	1.960	0.398	H-1→L+1	42.25
			H→L	41.69
S_5	2.020	0.003	H→L+2	38.43
			H-1→L+1	21.60
			H-1→L+3	21.37
S_6	2.027	0.014	H→L+3	32.54
			H-1→L+2	22.72
			H→L+1	13.77
			H-1→L	12.80
			H-1→L+1	12.53
S_7	2.148	0.639	H-1→L+2	49.41
			H→L+3	34.71
S_8	2.172	0.633	H-1→L+3	55.45
			H→L+2	30.54

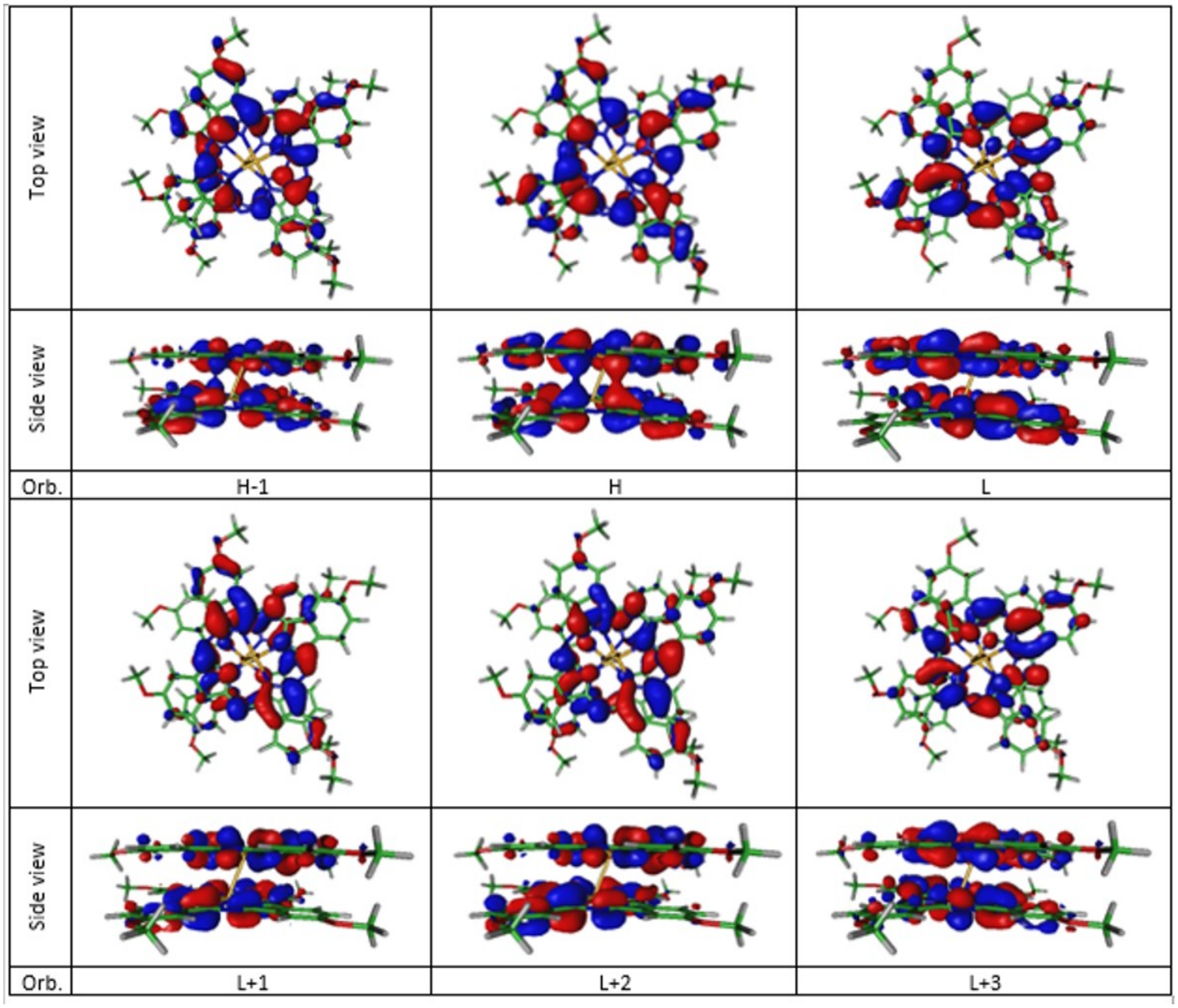

Figure S14: The frontier molecular orbitals involved in electronic transitions for the OM dimer, calculated at TD-CAM-B3LYP/6-31G(d,p) level adopting LR-PCM model of solvation in water. The molecular orbitals are represented in both top and side views.

Table S7: LVC Hamiltonian in the diabatic representation for the OM dimer. Parametrization at TD-CAM-B3LYP/6-31G(d,p)/LR-PCM(water) level of theory. The matrix is symmetric and only half is reported. Terms in the diagonal represent the vertical energies, $E^d_{ii}(0)$, while the off diagonal terms are the constant coupling, $E^d_{ij}(0)$. All terms are in eV.

State	$\|L_1\rangle$	$\|L'_1\rangle$	$\|L_2\rangle$	$\|L'_2\rangle$	$\|CT(1 \to 2)\rangle$	$\|CT'(1 \to 2)\rangle$	$\|CT(2 \to 1)\rangle$	$\|CT'(2 \to 1)\rangle$
$\|L_1\rangle$	1.983	0.004	0.089	0.065	0.032	-0.050	-0.028	-0.021
$\|L'_1\rangle$		1.984	0.071	-0.083	-0.061	-0.010	0.029	-0.039
$\|L_2\rangle$			1.979	0.004	-0.028	-0.028	0.020	-0.064
$\|L'_2\rangle$				1.983	0.014	-0.019	-0.048	-0.012
$\|CT(1 \to 2)\rangle$					2.072	0.011	-0.023	-0.001
$\|CT'(1 \to 2)\rangle$						2.064	0.001	0.001
$\|CT(2 \to 1)\rangle$							2.076	-0.008
$\|CT'(2 \to 1)\rangle$								2.059

Table S8: LVC Hamiltonian in the diabatic representation for the DM dimer. Parametrization at TD-CAM-B3LYP/6-31G(d,p)/LR-PCM(water) level of theory. The matrix is symmetric and only half is reported. Terms in the diagonal represent the vertical energies, $E^d_{ii}(0)$, while the off diagonal terms are the constant coupling, $E^d_{ij}(0)$. All terms are in eV.

State	$\|L_1\rangle$	$\|L'_1\rangle$	$\|L_2\rangle$	$\|L'_2\rangle$	$\|CT(1 \to 2)\rangle$	$\|CT'(1 \to 2)\rangle$	$\|CT(2 \to 1)\rangle$	$\|CT'(2 \to 1)\rangle$
$\|L_1\rangle$	1.955	0.000	0.048	0.039	0.001	-0.001	-0.002	-0.001
$\|L'_1\rangle$		1.966	0.042	-0.047	-0.003	-0.001	0.001	-0.003
$\|L_2\rangle$			1.953	0.000	-0.002	-0.001	0.001	-0.002
$\|L'_2\rangle$				1.957	0.001	-0.002	-0.002	-0.002
$\|CT(1 \to 2)\rangle$					2.342	0.008	0.000	0.000
$\|CT'(1 \to 2)\rangle$						2.357	0.000	0.000
$\|CT(2 \to 1)\rangle$							2.337	-0.003
$\|CT'(2 \to 1)\rangle$								2.358

Table S9: The properties of the adiabatic LVC states, including the composition, energy and oscillator strength, at CAM-B3LYP/6-31G(d,p)//LR-PCM(water) level, for the OM and DM dimer models.

State	Character	S_1	S_2	S_3	S_4	S_5	S_6	S_7	S_8
OM Zn Pc dimer									
$\|L_1\rangle = \|H_1 \to L_1\rangle$	LE	0.467	0.000	0.226	0.002	0.009	0.000	0.295	0.001
$\|L_1'\rangle = \|H_1 \to (L+1)_1\rangle$	LE	0.003	0.497	0.022	0.204	0.017	0.000	0.001	0.255
$\|L_2\rangle = \|H_2 \to L_2\rangle$	LE	0.349	0.181	0.166	0.001	0.000	0.010	0.140	0.153
$\|L_2'\rangle = \|H_2 \to (L+1)_2\rangle$	LE	0.122	0.298	0.002	0.351	0.004	0.004	0.141	0.078
$\|CT(1 \to 2)\rangle = \|H_1 \to L_2\rangle$	CT	0.030	0.015	0.000	0.190	0.259	0.264	0.032	0.210
$\|CT'(1 \to 2)\rangle = \|H_1 \to (L+1)_2\rangle$	CT	0.004	0.000	0.225	0.041	0.166	0.370	0.177	0.016
$\|CT(2 \to 1)\rangle = \|H_2 \to L_1\rangle$	CT	0.002	0.007	0.007	0.186	0.510	0.017	0.098	0.173
$\|CT'(2 \to 1)\rangle = \|H_2 \to (L+1)_1\rangle$	CT	0.023	0.002	0.351	0.024	0.034	0.335	0.116	0.114
	ΣLE	0.940	0.976	0.417	0.558	0.031	0.014	0.577	0.487
	ΣCT	0.060	0.024	0.583	0.442	0.969	0.986	0.423	0.513
	f	0.019	0.001	**0.440**	**0.534**	0.006	0.009	**0.628**	**0.625**
	E (in eV)	1.861	1.867	1.987	2.002	2.052	2.070	2.167	2.194
DM Zn Pc dimer									
$\|L_1\rangle = \|H_1 \to L_1\rangle$	LE	0.410	0.089	0.500	0.001	0.000	0.000	0.000	0.000
$\|L_1'\rangle = \|H_1 \to (L+1)_1\rangle$	LE	0.074	0.381	0.000	0.544	0.000	0.000	0.000	0.000
$\|L_2\rangle = \|H_2 \to L_2\rangle$	LE	0.483	0.048	0.264	0.205	0.000	0.000	0.000	0.000
$\|L_2'\rangle = \|H_2 \to (L+1)_2\rangle$	LE	0.034	0.482	0.236	0.249	0.000	0.000	0.000	0.000
$\|CT(1 \to 2)\rangle = \|H_1 \to L_2\rangle$	CT	0.000	0.000	0.000	0.000	0.008	0.830	0.000	0.162
$\|CT'(1 \to 2)\rangle = \|H_1 \to (L+1)_2\rangle$	CT	0.000	0.000	0.000	0.000	0.001	0.161	0.002	0.836
$\|CT(2 \to 1)\rangle = \|H_2 \to L_1\rangle$	CT	0.000	0.000	0.000	0.000	0.971	0.009	0.019	0.000
$\|CT'(2 \to 1)\rangle = \|H_2 \to (L+1)_1\rangle$	CT	0.000	0.000	0.000	0.000	0.019	0.000	0.978	0.002
	ΣLE	1.000	1.000	1.000	1.000	0.000	0.000	0.000	0.000
	ΣCT	0.000	0.000	0.000	0.000	1.000	1.000	1.000	1.000
	f	0.002	0.001	**1.083**	**1.077**	0.000	0.001	**0.000**	**0.000**
	E (in eV)	1.892	1.899	2.017	2.024	2.337	2.338	2.359	2.360

4 Simulated Absorption Spectrum of a Zn Pc Trimeric Species

To investigate the dependence of optical properties of Zn Pc aggregates on the number of molecular units involved in the aggregation, we computed the TD-DFT absorption spectrum of a Zn Pc trimeric species and compared it with that of OM dimer, as shown in Figure S15. In order to generate a truncated trimer model for the **Zn PcF12-SH**, we started from OM dimer and added another Zn Pc monomeric unit to reach a H-type trimer structure. This structure was then fully optimized at the CAM-B3LYP-D3/6-31G(d,p) level adopting LR-PCM/water implicit solvation model. To obtain the absorption spectrum, excited states TD-DFT computations were performed employing the same DFT functional and basis set. The result (while missing vibronic and electronic coupling contributions) shows that trimeric species contributes to the absorption spectrum by altering the relative intensities of the absorption spectrum bands and by adding new peaks with small intensity in the low-energy portion of the Q-band. Therefore, it is expected that accounting for higher order aggregates would increase the relative intensity height of the red tail of the Q-band, while featuring an overall broader and lower band with respect to dimers.

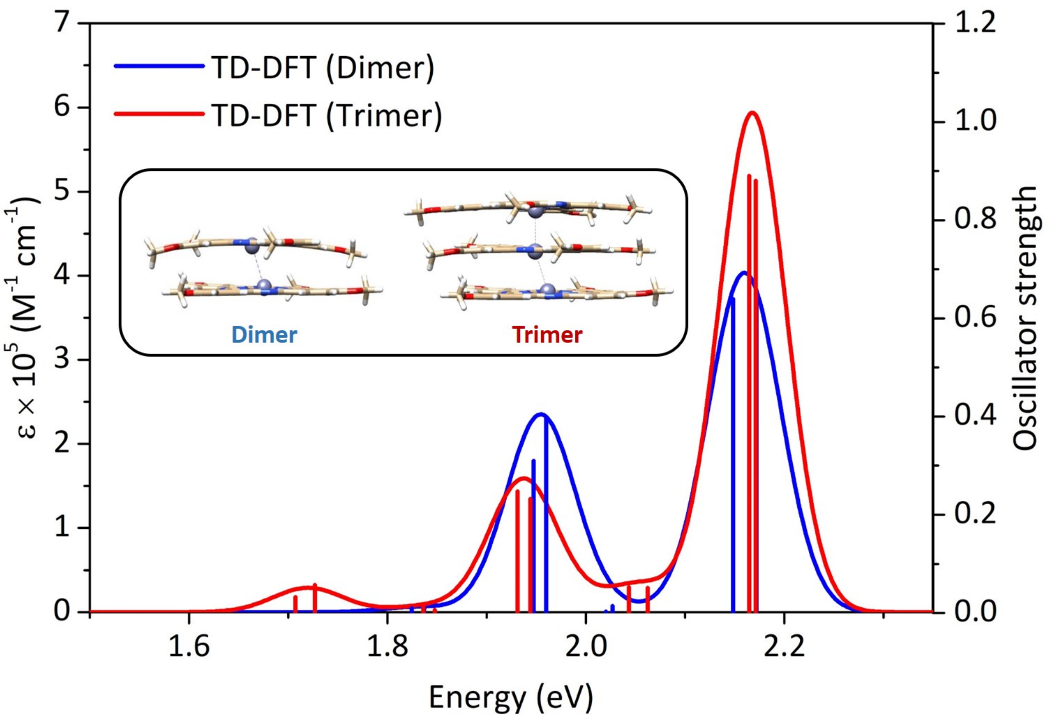

Figure S15: Comparison of the pure electronic TD-DFT spectra computed for a Zn Pc trimer with that of OM dimer. All the stick transitions were convoluted with a Gaussian function with HWHM = 0.04 eV. No shift has been applied to the computed spectra.

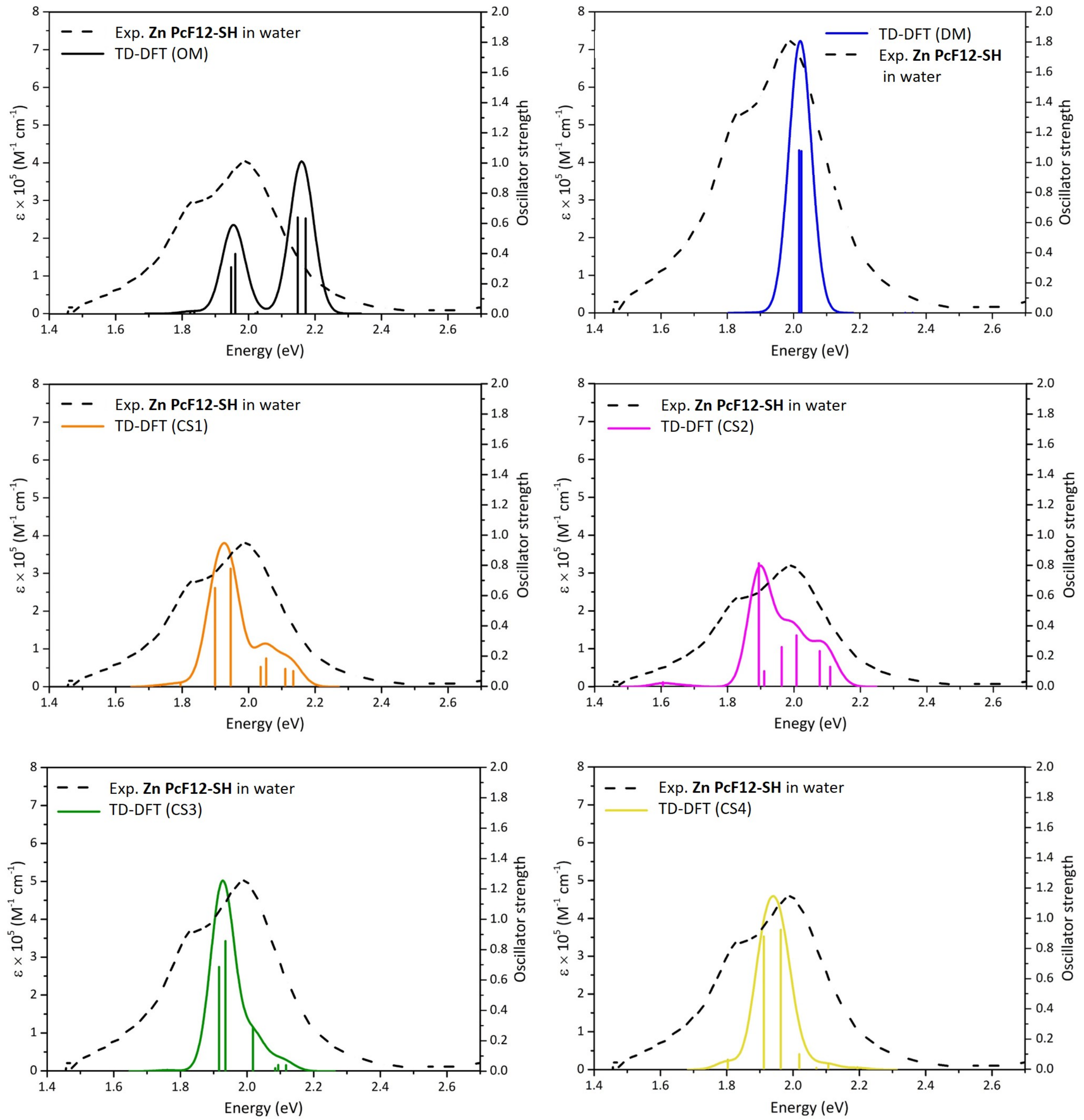

Figure S16: Comparison of the pure electronic TD-DFT spectra computed for all the Zn Pc dimers considered in this study in Q-band region, with the experimental spectrum recorded for **Zn PcF12-SH** in water. All the stick transitions were convoluted with a Gaussian function with HWHM = 0.04 eV. For simplicity of comparison, the experimental spectrum was normalized in each panel to match the maximum intensity of theoretical spectrum. No shift has been applied to the spectra.

5 Definition of Intermolecular Coordinates For Zn Pc Dimers

The intermolecular dimer coordinates employed in this work are defined based on the four important parameters. As indicated in Figure S17a, we defined the d_{Zn-Zn} distance as the distance between the zinc atoms located on the interacting Zn Pc units. The d_{P-P} distance is also defined as an average interatomic distance along a specific axis normal to each Zn Pc ring. In other words, the d_{P-P} is defined as the distance between two imaginary planes, each passing through one of the Zn Pc macrocycle rings with a average distance respect to all the atoms existing in the same ring (the atoms belonging to lateral chains were excluded). The imaginary planes were defined because the two aromatic macrocycle rings of Zn Pc dimers are not necessary planar and, as indicated in Figure 5c in the main text for the CSs obtained from MD clustering, exhibit significant distortions from planarity. Furthermore, we defined the angles α as the angles between the two zinc atoms with each of the nitrogen atoms located on one of the Zn Pc units, as shown in Figure S17a. The dihedral angle β is also defined according to the Figure S17b, as the angle between the two nitrogen atoms belonging to the two opposite isoindole units on each Zn Pc ring. Due to the distortion of the macrocycle rings and hence, loosing the symmetry, the nitrogen atoms located on each porphyrin ring are not expected to be equivalent. Hence, the eight α angles were defined, each concerns one of the nitrogen atoms located on one of the Zn Pc units. The angles α describe both tilting and displacement of one monomer with respect to the other one in all directions and hence, are strongly intercorrelated with the d_{Zn-Zn} distance, while the dihedral angle β describes the twisting of the two Zn Pc units.

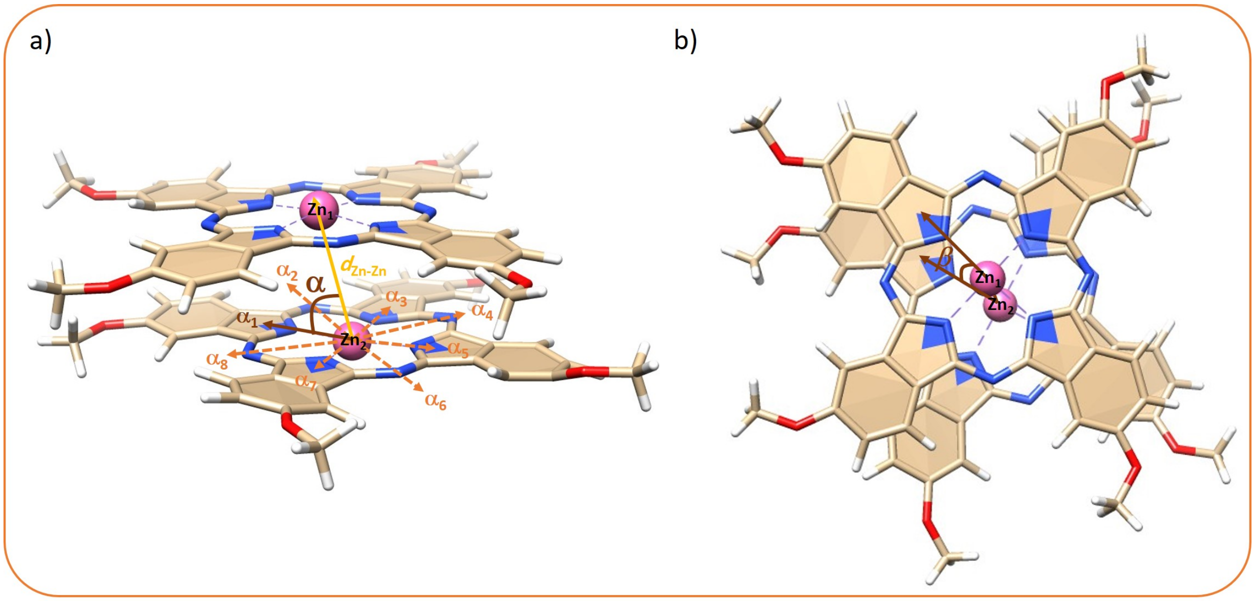

Figure S17: Representation of unit vectors used to define different geometrical descriptors analyzed in this study. a) The structure of a typical Zn Pc dimer, where the arrows define the angles α and the distance between the cores of two Zn Pc units, i.e. d_{Zn-Zn}, describing the tilting and displacement of one monomer with respect to the other and b) the dihedral angle β describing the twisting of two Zn Pc units. The structure of Zn Pc dimer used here was adopted from the CS1.

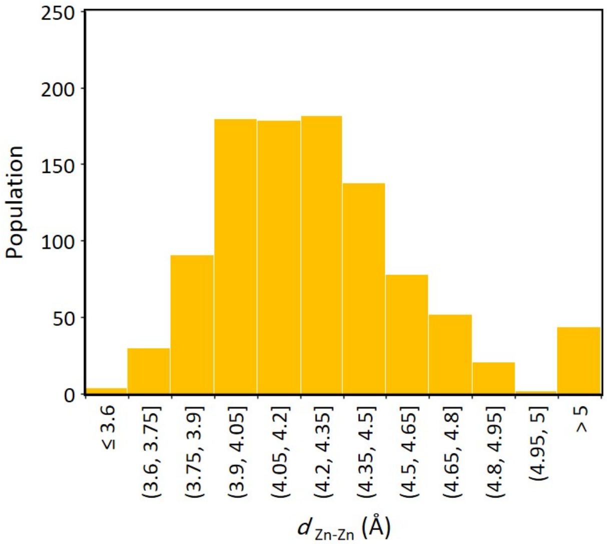

Figure S18: Distributions of intermolecular distance between the Zn Pc cores, d_{Zn-Zn} as defined in Figure S17, computed along the MD trajectories in water.

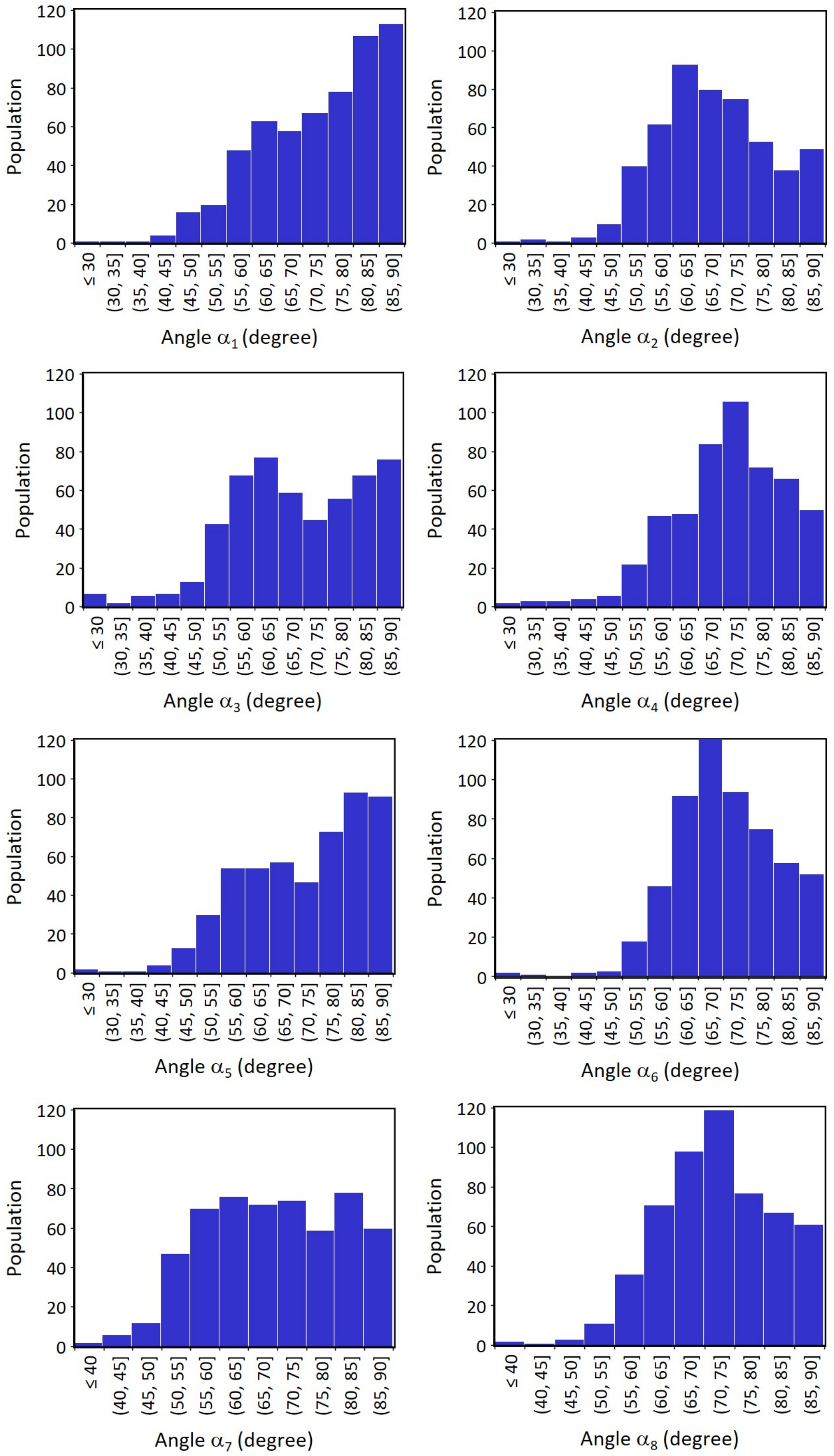

Figure S19: Distributions of intermolecular angles α for each of the nitrogen atoms located on one of the Zn Pc units, according to the numbering shown in Figure S17, computed along the MD trajectories in water.

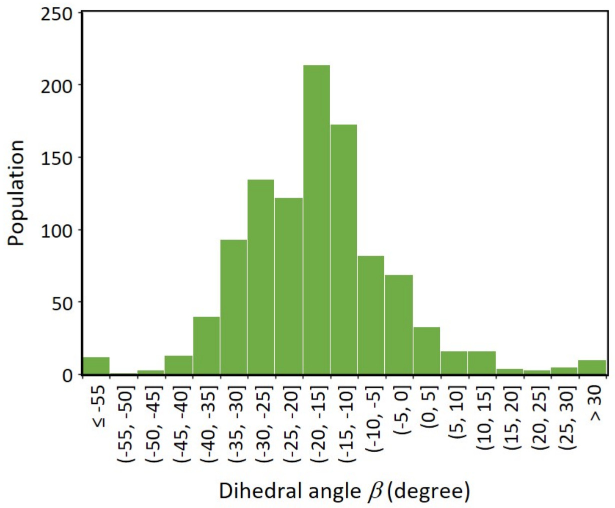

Figure S20: Distributions of the twisting dihedral angle β, as defined in Figure S17, computed along the MD trajectories in water.

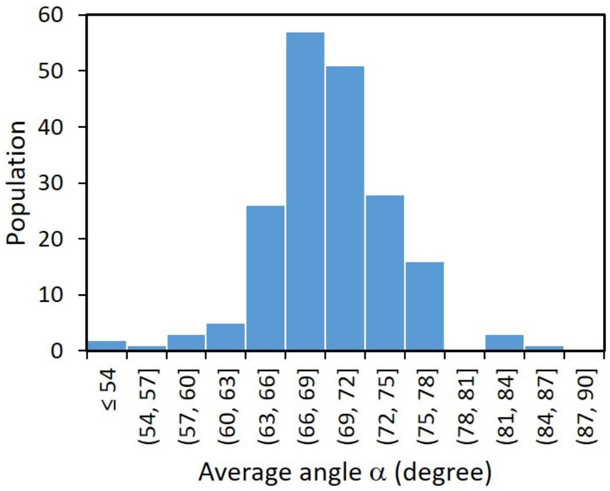

Figure S21: Distributions of the angle α, averaged over the four angles α_1, α_2, α_3 and α_4 (as defined in Figure S17), along the MD trajectories in water. For the sake of clarity, only the values $\leq 90°$ are considered in the distribution.

Table S10: The value of the most relevant inter-monomer coordinates defined in Figure S17 for all the Zn Pc dimers considered in this study. The distances are in Å, while the angles and dihedrals are in degree. The values of the geometrical descriptors for each representative Zn Pc dimer obtained from the cluster analysis were taken from the corresponding frame (shown in parentheses) along the MD trajectory.

Geometrical descriptor	CS1 (frame 585)	CS2 (frame 971)	CS3 (frame 490)	CS4 (frame 217)	OM	DM
distances						
d_{Zn-Zn}	4.210	4.150	4.660	4.530	3.080	4.630
d_{P-P}	3.738	3.393	3.527	3.849	3.301	4.852
angles						
α_1	88.19	84.23	59.90	111.50	108.48	102.85
α_2	96.87	64.29	62.80	116.20	112.94	106.09
α_3	102.31	62.42	87.45	99.20	109.25	105.58
α_4	95.96	72.51	108.39	81.30	93.52	94.97
α_5	90.22	95.02	120.07	64.19	83.38	89.17
α_6	80.48	114.30	116.18	58.60	75.43	82.29
α_7	72.50	117.93	96.53	69.50	82.59	86.49
α_8	78.09	105.14	73.75	96.34	92.62	91.38
dihedral						
β	-15.45	-22.63	-23.24	-17.37	-23.69	-22.69

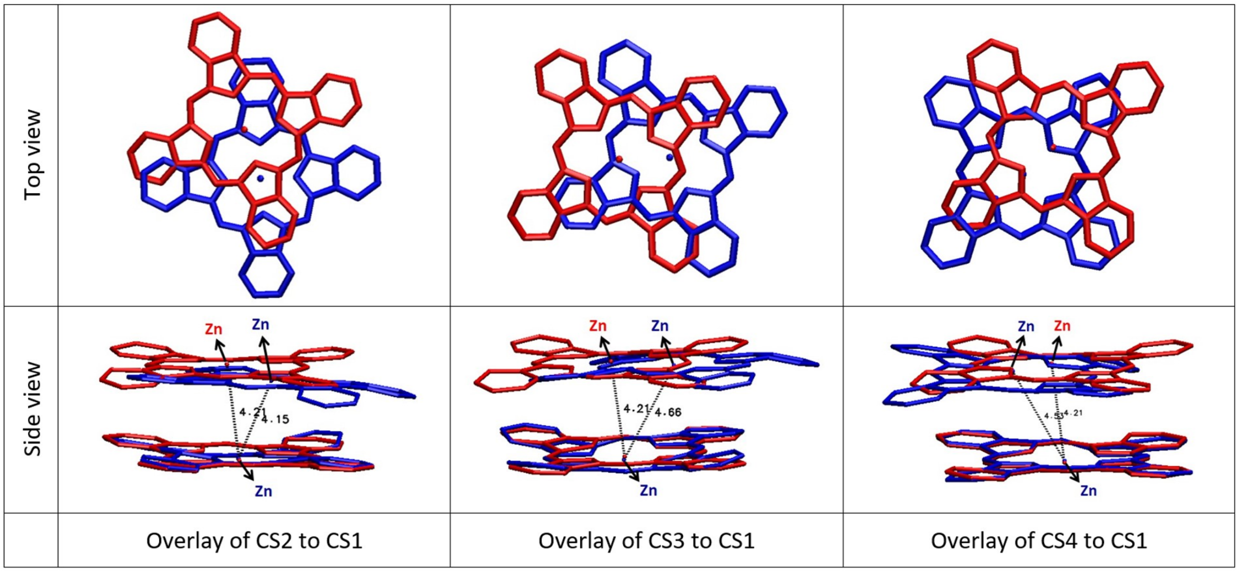

Figure S22: Superimposition of the representative Zn Pc dimer structures of CS2, CS3 and CS4 with that of the CS1, in both top (top panel) and side views (bottom panel). In the top panel, only the upper Zn Pc units are shown.

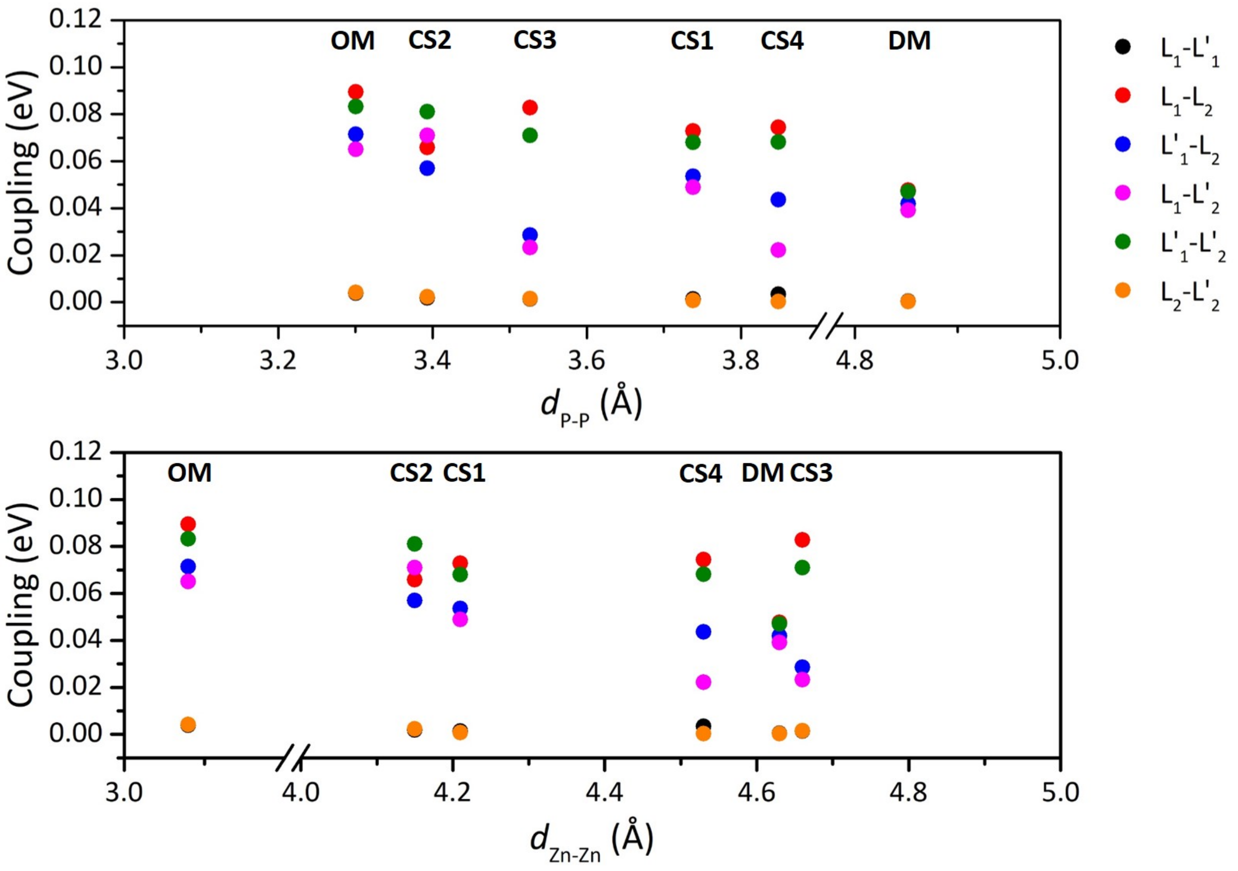

Figure S23: Dependence of the LE-LE interstate couplings with the values of d_{P-P} (top panel) and d_{Zn-Zn} (Å) (bottom panel) for all the Zn Pc dimers considered in this study.

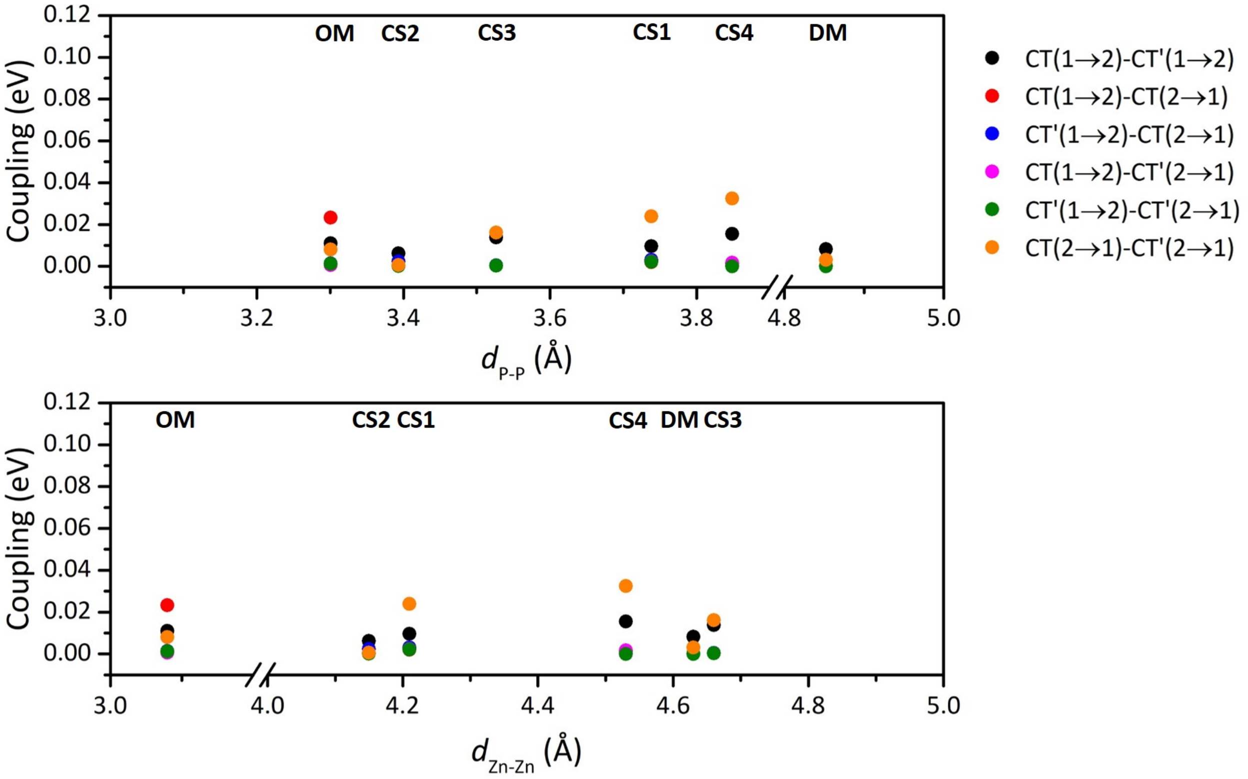

Figure S24: Dependence of the CT-CT interstate couplings with the values of d_{P-P} (top panel) and d_{Zn-Zn} (Å) (bottom panel) for all the Zn Pc dimers considered in this study.

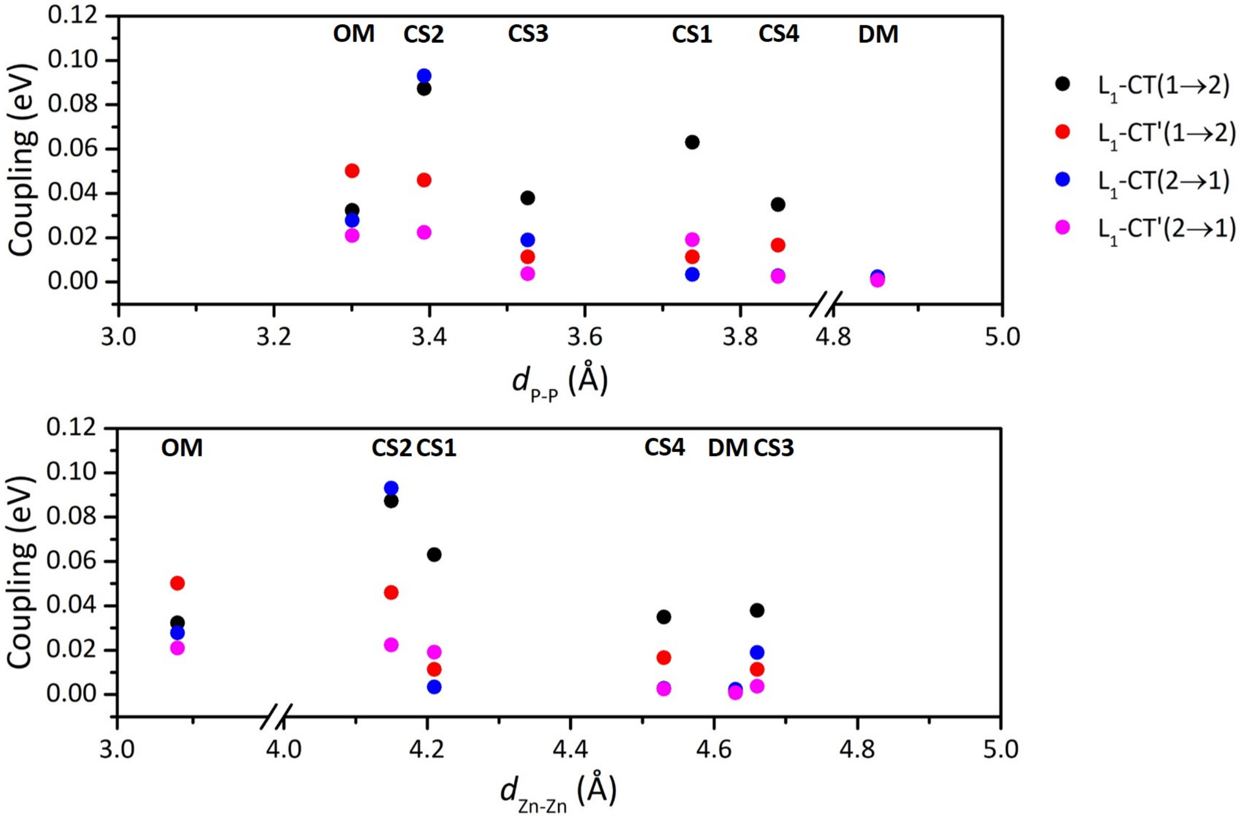

Figure S25: Dependence of the L_1-CT interstate couplings with the values of d_{P-P} (top panel) and d_{Zn-Zn} (Å) (bottom panel) for all the Zn Pc dimers considered in this study.

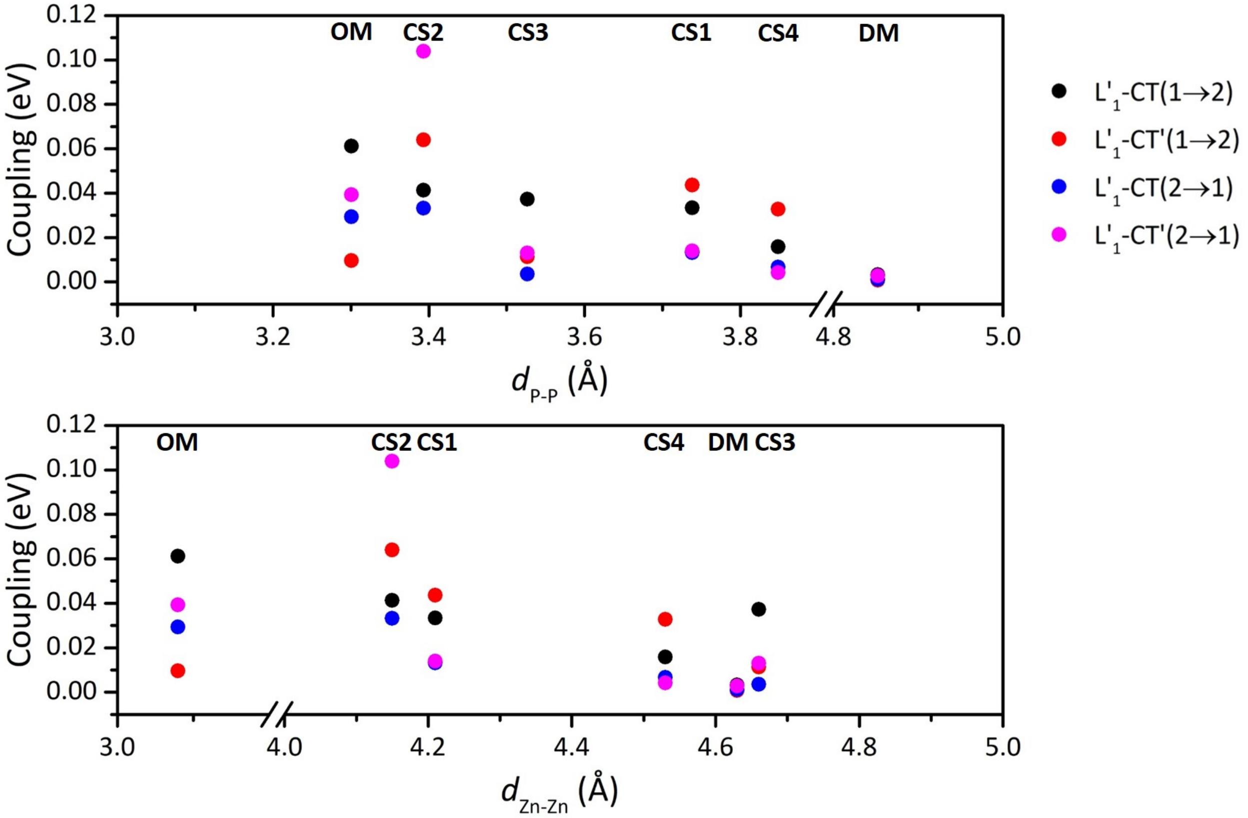

Figure S26: Dependence of the L'_1-CT interstate couplings with the values of d_{P-P} (top panel) and d_{Zn-Zn} (Å) (bottom panel) for all the Zn Pc dimers considered in this study.

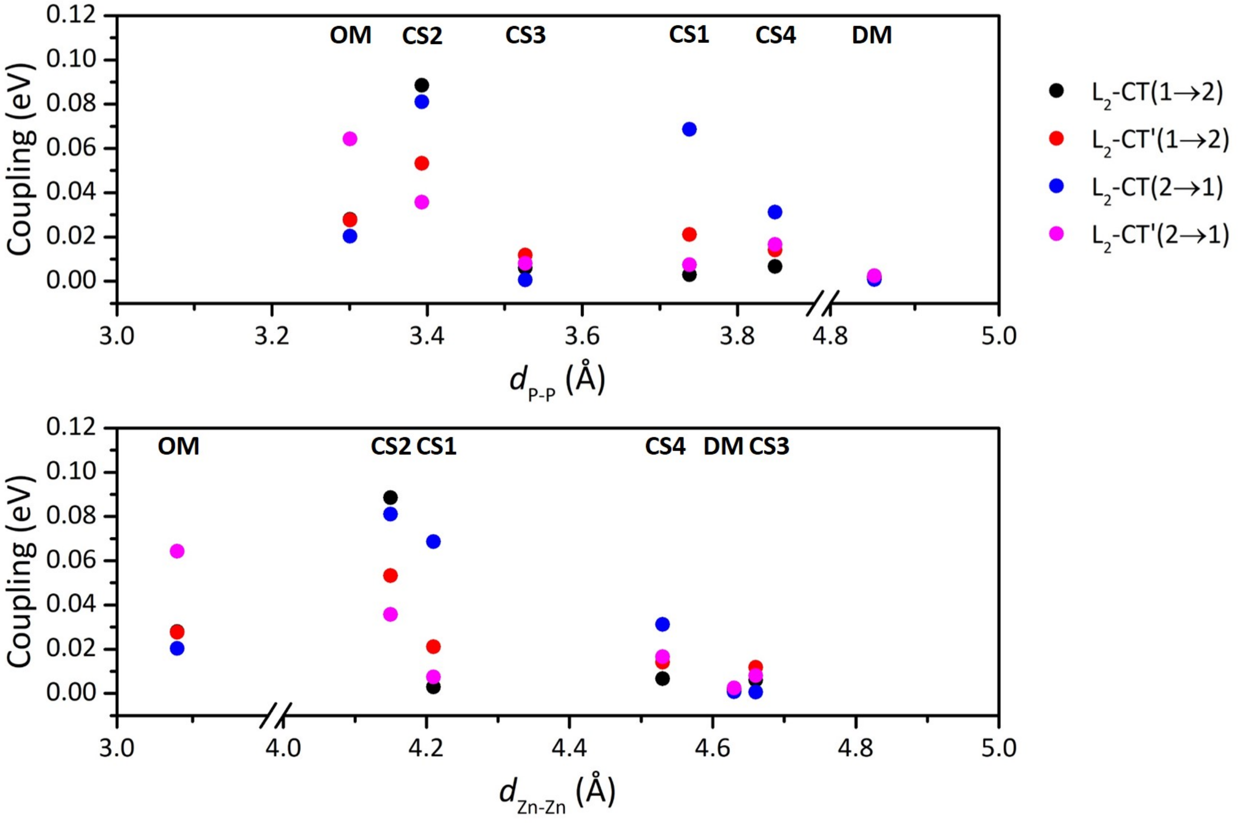

Figure S27: Dependence of the L_2-CT interstate couplings with the values of d_{P-P} (top panel) and d_{Zn-Zn} (Å) (bottom panel) for all the Zn Pc dimers considered in this study.

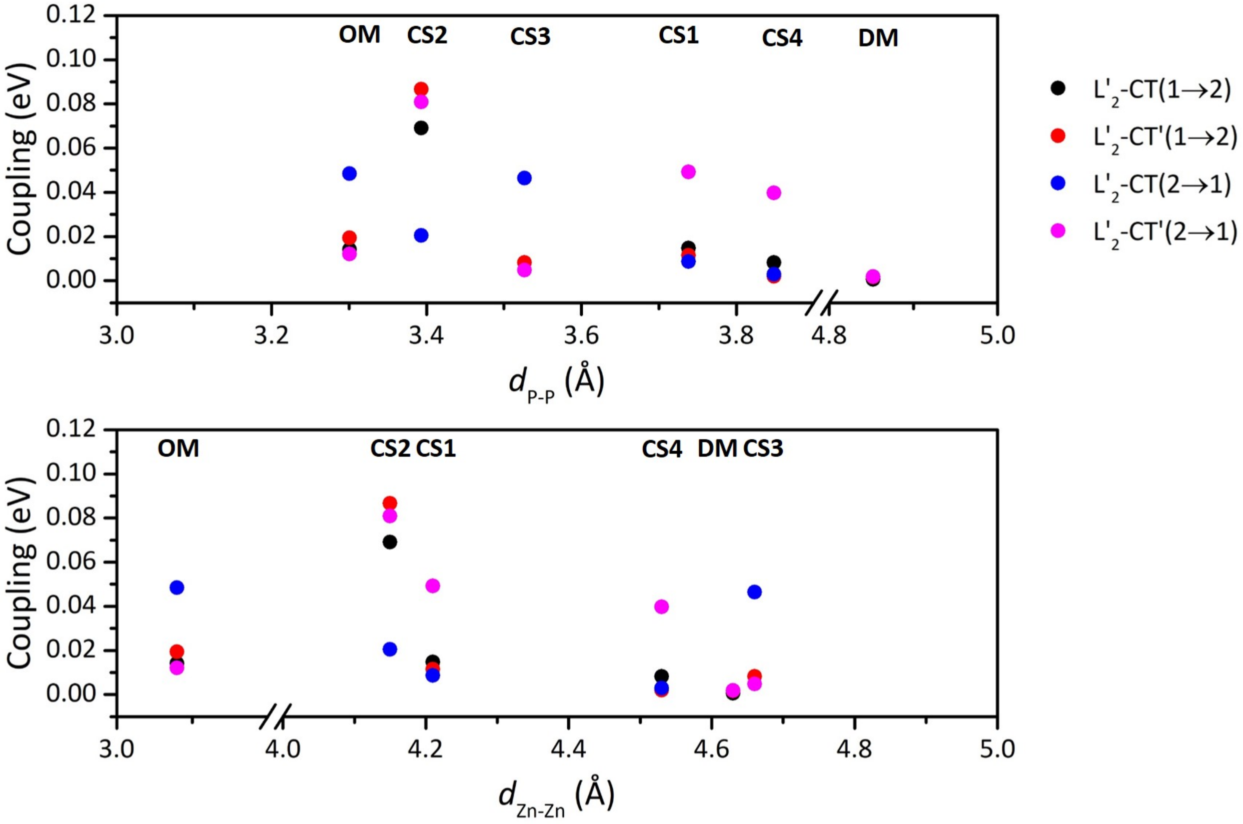

Figure S28: Dependence of the L_2'-CT interstate couplings with the values of d_{P-P} (top panel) and d_{Zn-Zn} (Å) (bottom panel) for all the Zn Pc dimers considered in this study.

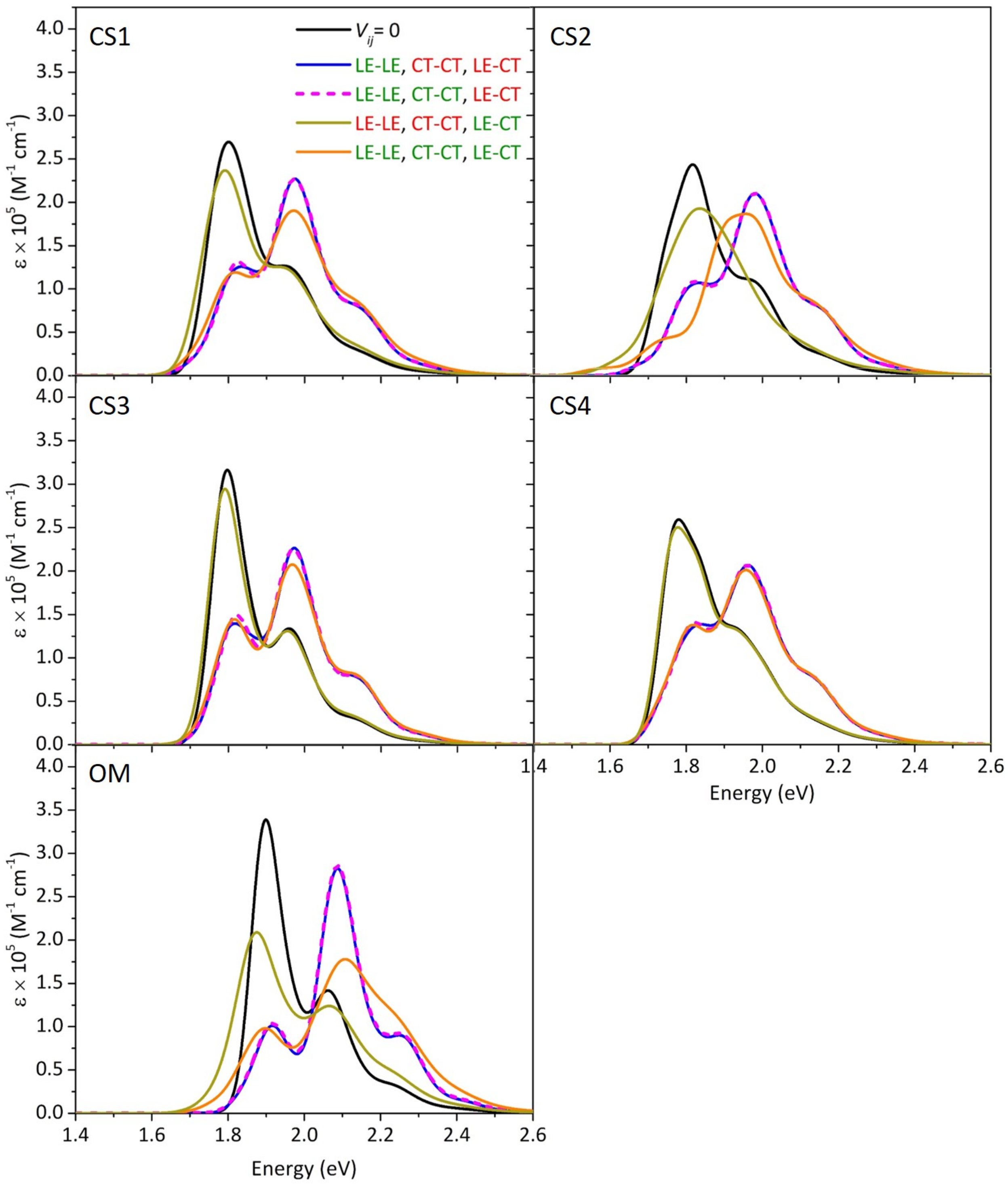

Figure S29: The nonadiabatic vibronic LVC spectra computed for the Zn Pc dimers considered in this study, by including (colored in green) or excluding (colored in red) different contributions from each type of interstate couplings. All vibronic transitions were convoluted with a Gaussian function with HWHM = 0.04 eV. No shift has been applied to the computed spectra.

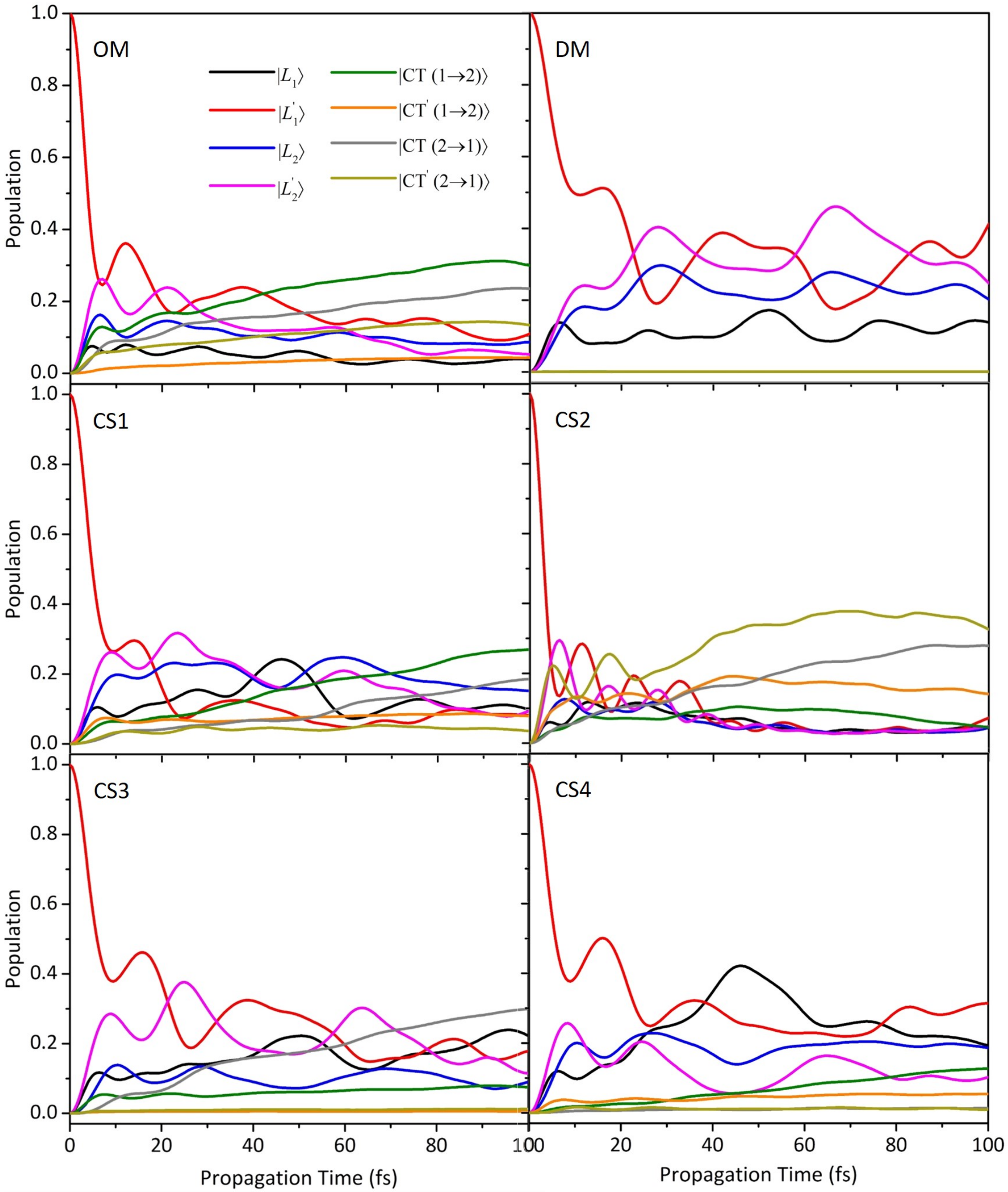

Figure S30: Population dynamics of the diabatic states of all the Zn Pc dimers considered in this study, starting photoexcitation from $|L_1'\rangle$. ML-MCTDH including 30 effective coordinates.

Table S11: Vertical excitation energies of the minima of the diabatic states according to the LVC models parameterized at different dimer structures with TD-CAM-B3LYP/6-31G(d,p)/LR-PCM(water,nonequilibrium). The zero energy value is different for each dimer and corresponds its ground state. All values are in eV.

Structure	$\|L_1\rangle$	$\|L'_1\rangle$	$\|L_2\rangle$	$\|L'_2\rangle$	$\|CT(1\rightarrow 2)\rangle$	$\|CT'(1\rightarrow 2)\rangle$	$\|CT(2\rightarrow 1)\rangle$	$\|CT'(2\rightarrow 1)\rangle$
OM	1.900	1.900	1.896	1.899	1.558	1.573	1.562	1.568
DM	1.873	1.883	1.870	1.873	2.018	2.033	2.013	2.035
CS1	1.771	1.816	1.738	1.777	1.505	1.617	1.535	1.635
CS2	1.700	1.804	1.757	1.808	1.482	1.523	1.411	1.508
CS3	1.758	1.777	1.780	1.787	1.602	1.625	1.500	1.616
CS4	1.743	1.767	1.748	1.822	1.513	1.630	1.590	1.704

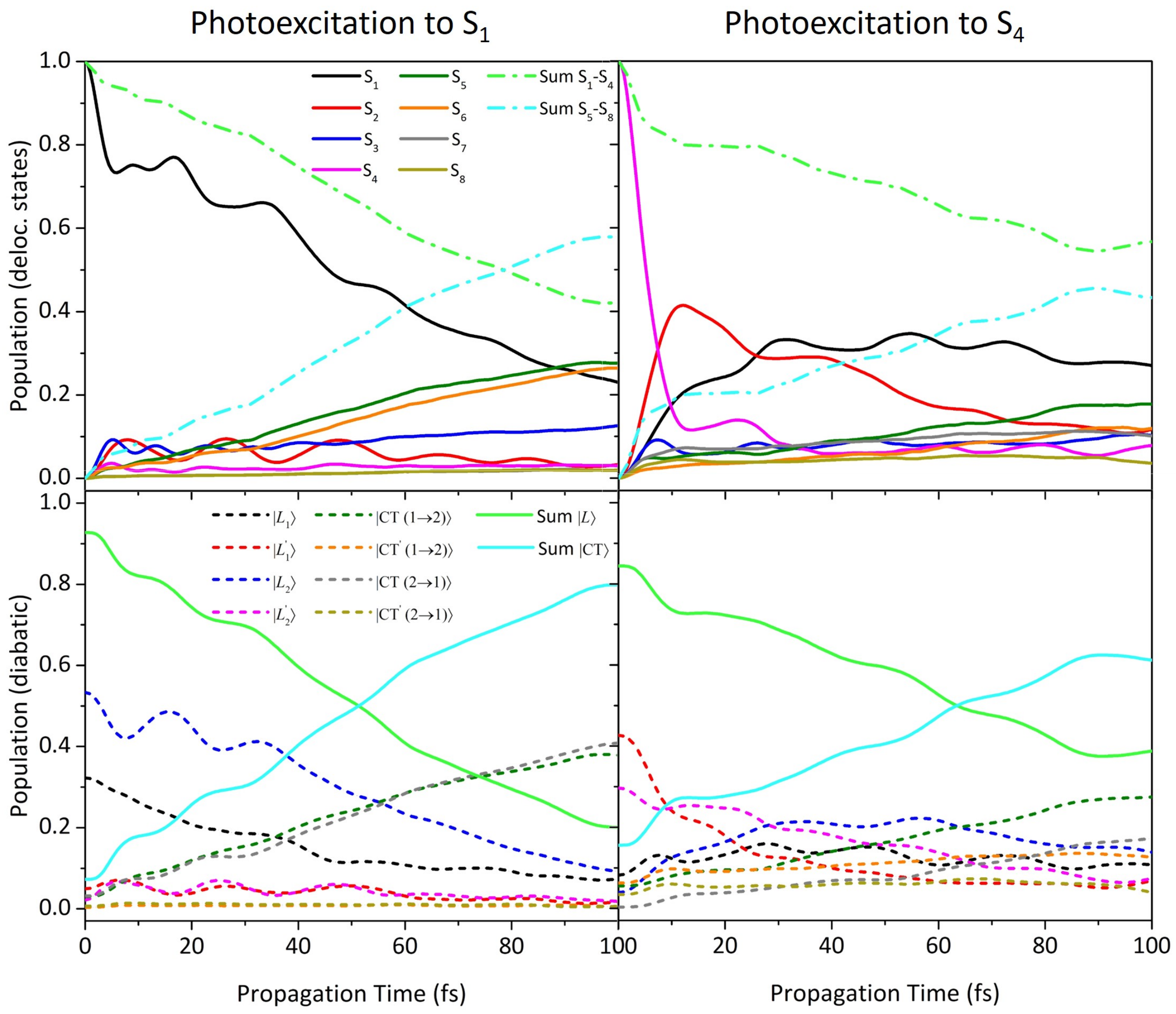

Figure S31: Population dynamics of the delocalized states (S_1-S_8, top) and diabatic local states (LE + CT, bottom) of CS1 Zn Pc dimer after photoexcitation to the lowest energy dark exciton state S_1 (left) or to the second bright adiabatic state S_4 (right). ML-MCTDH propagations including 30 effective coordinates. The results after a photoexcitation to the bright state S_3 are reported in Figure 10 in the main text.

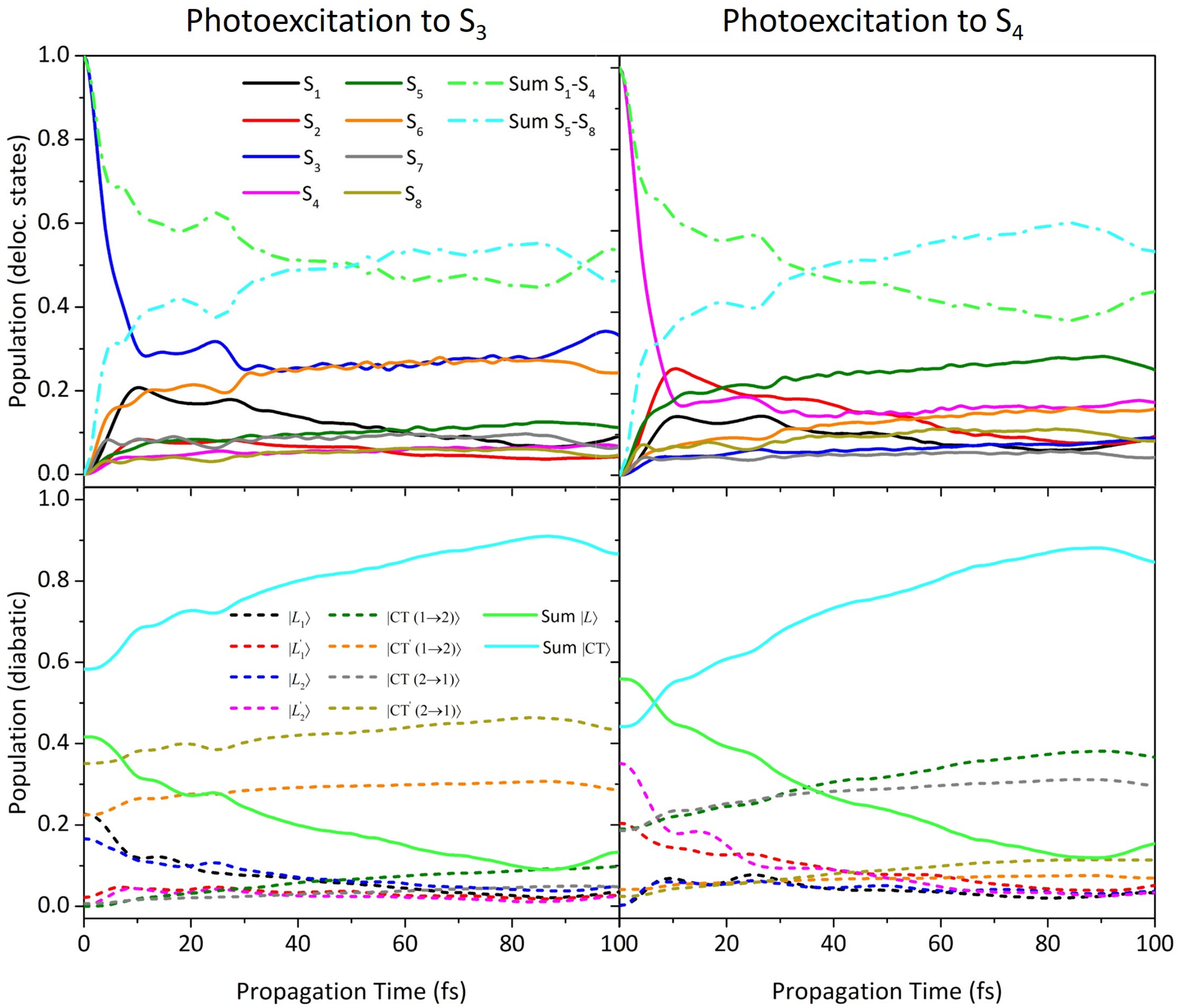

Figure S32: Population dynamics of the delocalized states (S_1-S_8, top) and diabatic local states (LE + CT, bottom) of OM Zn Pc dimer after photoexcitation to the bright adiabatic states S_3 (left) or S_4 (right). ML-MCTDH propagations including 30 effective coordinates.

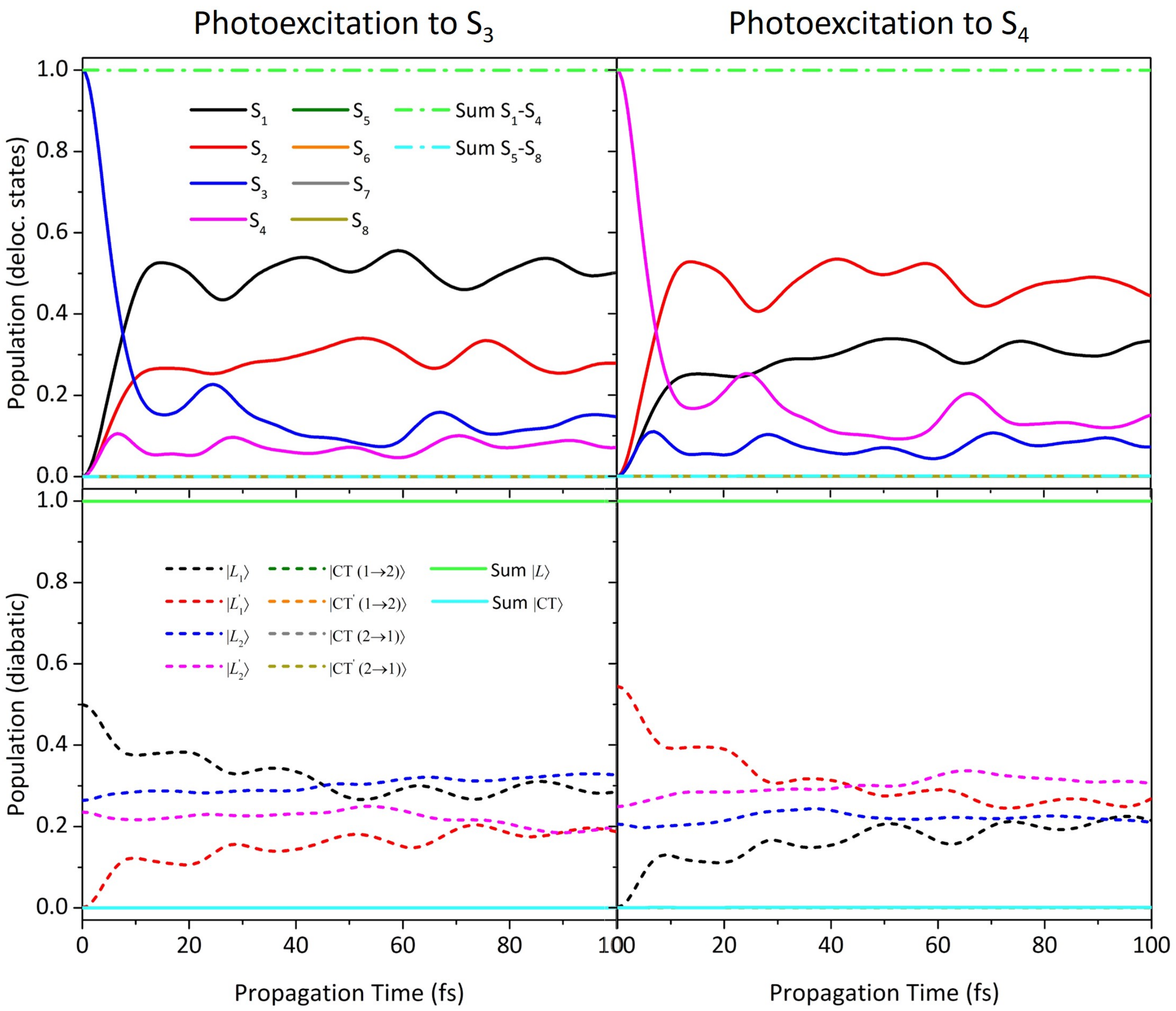

Figure S33: Population dynamics of the delocalized states (S_1-S_8, top) and diabatic local states (LE + CT, bottom) of DM Zn Pc dimer after photoexcitation to the bright adiabatic states S_3 (left) or S_4 (right). ML-MCTDH propagations including 30 effective coordinates.

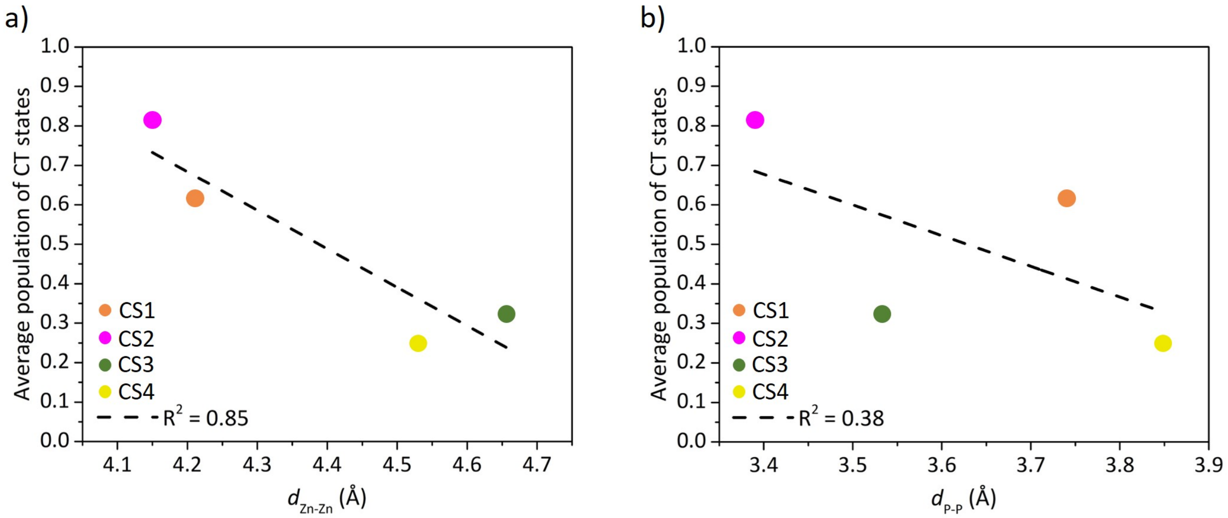

Figure S34: Linear dependence of the total population of the CT states after 100 fs, averaged over initial photoexcitation from the $|L_1\rangle$ and $|L_1'\rangle$ states, on the d_{Zn-Zn} (left panel) and d_{P-P} (right panel) stacking distances for the CS dimers obtained from the MD cluster analysis.

References

(S1) Yaghoubi Jouybari, M.; Liu, Y.; Improta, R.; Santoro, F. Ultrafast Dynamics of the Two Lowest Bright Excited States of Cytosine and 1-Methylcytosine: A Quantum Dynamical Study. *J. Chem. Theory Comput.* **2020**, *16*, 5792–5808.

(S2) Green, J. A.; Asha, H.; Santoro, F.; Improta, R. Excitonic Model for Strongly Coupled Multichromophoric Systems: The Electronic Circular Dichroism Spectra of Guanine Quadruplexes as Test Cases. *J. Chem. Theory Comput.* **2020**, *17*, 405–415.
